# A comparative genomics study of neuropeptide genes in the cnidarian subclasses Hexacorallia and Ceriantharia

**DOI:** 10.1186/s12864-020-06945-9

**Published:** 2020-09-29

**Authors:** Thomas L. Koch, Cornelis J. P. Grimmelikhuijzen

**Affiliations:** Section for Cell and Neurobiology, Department of Biology, University of Copenhagen, Universitetsparken 15, DK-2100 Copenhagen, Denmark

**Keywords:** Genome, Transcriptome, Functional genomics, Phylogenomics, Neuropeptide, Nervous system, Cnidaria, Sea anemone, Coral, Reef restoration

## Abstract

**Background:**

Nervous systems originated before the split of Proto- and Deuterostomia, more than 600 million years ago. Four animal phyla (Cnidaria, Placozoa, Ctenophora, Porifera) diverged before this split and studying these phyla could give us important information on the evolution of the nervous system. Here, we have annotated the neuropeptide preprohormone genes of twenty species belonging to the subclass Hexacorallia or Ceriantharia (Anthozoa: Cnidaria), using thirty-seven publicly accessible genome or transcriptome databases. Studying hexacorals is important, because they are versatile laboratory models for development (e.g., *Nematostella vectensis*) and symbiosis (e.g., *Exaiptasia diaphana*) and also are prominent reef-builders.

**Results:**

We found that each hexacoral or ceriantharian species contains five to ten neuropeptide preprohormone genes. Many of these preprohormones contain multiple copies of immature neuropeptides, which can be up to 50 copies of identical or similar neuropeptide sequences. We also discovered preprohormones that only contained one neuropeptide sequence positioned directly after the signal sequence. Examples of them are neuropeptides that terminate with the sequence RWamide (the Antho-RWamides). Most neuropeptide sequences are N-terminally protected by pyroglutamyl (pQ) or one or more prolyl residues, while they are C-terminally protected by an amide group. Previously, we isolated and sequenced small neuropeptides from hexacorals that were N-terminally protected by an unusual L-3-phenyllactyl group. In our current analysis, we found that these N-phenyllactyl-peptides are derived from N-phenylalanyl-peptides located directly after the signal sequence of the preprohormone. The N-phenyllactyl- peptides appear to be confined to the hexacorallian order Actiniaria and do not occur in other cnidarians. On the other hand, (1) the neuropeptide Antho-RFamide (pQGRFamide); (2) peptides with the C-terminal sequence GLWamide; and (3) tetrapeptides with the X_1_PRX_2_amide consensus sequence (most frequently GPRGamide) are ubiquitous in Hexacorallia.

**Conclusions:**

We found GRFamide, GLWamide, and X_1_PRX_2_amide peptides in all tested Hexacorallia. Previously, we discovered these three neuropeptide classes also in Cubozoa, Scyphozoa, and Staurozoa, indicating that these neuropeptides originated in the common cnidarian ancestor and are evolutionarily ancient. In addition to these ubiquitous neuropeptides, other neuropeptides appear to be confined to specific cnidarian orders or subclasses.

## Background

Four animal phyla (Cnidaria, Placozoa, Ctenophora, Porifera) occupy an evolutionarily interesting position, because they diverged from other animal lineages before the split of Proto- and Deuterostomia, more than 600 billion years ago [1]. Extant members of these four phyla, therefore, are an important resource for studying the early evolution of animal development, cell-to-cell signaling, and of organs and tissues like the brain and neuro-endocrine system.

The focus of this paper is on nervous systems and neuropeptides from Cnidaria. Animals belonging to this phylum often have a life cycle including a planula larva, a polyp, and a medusa stage. Anatomically, the nervous system of cnidarians consists of a nerve net, which sometimes is condensed to form a nerve plexus in the head or foot regions of polyps, or in the mouth regions of medusae. These condensations can also lead to giant axons in tentacles from polyps, or nerve rings around the bell margins of medusae [2–9]. The nervous systems of cnidarians are strongly peptidergic and a large number of neuropeptides have been isolated and sequenced from these animals (reviewed in [9–13]). Many cnidarians are transparent and the use of whole-mount animals and antibodies raised against their neuropeptides have enabled scientists to visualize the overall organization of the cnidarian nervous systems in unmatched detail [5–7, 9–11, 14–17].

Cnidarian neuropeptides play a role in development, metamorphosis, reproduction, feeding, muscle contraction, and probably many other processes [9–11, 13, 18–23].

We and other research groups have cloned the preprohormones for many of the sequenced cnidarian neuropeptides. This work showed that these preprohormones contain multiple immature (=unprocessed) neuropeptide copies, which can be up to 38 copies per preprohormone [9–11, 24–30]. At the C-terminal side of each immature neuropeptide sequence, we found the classical KR, RR, or R sequences, which are cleaving signals for the prohormone convertases PC1/PC3 and PC2. These prohormone convertase cleavage sites were always preceded by G residues that can be converted into C-terminal amide groups by peptidylglycine monooxygenase [31–33]. At the N-termini of the immature neuropeptide sequences, however, and in contrast to the situation in mammals, basic residues were lacking and, instead, acidic residues (D or E), hydrophilic residues (S or T), or other amino acid sequences occurred [9–11, 24–30]. From these findings we concluded that the N-termini of immature cnidarian neuropeptide sequences are processed by unspecific aminopeptidases [10, 11]. We proposed that such peptidases might cleave until the N-terminal neuropeptide protecting groups (Q, X-P, or X-P-P sequences) would be reached, stopping further processing [34]. An endoproteinase might also be involved in the propeptide cleavage around E and D residues [10, 11]. Therefore, this unorthodox and unspecific N-terminal processing makes it sometimes difficult to predict the precise N-terminal sequences of mature neuropeptides, when annotating cnidarian preprohormones from genomic or transcriptomic databases [19, 34]. It is interesting that immature neuropeptides from Placozoa appear to be processed by the same unorthodox N-terminal processing enzymes as the neuropeptides from Cnidaria [35], suggesting that this type of N-terminal peptide processing is characteristic for early neuroendocrine systems.

**Fig. 1:**
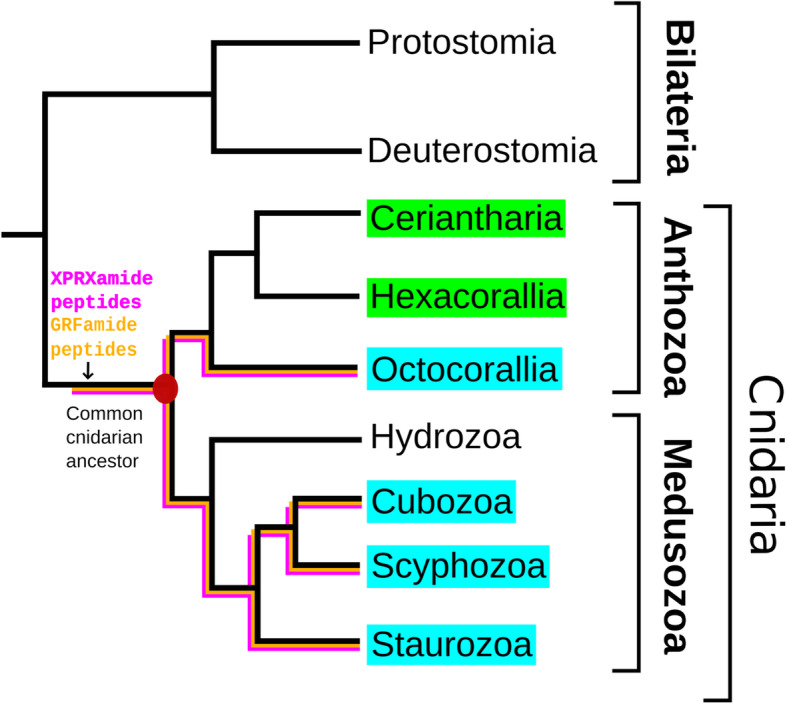
Schematic drawing showing the phylogenetic positions of the subclasses Ceriantharia, Hexacorallia and Octocorallia and the classes Hydrozoa, Cubozoa, Scyphozoa, and Staurozoa. Cnidaria are a sister group to Bilateria. The figure also shows that X_1_PRX_2_amide and GRFamide peptides are present in all testet Octocorallia, Cubozoa, Scyphozoa, and Staurozoa [34]. In the current paper, we are investigating these and other neuropeptides in Ceriantharia and Hexacorallia. Modified from [34]

Most of the cnidarian neuropeptides have been isolated from just a few model cnidarians, such as the fresh water polyp *Hydra magnipapillata*, the hydrozoan medusa *Polyorchis penicillatus*, the scyphomedusan *Cyanea lamarckii*, and the sea anemones *Anthopleura elegantissima*, *Calliactis parasitica*, and *Nematostella vectensis* [9–13, 21, 36]. It was, therefore, unclear whether neuropeptides were present in all cnidarians and, if so, what the structures of these neuropeptides were. This question, however, can now be solved, because the genomes from many cnidarians have been sequenced and numerous cnidarian transcriptomes are also available [37–51]. Last year, we developed a bioinformatics tool that could analyze cnidarian genomes and transcriptomes for the presence of neuropeptide preprohormones genes [37]. Using this script, we analyzed all available genome and transcriptome databases from Cubozoa, Scyphozoa, Staurozoa (belonging to the subphylum Medusozoa) and Octocorallia (belonging to the class Anthozoa) for the presence of neuropeptide preprohormones [34, 37]. Our analyses showed that all investigated cnidarians possessed a GRFamide neuropeptide: pQGRFamide in Octocorallia; pQWLRGRFamide in Cubozoa and Scyphozoa; and pQFLRGRFamide in Staurozoa. Furthermore, we found that all investigated species produced RPRSamide or a related neuropeptide sequence (consensus sequence X_1_PRX_2_amide, where X_2_ was S, A, or G) [34, 37]. Because the Cubozoa, Scyphozoa, Staurozoa, and Octocorallia have their closest evolutionary root in the common ancestral cnidarian (Fig. 1), we concluded that the GRFamide and X_1_PRX_2_amide are primordial neuropeptides that evolved in the common cnidarian ancestor [34]. In addition to these ubiquitous neuropeptides, we also found 2 to 3 neuropeptide genes that were confined to a certain cnidarian class. We assumed that these neuropeptides originated later in evolution and served class-specific functions [34].

In our previous analysis, we used the anthozoan subclasss Octocorallia as an out-group, which made it possible to draw conclusions on the early origins of the GRFamide and X_1_PRX_2_amide neuropeptides [34] (Fig. 1). The Anthozoa, however, consist of three subclasses (Fig. 1). In the current paper, therefore, we have decided also to analyze the subclasses Hexacorallia and Ceriantharia. This analysis was especially needed, because, thirty years ago, we isolated and sequenced many neuropeptides from sea anemones (Hexacorallia) [9–11] that could not be identified in Octocorallia [34]. Some of these neuropeptides from sea anemones had the C-terminal sequence RPamide, such as LPPGPLPRPamide, GPHypSLFRPamide (Hyp = hydroxyproline), pQNFHLRPamide, or pQVKLYRPamide [9–11, 52, 53]. Other neuropeptides had the C-terminal sequence RWamide, such as pQSLRWamide, or pQGLRWamide [9–11, 54, 55]. We also isolated and sequenced neuropeptides from sea anemones that had the unusual L-3-phenyllactyl residue as an N-terminal protecting group, such as L-3-phelac-LRNamide, L-3-phelac-YRIamide, or L-3-phelac-FKAamide [9–11, 56–58]. For the last group of neuropeptides, the identification of their preprohormones would be especially rewarding, because we have not been able to clone these preprohormones using the degenerate primer PCR techniques of thirty years ago. These preprohormones would also inform us on the biosynthesis of the N-terminal L-3-phenyllactyl group: Would this group originate from an N-terminal phenylalanine residue or would it be attached to the unprotected tripeptide by a, so far unknown, N-acyltranferase [9, 10, 56]?

**Fig. 2:**
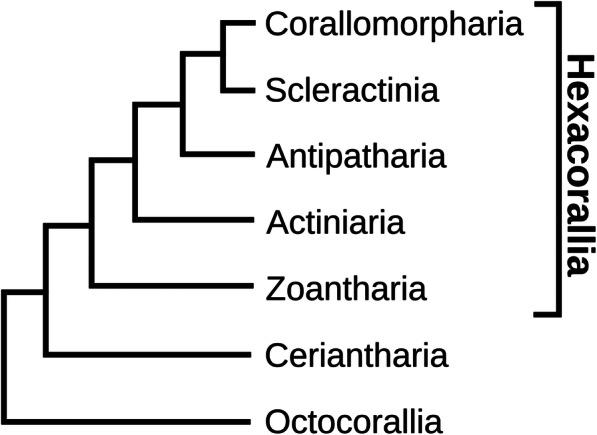
Phylogenetic relationships between the various Hexacorallia orders and the subclasses Ceriantharia and Octocorallia. All hexacoral orders were studied in this paper except for the Antipatharia, which has no species with a sequenced genome or transcriptome. This figure is based on data from [59, 60]

In our current paper, we have identified the neuropeptide preprohormones from nineteen species belonging to five different orders of Hexacorallia and from one species belonging to the anthozoan subclass Ceriantharia (Fig. 1, Fig. 2, and Table 1). This large number of hexacorallian species also includes the model sea anemones *N. vectensis*, and *E. diaphana*, and the model coral *Acropora millepora* (Table 1). In our study, we have clarified many of the questions raised in the preceding paragraph.

**Table 1:** Accession numbers for the different databases used

**Species**	**Subclass**	**Order**	**Database type**	**Accession number**
Anemonia viridis	Hexacorallia	Actiniaria	WGS	OCZR00000000.1
Anemonia viridis	Hexacorallia	Actiniaria	TSA	BBLT00000000.1 GHCD00000000.1
Anthopleura elegantissima	Hexacorallia	Actiniaria	TSA	GBXJ00000000.1 GBYC00000000.1
Exaiptasia diaphana	Hexacorallia	Actiniaria	WGS	LJWW00000000.1
Exaiptasia diaphana	Hexacorallia	Actiniaria	TSA	JV077153-JV134524
Nematostella vectensis	Hexacorallia	Actiniaria	WGS	ABAV00000000.1
Nematostella vectensis	Hexacorallia	Actiniaria	TSA	HADP00000000.1 HADN00000000.1
Phymanthus crucifer	Hexacorallia	Actiniaria	WGS	WUCR0000000.1
Scolanthus callimorphus	Hexacorallia	Actiniaria	TSA	GGGE00000000.1
Amplexidiscus fenestrafer	Hexacorallia	Corallimorpharia	WGS	PRJNA354436
Corynactis australis	Hexacorallia	Corallimorpharia	TSA	GELM00000000.1
Discosoma sp.	Hexacorllia	Corallimorpharia	WGS	PRJNA35449
Ricordea yuma	Hexacorallia	Corallimorpharia	TSA	GELN00000000.1
Acropora digitifera	Hexacorallia	Scleractinia	WGS	BACK00000000.2
Acropora millepora	Hexacorallia	Scleractinia	WGS	QTZP00000000.1
Acropora millepora	Hexacorallia	Scleractinia	TSA	GHGU00000000.1 GHGM00000000.1 GHGS00000000.1 GHGO00000000.1 GHGH00000000.1 GHGT00000000.1 GHGN00000000.1 GHGQ00000000.1
Montipora capitata	Hexacorallia	Scleractinia	WGS	RDEB00000000.1
Montipora capitata	Hexacorallia	Scleractinia	TSA	GEFRO00000000.1
Orbicella faveolata	Hexacorallia	Scleractinia	WGS	MZGG00000000.1
Pocillopora damicornis	Hexacorallia	Scleractinia	WGS	RCHS00000000.1
Pocillopora damicornis	Hexacorallia	Scleractinia	TSA	GEFF00000000.1
Porites rus	Hexacorallia	Scleractinia	WGS	OKRP00000000.1
Stylophora pistillata	Hexacorallia	Scleractinia	WGS	LSTM00000000.1
Stylophora pistillata	Hexacorallia	Scleractinia	TSA	GARY00000000.1
Protopalythoa variabilis	Hexacorallia	Zoantharia	TSA	GCVI00000000.1
Zoanthus sp.	Hexacorallia	Zoantharia	TSA	GGTW00000000.1
Pachycerianthus borealis	Ceriantharia	Spirularia	TSA	HAGY00000000.1

Last year, a paper was published that described the annotation and sequencing of neuropeptides in *N. vectensis*, using tandem mass spectrometry [36]. In our current study, we have discovered several additional neuropeptides in *N. vectensis*. Thus, our study not only gives a global overview of neuropeptide genes present in Hexacorallia, but it also supplies the scientific community with a more complete inventory of the neuropeptides present in several hexacoral laboratory models.

## Results

### Annotations of genes and transcripts coding for neuropeptide preprohormones

Table 1 gives the accession numbers of the genome and transcriptome databases that we used for the analyses of the various members of Hexacorallia and Ceriantharia. These analyses were carried out, using a script described in [37] that was based on the presence of two or more similar neuropeptide copies on a neuropeptide preprohormone (see also Methods). However, to avoid the exclusion of single copy neuropeptide preprohormones, we also applied TBLASTN and a related software program named Regex (Regular expression). In the following sections, we identify neuropeptide preprohormones in species belonging to the orders Actinaria (sea anemones), Scleractinia (stony corals), Corallimorpharia (hexacorallians resembling stony corals, but lacking a stony skeleton), Zoantharia (sand-incorporating corals), and the subclass Ceriantaria (tube-dwelling anemones). Figure 2 gives the most recently proposed phylogenetic relationships between these orders and the subclasses Ceriantharia, and Octocorallia [59, 60].

### Preprohormones in Actiniaria containing multiple neuropeptide copies

We annotated six neuropeptide gene families in Actinaria (indicated as neuropeptide family #1to #6 in Table 2 and Table 3) that coded for preprohormones with multiple neuropeptide copies. The first family consists of preprohormones that produce a large number of GPRGamide or APRGamide neuropeptides (Table 2, neuropeptide family #1). In the sea anemone *A. elegantissima*, we identified an incomplete preprohormone, containing 19 copies of GPRGamide; in *A. viridis* two non-overlapping preprohormone fragments, one containing 17 and another 16 copies of GPRGamide; in *N. vectensis* an incomplete preprohormone, containing 15 copies of APRGamide and 13 copies of GPRGamide; in *P. crucifer* a complete preprohormone, containing 19 copies of GPRGamide; in *S. callimorphus* a preprohormone fragment, containing 3 copies of APRGamide; and in *E. diaphana* and incomplete preprohormone containing 23 copies of GPRPamide. The amino acid sequences of these GPRGamide preprohormones are given in Additional file 1.

**Table 2:**
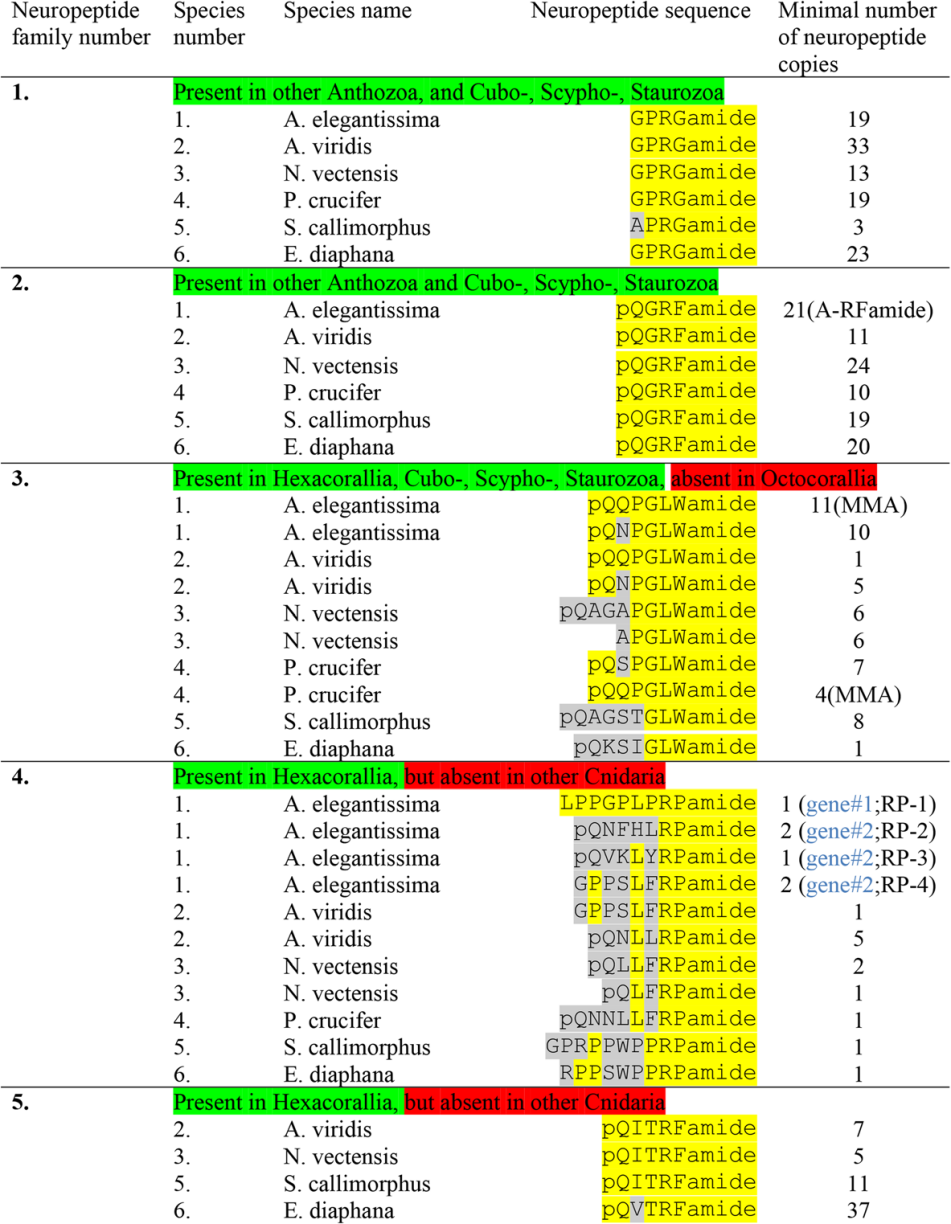
Neuropeptide families identified in six species of Actiniaria: *Anthopleura elegantissima*, *Anemonia viridis*, *Nematostella vectensis*, *Phymanthus crucifer*, *Scolanthus callimorphus*, and *Exaiptasia diaphana*. Only those neuropeptides that have multiple identical or similar copies on their preprohormones are listed here and from them only those with the highest copy numbers are given. The isolated and sequenced neuropeptides from A. elegantissima are abbreviated: A-RFamide (=Antho-RFamide); MMA (= Metamorphosin A); RP-1, -2, -3, -4 (= Antho-RPamide-1, -2, -3, -4). If more than one gene codes for the peptides, this is highlighted in blue in the last row in parentheses. The amino acid sequences of the preprohormones are shown in Additional files 1 to Additional files 5

**Table 3:**
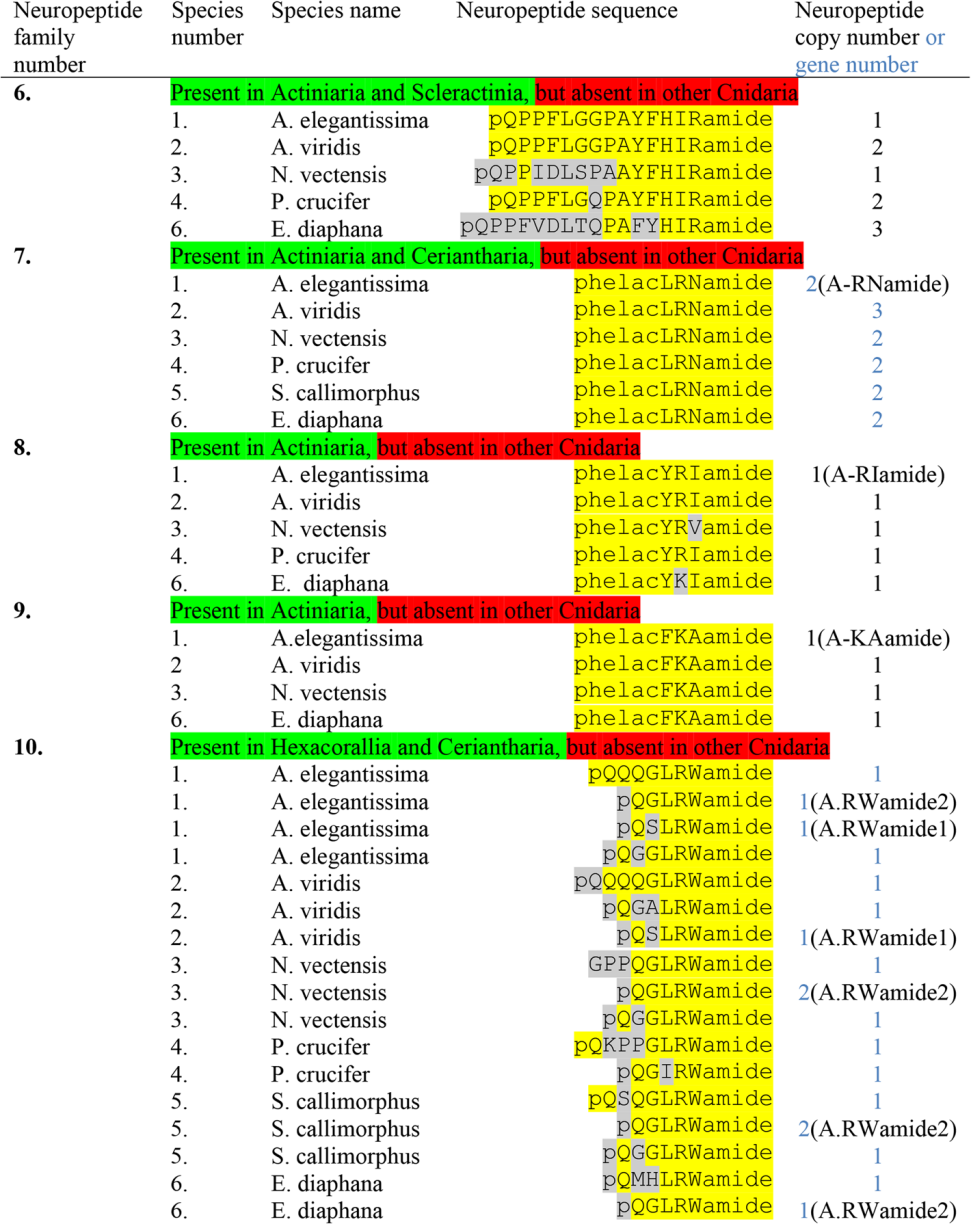
Neuropeptide families #6 to # 10 identified in six species of Actiniaria: *A. elegantissima*, *A. viridis*, *N. vectensis*, *P. crucifer*, *S. callimorphus*, and *E.diaphana*. This table is a continuation of Table 2. Neuropeptide family #6 has multiple copy preprohormones, while neuropeptide families #7 to #10 have single copy preprohormones. The isolated and sequenced neuropeptides from A. elegantissima are abbreviated: A-RNamide (= Antho-RNamide), A-RIamide (= Antho-RIamide); A-KAamide (= Antho-KAamide); A.RWamide1 and 2 (= Antho-RWamide-1 and -2). On some occasions, there are two or more genes coding for the same neuropeptide, which is indicated (highlighted in blue) in the last column. The preprohormone amino acid sequences are shown in Additional file 6 to Additional file 10

The second family (Table 2) consists of preprohormones that all contain a large number of pQGRFamide neuropeptides. These neuropeptides have been isolated and sequenced previously from several anthozoans, including *A. elegantissima* and named Antho-RFamide [9–11, 61]. We have also cloned the Antho-RFamide preprohormones [9–11, 24, 25]. The complete, cloned and published Antho-RFamide preprohormone from *A. elegantissima* contains 21 copies of Antho-RFamide [25]. This complete sequence is given in Additional file 2. In the *A. elegantissima* transcriptome that we investigated, however, we could only recover a small preprohormone fragment, containing 5 Antho-RFamide copies (Additional file 2). In the sea anemone *A. viridis*, an Antho-RFamide preprohormone fragment was identified, containing 11 copies of Antho-RFamide; in *N. vectensis* an incomplete preprohormone, containing 24 copies of Antho-RFamide; in *P. crucifer* a preprohormone fragment, containing 10 copies of Antho-RFamide; in *S. callimorphus* an incomplete preprohormone, containing 19 copies of Antho-RFamide; and in *E. diaphana* an incomplete preprohormone, containing 20 copies of Antho-RFamide (Table 2, Additional file 2).

In the N-terminal parts of all sea anemone Antho-RFamide preprohormones, we found 8–11 repetitive amino acid sequences, each of them 8 residues in length and many having the QFWKGRFS sequence that possibly could represent neuropeptide sequences (highlighted in yellow in Additional file 2). Because multiple copies of immature Antho-RFamide (QGRFGRE, see Additional file 2) are also present on the same prohormone, we assume that the cnidarian prohormone convertase will only cleave between R and E, but not between the R and F residues, which would destroy the Antho-RFamide sequences. Therefore, the N-terminal QFWKGRFS sequences probably do not have a cleavage site for the cnidarian prohormone convertase. Because these sequences also lack a C-terminal amidation signal (G), it is difficult to propose any mature neuropeptide structures for them.

The third neuropeptide family (Table 2) consists of peptides that have the C-terminal sequence GLWamide. These neuropeptides include the isolated and sequenced *A. elegantissima* neuropeptide Metamorphosin A (MMA), which has the sequence pQQPPGLWamide. MMA induces metamorphosis in planula larvae from the colonial hydrozoan *Hydractinia echinata* [21] and the sea anemone *N. vectensis* [62]. We have cloned the MMA preprohormone from *A. elegantissima* and found that it contains 11 copies of MMA together with 10 copies of pQNPGLWamide, 8 copies of pQPGLWamide and several copies of other related neuropeptide sequences [27]. The sequence of this cloned MMA preprohormone from *A. elegantissima* is given in Additional file 3. In the tested transcriptomes from *A. elegantissima* (Table 1), we could identify an MMA preprohormone fragment that contained three copies of pQNPGLWamide and 5 copies of pQPGLWamide, but no MMA (Additional file 3). This preprohormone fragment corresponds to the N-terminal half of the complete, cloned MMA preprohormone (Additional file 3) [27]. In *A. viridis*, we found several non-overlapping MMA preprohormone fragments, containing 1 copy of MMA and 5 copies of the related peptide pQNPGLWamide (Table 2, Additional file 3). In *N. vectensis*, we identified a preprohormone fragment, containing 6 copies of pQAGAPGLWamide and 6 copies of APGLWamide, but we could not discover true MMA sequences (Table 2, Additional file 3). In *P. crucifer*, we identified two preprohormone fragments, containing altogether 7 copies of pQSPGLWamide, 4 copies of MMA, and 5 other GLWamide peptides. In *C. callimorphus*, we identified a few non-overlapping preprohormone fragments, containing 8 copies of pQAGSTGLWamide and several other related sequences, but no genuine MMA. In *E. diaphana*, we found a preprohormone fragment, containing 1 copy of pQKSIGLWamide and several other GLWamide peptides (Table 2, Additional file 3).

The fourth class of neuropeptides has the carboxyterminus RPamide (Table 2). Previously, we have isolated and sequenced various RPamide neuropeptides from *A. elegantissima*, which had the sequences LPPGPLPRPamide (Antho-RPamide-1), pQNFHLRPamide (Antho-RPamide-2), pQVKLYRPamide (Antho-RPamide-3), GPHypSLFRPamide (Hyp = hydroxyproline; Antho-RPamide-4), and YRPamide (Antho-RPamide-5) [9–11, 52, 53, 63]. Using antisera against RPamide peptides, we found that these peptides were produced in ectodermal and endodermal sensory cells of the tentacles and oral disk from the sea anemone *Calliactis parasitica*, showing that these peptides were genuine neuropeptides [63]. This neuronal localization of Antho-RPamides was recently confirmed in *N. vectensis* [64]. Also, thirty years ago, we cloned a complete Antho-RPamide preprohormone from *A. elegantissima* [10] (Additional file 4), which contained one copy of Antho-RPamide-1 and one copy of an additional RPRPamide, while the other Antho-RPamides were lacking. This finding suggests the presence of at least 2 Antho-RPamide preprohormone genes (gene-1 and gene-2).

In the tested transcriptome from *A. elegantissima*, we identified an incomplete Antho-RPamide preprohormone that contained an Antho-RPamide-1 sequence and a second sequence, RPRPamide (Table 2, Additional file 4). This preprohormone fragment corresponds to the N-terminal part of our complete, cloned Antho-RPamide-1 preprohormone [10]. We also found another incomplete preprohormone fragment that contained two copies of Antho-RPamide-2, one copy of Antho-RWamide-3, two copies of Antho-RPamide-4, and several other RPamide fragments (Table 2, Additional file 4). In addition, we identified a third complete preprohormone, coding for three additional RPamide peptides. This preprohormone must be coded for by a third RPamide gene (Additional file 4). In *A. viridis*, we identified two incomplete preprohormone sequences that are probably fragments of the Antho-RPamide preprohormone, corresponding to the Antho-RPamide gene-2 product from *A. elegantissima.* These fragments contained the sequence of Antho-RPamide-4 together with 14 other, novel RPamide sequences, of which pQNLLRPamide was the most frequent one (Table 2, Additional file 4). In *N. vectensis*, a preprohormone fragment was identified, containing 9 RPamide peptides, among them pQLLFRPamide, and pQLFRPamide (Table 2, Additional file 4). In *P. crucifer*, a complete preprohormone was identified that contained 7 different RPamide peptides among them the sequence pQNNLLFRPamide (Table 2, Additional file 4). In *S. callimorphus*, an incomplete RPamide preprohormone was found, containing 8 different RPamide peptides (Table 2, Additional file 4). Finally, in *E. diaphana* we found a preprohormone with at least 10 copies of different RPamide peptides (Table 2, Additional file 4).

The fifth class of neuropeptides comprises peptides with the sequences pQITRFamide, pQVTRFamide, and NPPITRIamide, (Table 2, Additional file 5). Although they have the C-terminal sequence RFamide in common with the Antho-RFamides (neuropeptide family number 2 in Table 2), these two peptide families are probably not related, due to clear structural differences in the N-terminal halves of the peptides. Using our script, TRFamide/TRIamide preprohormones could not be identified in *A. elegantissima* (Table 2). Also, previously, members of this peptide family could not be purified and sequenced from extracts of *A. elegantissima*, although a radioimmunoassay for monitoring the purification of RFamide peptides was used [61]. These findings make the status of this peptide family somewhat uncertain.

The sixth class of peptides all have the C-terminal sequence FHIRamide or YHIRamide in common (Table 3, neuropeptide family #6). Their preprohormones contain four to ten copies of identical or slightly different peptide sequences (Additional file 6). These peptides are somewhat unusual, because they are quite long for a cnidarian neuropeptide (14 to 19 amino acid residues long, Additional file 6). Also, several members of this neuropeptide family have one or more acidic residues in the middle of their sequences. Most isolated and sequenced cnidarian neuropeptides are short sequences that lack acidic residues [9–13]. Therefore, we are somewhat in doubt about the neuropeptide status of these FHIRamide peptides.

### Preprohormones in Actiniaria containing a single neuropeptide copy

The seventh, eighth, and ninth class of neuropeptides (Table 3) comprise peptides with N-terminal phenyllactyl residues. Previously, using radioimmunoassays against RNamide or RVamide peptides, we isolated and sequenced three short neuropeptides from *A. elegantissima* that were N-terminally protected by L-3-phenyllactyl residues [56–58]. At that time, the biosynthesis of these N-terminal residues was unclear to us: It could be that the N-terminal L-3-phenyllactylresidue was derived from an N-terminal phenylalanyl residue, or that L-3-phenyllactyl was transferred to the N-terminus of the peptide by an acyltransferase [56]. Using Regex, but not our script [37], as a search tool, we identified the preprohormones for these neuropeptides in *A. elegantissima*. For the first neuropeptide, L-3-phenyllactyl-LRNamide (abbreviated phelacLRNamide), we found two different preprohormones, coded for by two different genes (Table 3, neuropeptide family #7). Each preprohormone had only one neuropeptide copy that was located directly after the signal sequence of the preprohormone (Additional file 7), thereby becoming the N-terminus of the prohormone after protein translocation through the rough endoplasmic reticulum (RER) membrane. The N-terminus of the immature neuropeptide copy was a phenylalanyl residue, thus confirming that L-3-phenyllactyl-LRNamide originated from L-3-phenylalanyl-LRNamide. In *A. viridis*, we found three different phelacLRNamide preprohormones; in *N. vectensis*, we found two; in *P. crucifer* we found two; in *S. callimorphus*, we found two; and also in *E. diaphana*, we found two preprohormones. These preprohormones were all single peptide copy preprohormones, where the immature neuropeptide sequences were located immediately after the signal sequence (Table 3, Additional file 7).

We have previously found that only neurons and no other cell types were stained with RNamide antibodies in the sea anemone *C. parasitica*, showing that the preprohormone is most likely expressed in neurons and that phelacLRNamide is probably a neuropeptide in all sea anemones listed in Table 3 [56].

Using a radioimmunoassay against RVamide peptides, we previously isolated L-3-phenyllactylYRIamide (abbreviated phelacYRIamide and named Antho-RIamide) from *A. elegantissima*, because this RIamide peptide crossreacted in our RVamide radioimmunoassay [58]. Using the software program Regex, we now found a preprohormone in *A. elegantissima* with a single copy of the immature neuropeptide sequence positioned directly after the signal sequence. Again, an N-terminal phenylalanyl residue is the substrate of the N-terminal L-3-phenyllactyl residue (Additional file 8; Table 3 neuropeptide family 8). *A. viridis* has a preprohormone, containing a single copy of Antho-RIamide located directly after the signal sequence (Table 3, Additional file 8). *N. vectensis* contains one preprohormone with a single copy of FYRVGKR positioned directly after the signal sequence (Additional file 8), thus likely yielding the mature peptide phelacYRVamide (Table 3); *P. crucifer* contains one preprohormone with a single copy of Antho-RIamide; *E. diaphana* contains one preprohormone with a single copy of FYKIamide, located directly after the signal sequence (Additional file 8), thus yielding the related peptide phelacYKIamide (Table 3). We could not identify a phelacYRIamide or related peptide preprohormone in *S. callimorphus* (Table 3, Additional file 8).

By using immunocytochemistry and RVamide antisera, we previously found that the Antho-RIamide preprohormone was located in neurons of the sea anemone *C. parasitica* and that Antho-RIamide, because of its crossreaction with RVamide antisera, therefore, was likely to be a neuropeptide in all sea anemones [58].

We previously isolated L-3-phenyllactylFKAamide (abbreviated phelacFKAamide and dubbed Antho-KAamide) from *A. elegantissima* [57]. Using Regex, we could identify a preprohormone in *A. elegantissima* that contained one copy of an immature Antho-KAmide sequence that was located immediately after the signal sequence (Table 3, Additional file 9). Also here, the N-terminal L-3-phenyllactyl residue was derived from an N-terminal phenylalanyl residue (Additional file 9). We also identified an Antho-KAamide preprohormone in *A. viridis*, *N. vectensis* and *E. diaphana* that had the same overall organization as the Antho-KAamide preprohormone from *A. elegantissima* (Table 3, Additional file 9). We were not able to identify Antho-KAamide preprohormones in *P. crucifer* or *S. callimorphus*.

The tenth class of peptides consists of short neuropeptides with the C-terminal sequence LRWamide (Table 3). Previously, we published the isolation and sequencing of two peptides from the sea anemone *A. elegantissima*, pQSLRWamide (Antho-RWamide-1) and pQGLRWamide (Antho-RWamide-2) [54, 55]. Using immunocytochemistry with an antiserum against the sequence RWamide, we found that these peptides were located in endodermal sensory neurons from the sea anemone *C. parasitica* that projected to the sphincter muscle located below the oral disk region of the animal, which was heavily innervated by these RWamide-positive processes [9]. This population of RWamide-positive sensory-motor-neurons was different from the Antho-RFamide-positive sensory neurons in the endoderm of that region, which did not project to the sphincter [9]. Using our script, we could not detect genes coding for LRWamide or SRWamide peptides in *A. elegantissima*, but using Regex, we found four genes, each coding for a complete, single copy-type of LRWamide preprohormone (Table 3, Additional file 10). The first preprohormone contains a single copy of pQQQGLRWamide; the second preprohormone contains a single Antho-RWamide-2 sequence; the third preprohormone has a single copy of Antho-RWamide-1; and the fourth preprohormone has a single copy of pQGGLRWamide. All four neuropeptide sequences are located immediately after the signal sequence of the preprohormone (Table 3, Additional file 10).

In *A. viridis*, there are three single-copy preprohormones, each containing a single LRWamide neuropeptide sequence located directly after the signal sequence (Table 3, Additional file 10). One of these sequences is identical to Antho-RWamide-1 (pQSLRWamide), while another one is one amino residue longer than Antho-RWamide-2 (pQGALRWamide) (Table 3).

In *N. vectensis*, we identified four single-copy preprohormones containing GLRWamide neuropeptides. These neuropeptide sequences were, again, located directly after the signal sequence of the preprohormone (Additional file 10). There are two different preprohormones and, therefore, two different genes, encoding Antho-RWamide-2 (pQGLRWamide); one preprohormone containing a GPPQGLRWamide sequence; and one containing a pQGGLRWamide sequence (Additional file 10, Table 3).

In *P. crucifer*, we identified two genes: One coding for a single copy preprohormone containing the sequence pQKPPGLRWamide and another gene, coding for a single copy preprohormone containing the sequence pQGIRWamide (Table 3, Additional file 10).

In *S. callimorphus*, we found a similar situation as in *N. vectensis* with two different genes, encoding two different preprohomones that each contained a single copy of Antho-RWamide-2. Furthermore, there were two preprohormones with N-terminally elongated LRWamide sequences (Table 3, Additional file 10).

In *E. diaphana*, we identified one preprohormone, containing one Antho-RWamide-2 sequence and another preprohormone, having an N-terminally elongated LRWamide sequence (Table 3, Additional file 10).

### Preprohormones in Scleractinia containing multiple neuropeptide copies

Using our script [37], we could identify seven neuropeptide families in Scleractinia derived from more than seven preprohormones. Five of these preprohormones contained multiple neuropeptide copies (Table 4, Table 5).

**Table 4:**
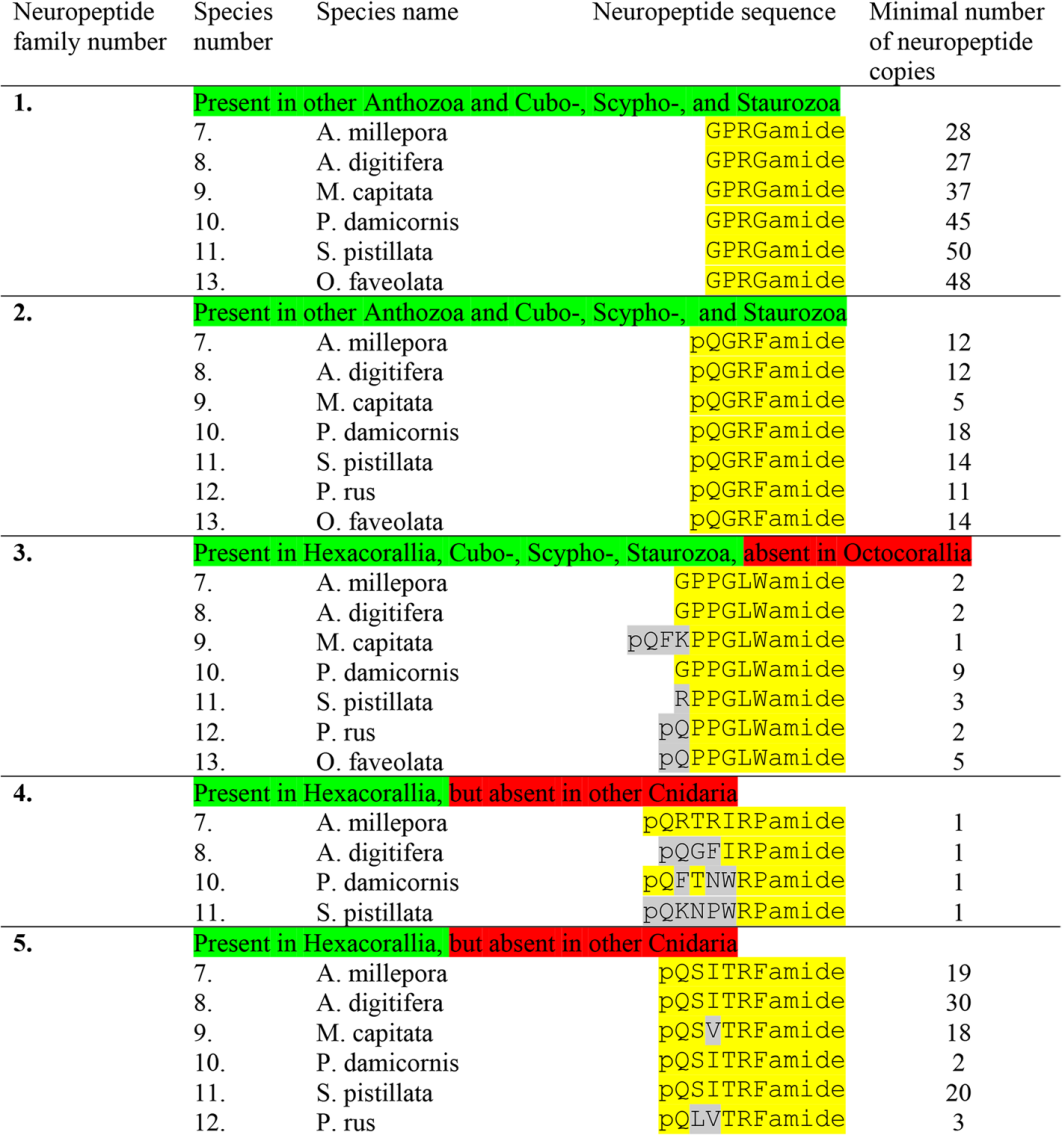
Neuropeptide families identified in seven species of Scleractinia: *Acropora millepora*, *Acropora digitifera*, *Montipora capitata*, *Pocillopora damicornis*, *Stylophora pistillata*, *Porites rus*, and *Orbicella faveolata*. These neuropeptide families have preprohormones that contain multiple identical or similar neuropeptide copies. Only those neuropeptides with the highest copy numbers are given. The preprohormone amino acid sequences are shown in Additional files 1 to Additional files 5

**Table 5:**
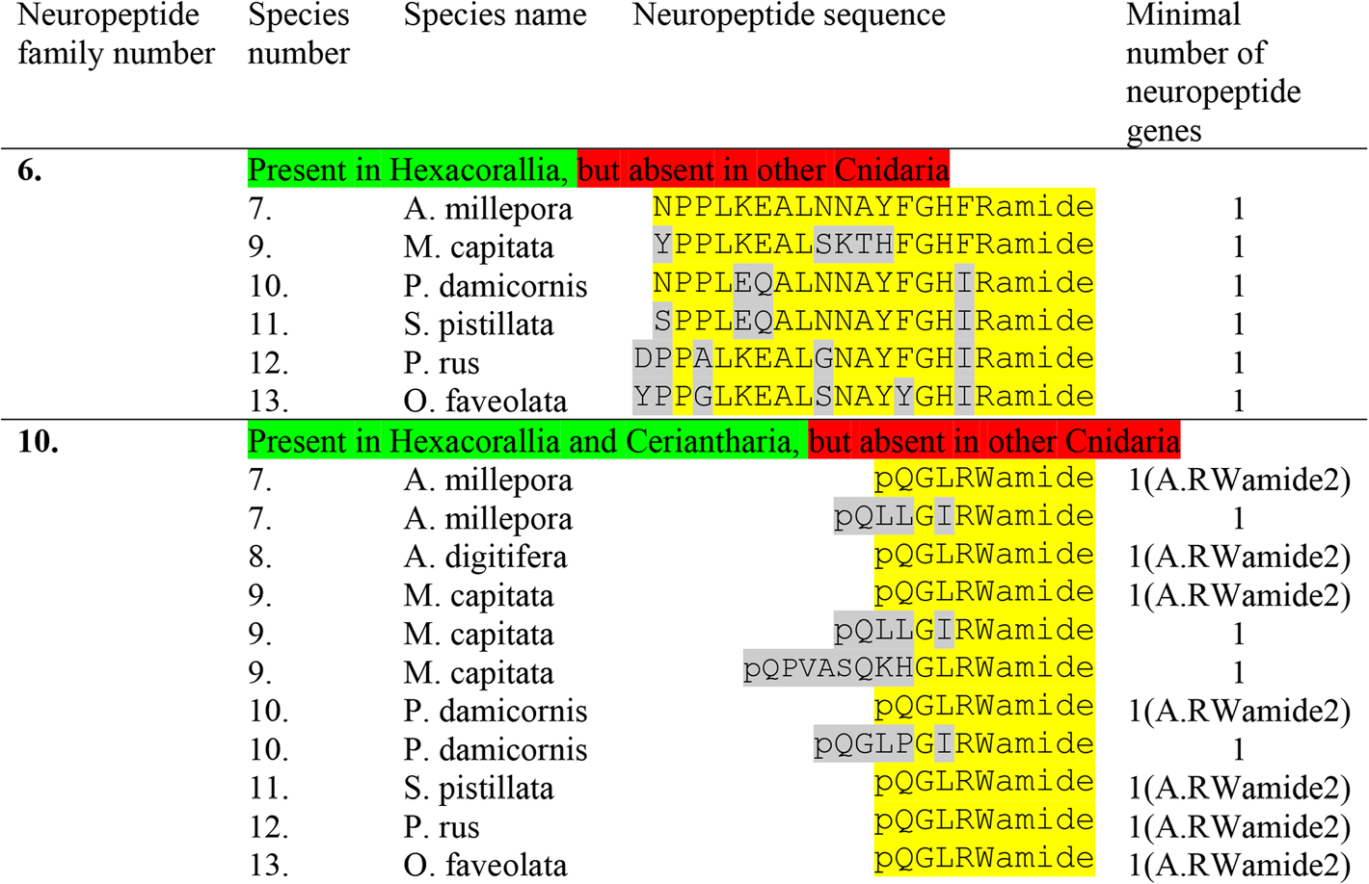
Presence of neuropeptide families #6 and #10 in seven species of Scleractinia: *A. millepora*, *A. digitifera*, *M. capitata*, *P. damicornis*, *S. pistillata*, *P. rus*, and *O. faveolata*. This table is a continuation of Table 4. Neuropeptide family #6 is derived from preprohormones with multiple neuropeptide copies, while neuropeptide family #10 is derived from single neuropeptide copy preprohormones. The preprohormone amino acid sequences are given in Additional file 6 and Additional file 10. A.RWamide2 = Antho-RWamide-2

In the stony coral *Acropora millepora*, we could identify a preprohormone fragment that contained 28 copies of the neuropeptide GPRGamide (Table 4, neuropeptide family number 1; Additional file 1, heading Scleractinia). In *Acropora digitifera*, we found a preprohormone fragment that contained 27 copies of GPRGamide; in *Montipora capitata* a preprohormone fragment with 37 copies of GPRGamide; in *Pocillopora damicornis* a preprohormone fragment with 45 copies of GPRGamide; in *Stylophora pistillata* a complete preprohormone with the impressive number of 50 copies of GPRGamide; no GPRGamide preprohormone in the transcriptome of *Porites rus*; but in *Orbicella faveolata* a preprohormone fragment with 48 copies of GPRGamide (Table 4; Additional file 1).

The second neuropeptide family comprises the Antho-RFamides (pQGRFamide). In *A. millepora,* we found an incomplete Antho-RFamide preprohormone containing 12 copies of Antho-RFamide; in *A. digitifera* a complete preprohormone containing 12 copies of Antho-RFamide; in *M. capatata* a preprohormone fragment containing 5 copies of Antho-RFamide; in *P. damicornis* a preprohormone fragment containing 18 copies of Antho-RFamide; in *S.pistillata* a complete preprohormone containing 14 copies of Antho-RFamide; in *P. rus* a complete preprohormone containing 11 copies of Antho-RFamide; and in *O. faveolata* a preprohomone fragment containing 14 copies of Antho-RFamide (Table 4; Additional file 2).

Most members from the third neuropeptide family consist of peptides having the PPGLWamide C-terminus (Table 4; Additional file 3). In *A. millepora* we found a preprohormone fragment containing 2 copies of GPPGLWamide and 3 other GLWamide peptides; in *A. digitifera* a preprohormone fragment with 2 GPPGLWamide copies and 3 other GLWamide peptides; in *M. capitata* a fragment with 1 copy of pQFKPPGLWamide and 4 other GLWamide peptides; in *P. damicornis* a fragment with 9 copies of GPPGLWamide; in *S. pistillata* a complete preprohormone with 3 copies of RPPGLWamide and 5 copies of other GLWamide peptides; in *P. rus* a complete preprohormone with 2 copies of pQPPGLWamide and 4 copies of other GLWamide peptides; and in *O. faveolata* a fragment with 5 copies of pQPPGLWamide and 1 other GLWamide peptide (Table 4; Additional file 3).

We also found the fourth neuropeptide family, containing the RPamide C-terminus, in Scleractinia. In *A. millepora*, we identified an incomplete preprohormone, containing 1 copy of pQRTRIRPamide and 2 other RPamide peptides; in *A. digitifera* an incomplete preprohormone, containing 1 copy of pQGFIRPamide and 2 other RPamide peptides; in *P. damicornis* a preprohormone fragment, containing one copy of pQFTNWRPamide and two other RPamide peptides; and in *S. pistillata* a complete preprohormone, containing 1 copy of pQKNPWRPamide and 3 copies of other RPamide peptides (Table 4; Additional file 4). In the other tested scleractinians, we were not able to detect RPamide preprohormones.

The fifth peptide family consists of pQSITRFamide peptides or closely related peptide sequences (Table 4, neuropeptide family 5). These peptides resemble neuropeptide family 5 from Table 2, but are elongated in their N-terminal halves by one amino acid residue. We identified a complete preprohormone in *A. millepora* with 19 copies of pQSITRFamide; in *A. digitifera* a complete preprohormone with 30 copies of the same peptide; in *M. capitata* a complete preprohormone with 18 copies of pQSVTRFamide; in *P. damicornis* an incomplete preprohormone with two copies of pQSITRFamide and one copy of pQSVTRFamide; in *S. pistillata* a complete preprohormone with 20 copies of pQSITRFamide; and in *P. rus* a complete preprohormone with 3 copies of pQLVTRFamide (Table 4, Additional file 5).

Also members of the sixth peptide family, the FHIRamides, are present in Scleractinia. We could identify them in *A. millipora*, *M. capitata*, *P. damicornis*, *S. pistillata*, *P. rus*, and *O. faveolata* (Table 5, neuropeptide family #6; Additional file 6).

### Preprohormones in Scleractinia containing a single neuropeptide copy

We were unable to find phenyllactyl peptides in Scleractinia, corresponding to the ones identified in Actiniaria (Table 3, neuropeptide families 7, 8, and 9). However, we found preprohormones containing Antho-RWamide-2 (pQGLRWamide) and related peptides (Table 5, neuropeptide family #10). In all cases, these preprohormones only contained one single neuropeptide copy, which was situated directly after the signal sequence, just as we saw earlier in Actiniaria (Additional file 10).

In *A. millepora*, we identified two complete preprohormones, one containing a single copy of Antho-RWamide-2, and the other one, containing a single copy of pQLLGIRWamide. In *A. digitifera*, we identified a complete preprohormone, containing a single copy of Antho-RWamide-2. In *M. capitata*, we identified three complete preprohormones, one containing a single copy of Antho-RWamide-2, one containing the peptide pQLLGIRWamide, and one containing the peptide pQPVASQKHGLRWamide. In *P. damicornis*, we found a preprohormone, containing one copy of Antho-RWamide-2 and another preprohormone, containing the sequence pQGLPGIRWamide. In *P. rus*, we identified a preprohormone with one copy of Antho-RWamide-2. The same was found in *O. faveolata* (Table 5; Additional file 10).

### Preprohormones in Corallimorpharia, Zoantharia, and Ceriantharia containing multiple neuropeptide copies

In the following, we are investigating the presence of preprohormones with multiple neuropeptide copies in four species, belonging to the order Corallimorpharia (*Amplexidiscus fenestrafer*, *Corynactis australis*, *Discosoma* sp. and *Ricordea yuma*); in two species, belonging to the order Zoantharia (*Protopalythoa variabilis* and *Zoanthus* sp.); and in one species, belonging to the subclass Ceriantharia (*Pachycerianthus borealis*).

In all seven species we identified preprohormone fragments with multiple copies of GPRGamide (neuropeptide family #1, Table 6): In *A. fenestrafer* there are 42 copies; in *C. australis* 21 copies; in *Discosoma* sp. 46 copies; in *R. yuma* 45 copies; in *P. variabilis* 17 copies; in *Zoanthus* sp. 19 copies; and in *P. borealis* 8 copies (Additional file 1).

**Table 6:**
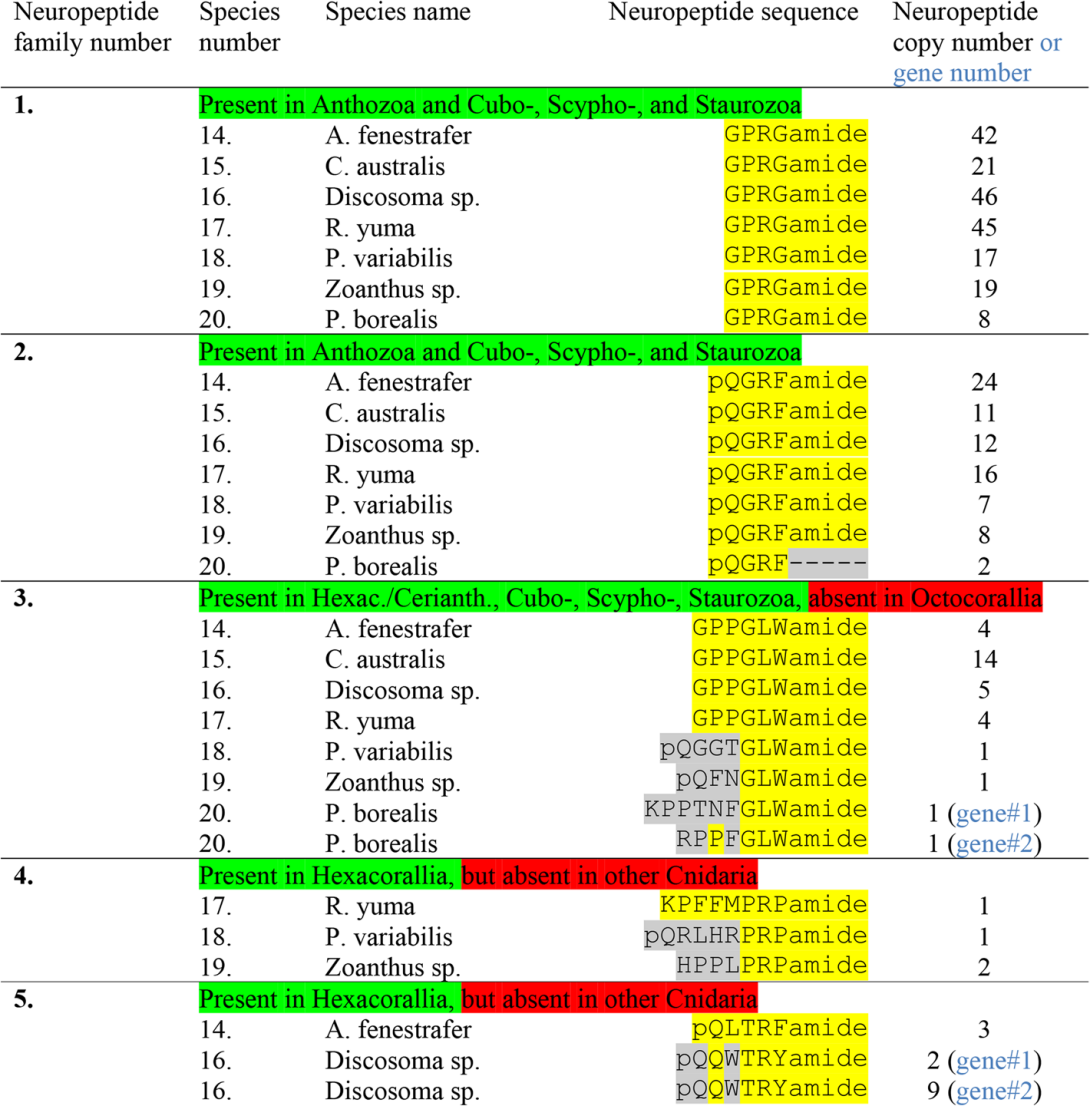
Neuropeptide families identified in four species of the order Corallimorpharia: Amplexidiscus fenestrafer, Corynactis australis, Discosoma sp., and Ricordea yuma; two species of the order Zoantharia: Protopalythoa variabilis and Zoanthus sp.; and one species of the Ceriantharia subclass: Pachycerianthus borealis. Neuropeptide families # 1-5 always have multiple neuropeptide copies on their preprohomones. Sometimes there are two genes, which is indicated (highlighted in blue) in the last column. The preprohormone amino acid sequences are given in Additional file 1 to Additional file 5

Similarly, we found preprohormones with multiple copies of Antho-RFamide (neuropeptide family #2, Table 6) in all seven species: In *A. fenestrafer*, there are 24 copies of Antho-RFamide; in *C. australis* 11 copies; in *Discosoma* sp. 12 copies; in *R. yuma* 16 copies; in *P. variabilis* 7 copies; and in *Zoanthus* sp. 8 copies.

We encountered a short, unusual Antho-RFamide preprohormone fragment in the ceriantharian *P. borealis* (Table 6; Additional file 2). Here we identified 2 copies of Antho-RFamide (out of 2), that were not C-terminally amidated. The mature copies that would be released would thus be pQGRF (Table 6) and, therefore, would not correspond to genuine Antho-RFamide.

The third neuropeptide family of GLWamides (Table 6, Additional file 3) is present in all mentioned species: In *A. fenestrafer*, we identified a complete preprohormone, containing 4 copies of GPPGLWamide and 4 other GLWamide peptides; in *C. australis*, two non-overlapping preprohormone fragments, each containing 7 copies of GPPGLWamide; in *Discosoma* sp. a fragment with 5 copies of GPPGLWamide; in *R. yuma* a preprohormone, containing 4 copies of GPPGLWamide and 3 other GLWamide peptides; in *P. variabilis* a preprohormone, containing one copy of pQGGTGLWamide and 6 other GLWamides; in *Zoanthus* sp. a preprohormone, containing one copy of pQFNGLWamide and 3 other GLWamides; and in *P. borealis* two separate genes, one coding for a copy of KPPTNFGLWamide and another GLWamide peptide and one coding for a copy of RPPFGLWamide and an additional GLWamide (Table 6; Additional file 3).

The fourth neuropeptide family, comprising the Antho-RPamides (Table 6), could be identified in all mentioned species, except for *A. fenestrafer*, *C. australis*, *Discosoma* sp., and *P. borealis*: In *R. yuma* we found a preprohormone, containing 1 copy of KPFFMPRPamide and another RPamide peptide; in *P. variabilis* a preprohormone, containing 1 copy of pQRLHRPRPamide together with 2 other RPamide peptides; and in *Zoanthus* sp. a preprohormone containing 2 copies of HPPLPRPamide together with another resembling RPamide peptide (Table 6; Additional file 4).

The fifth peptide family (Table 6) could only be identified in two corallimorpharian species, *A. fenestrafer* and *Discosoma* sp. In *A. fenestrafer* we identified a preprohormone, containing 3 copies of pQLTRFamide. In *Discosoma* sp., we identified two genes (or 2 cDNAs): One gene coding for 2 copies of pQQWTRYamide and one gene coding for 9 copies of pQQWTRYamide (Additional file 5).

The sixth peptide family, the FHIRamides, could be identified in all four Corallimorpharia species: *A. fenestrafer*, *C. australis*, *Discosoma* sp., and *R. yuma*; and in one Zoantharia species: *Zoanthus* sp. (Table 7, neuropeptide family #6; Additional file 6). We were unable to find FHIRamide peptides in Ceriantharia.

**Table 7:**
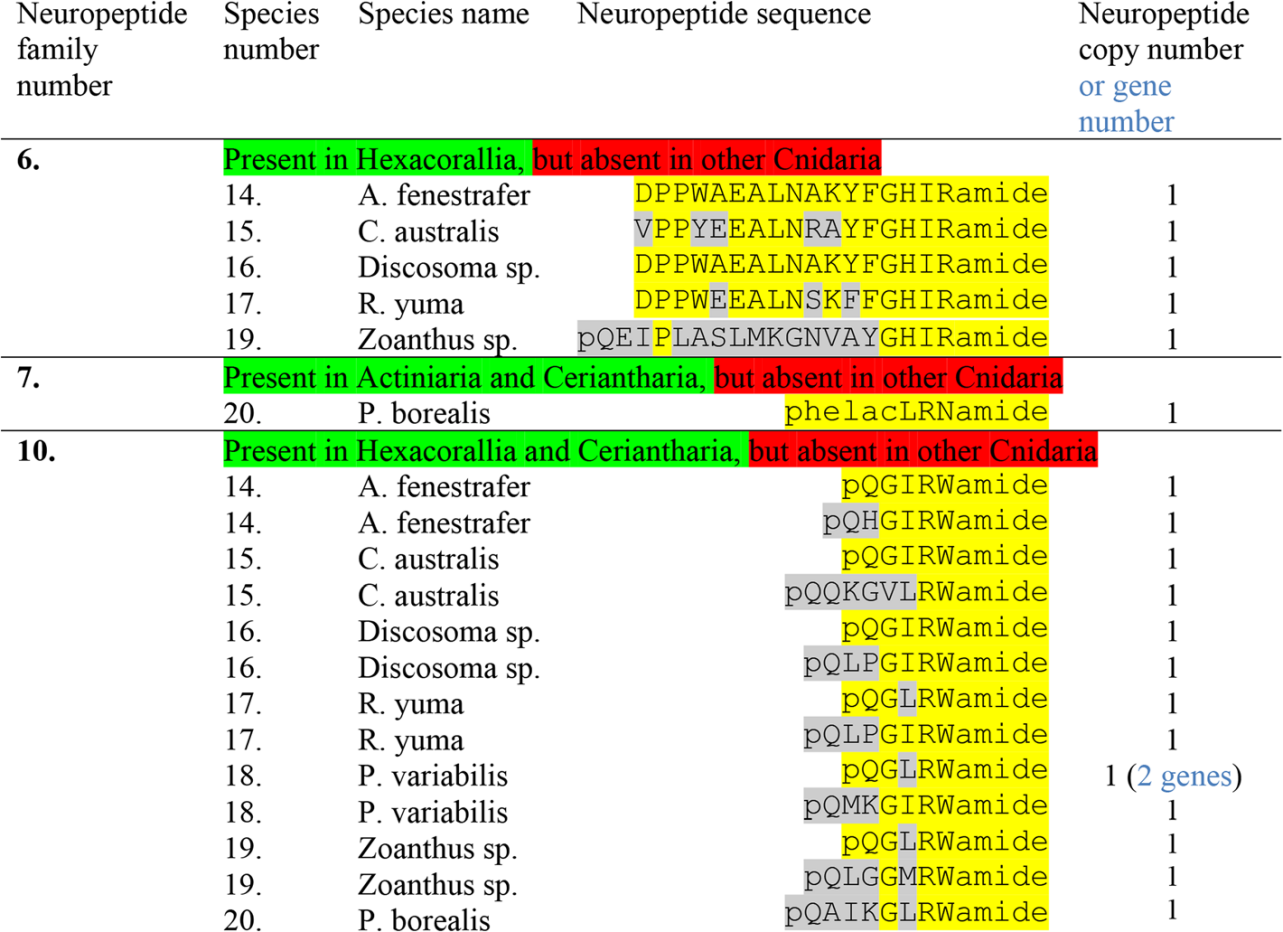
Presence of neuropeptide families in four species of the order Corallimorpharia: A. fenestrafer, C. australis, Discosoma sp., and R. yuma; two species of the order Zoantharia: P. variabilis and Zoanthus sp.; and one species of the Ceriantharia subclass: P. borealis. This table is a continuation of Table 6. Peptide family #6 is derived from preprohormones containing several similar neuropeptide copies. Peptide families #7 and #10 are derived from single copy preprohormones. Sometimes there are two genes, which is indicated in the last column (highlighted in blue). The amino acid sequences of the preprohormones are given in Additional file 6, Additional file 7, and Additional file 10

### Preprohormones in Corallimorpharia, Zoantharia, and Ceriantharia containing a single neuropeptide copy

We were unable to find phenyllactyl peptides in Corallimorpharia and Zoantharia species, corresponding to the ones found in Actiniaria (Table 3, neuropeptide families 7, 8, and 9). Surprisingly, however, we could identify an Antho-RNamide preprohormone in the ceriantharian *P. borealis* (Table 7, neuropeptide family 7; Additional file 7).

In all seven species, belonging to the Corallimorphia, Zoantharia, or Ceriantharia, we found preprohormones, containing a single copy of an RWamide peptide that was located directly after the signal sequence (Table 7, neuropeptide family number 10): In *A. fenestrafer* we identified a preprohormone, containing a single copy of pQGIRWamide and another preprohormone, containing a single copy of pQHGLRWamide; in *C. australis* a preprohormone, containing a single copy of pQGIRWamide and another preprohomone, containing a single copy of pQQKGVLRWamide; in *Discosoma* sp. one preprohormone, containing a single copy of pQGIRWamide and a second preprohormone, containing a single copy of pQLPGIRWamide; in *R. yuma* a preprohormone, containing 1 copy of Antho-RWamide-2 (pQGLRWamide) and another preprohormone, containing a single copy of pQLPGIRWamide; in *P. variabilis* 2 preprohormones (encoded by 2 genes), each containing a single copy of Antho-RWamide-2, and another preprohormone, containing a single copy of pQMKGIRWamide; in *Zoanthus* sp. a preprohormone, containing a single copy of Antho-RWamide-2, and another preprohormone, containing a single copy of pQLGGMRWamide; and in *P. borealis* a preprohormone, containing a single copy of pQAIKGLRWamide (Additional file 10).

## Discussion

**Fig. 3:**
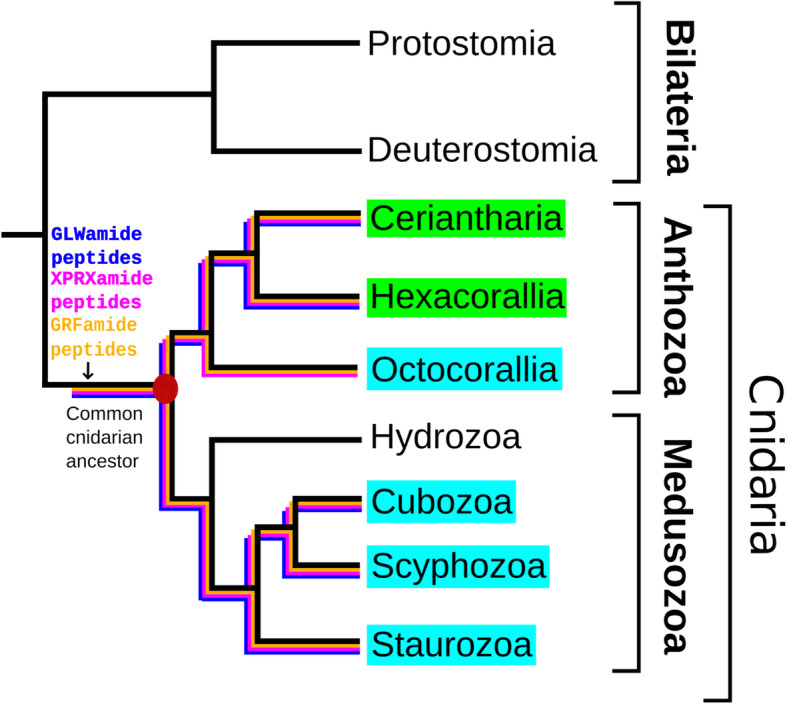
Presence of the GLWamide (highlighted in blue), GRFamide (highlighted in yellow), and X_1_PRX_2_amide (where X_2_ is either S, A, or G; highlighted in purple) peptides in the subclasses Ceriantharia, Hexacorallia, and Octocorallia and the classes Cubozoa, Scyphozoa, and Staurozoa. This figure has the same structure as Fig.1. It shows that the three peptide families are present in all mentioned classes and subclasses, except for the Octocorallia that lack the GLWamides. Nevertheless, it can be concluded that the three peptide families originated in the common cnidarian ancestor. The other neuropeptide families identified in this paper (Additional files 4, 5, 6, 7, 8, 9, and 10) are either class-, subclass-, or order-specific and are no good candidate for being primordial neuropeptides

In a previous paper [34], we have found that two cnidarian neuropeptide families, the GRFamides and X_1_PRX_2_amides, are occurring in all investigated species belonging to the cnidarian classes Cubozoa, Scyphozoa, Staurozoa, and Octocorallia. Based on the phylogenetic positions of these four cnidarian classes, we were able to conclude that the GRFamide and X_1_PRX_2_amide peptides originated in the common cnidarian ancestor (Fig. 1) [34]. The data from our current analyses, which show that the GRFamide and X_1_PRX_2_amide peptides (neuropeptide families #1 and #2 from Tables 2, 4, and 6; Additional files 1 and 2) are present in all investigated Hexacorallia and Ceriantharia species, strongly support this conclusion (Fig. 3).

For the third class of neuropeptides, the GLWamides (neuropeptide family #3 from Tables 2, 4, and 6; Additional file 3) we have previously found that they were present in Cubo-, Scypho-, and Staurozoa, but absent in Octocorallia [34]. It appeared, therefore, that the GLWamide family was limited to a part of the cnidarian subphylum Medusozoa (Fig. 1). In our present study, however, we find GLWamide peptides in all investigated hexacorallian and ceriantharian species (Tables 2, 4, 6; Additional file 3). Because Ceriantharia, Hexacorallia, Cubo-, Scypho-, and Staurozoa root in their common cnidarian ancestor (Fig. 1), we can now conclude that also the GLWamide peptides must have originated in the common cnidarian ancestor (Fig. 3) and that these peptides probably evolved together with the functional and molecular expansion of its nervous system.

The absence of GLWamide peptides in Octocorallia [34] suggests that that this order has lost the genes coding for the GLWamides. Gene losses have, so far, not been observed, when studying the evolution of cnidarian neuropeptides [34], but in other invertebrate groups, such as insects, neuropeptide gene loss is common [65].

**Fig. 4:**
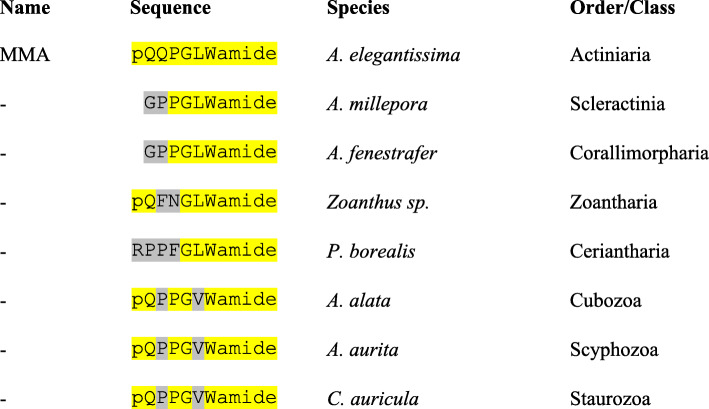
Alignment of GLWamide and GVWamide peptides from species belonging to the subclasses Hexacorallia and Ceriantharia, or orders Cubozoa, Scyphozoa, and Staurozoa. These alignments show that the GVWamides have four out of six residues in common with the canonical GLWamide peptide MMA. In addition, there are conserved L/V residues at position 5 of all peptides

When we aligned the amino acid sequences of the GLWamide peptides from Cubo-, Scypho-, and Staurozoa with those from the Hexacorallia and Ceriantharia orders, we find many identical or highly similar residues, despite the fact that the Cubo-, Scypho-, and Staurozoa sequences do not have the GLWamide, but the GVWamide C-terminal sequence (Fig. 4). For example, the Metamorphosin A (MMA) sequence from *A. elegantissima*, pQQPGLWamide [21], is highly similar to the conserved pQPPGVWamide sequence from cubo-, scypho-, and staurozoan species (Fig. 4). These findings suggest that the family of pQPPGVWamide peptides that we annotated from cubo-, scypho-, and staurozoan species [34], might also be involved in planula metamorphosis, just as MMA is in hexacorals [21, 62].

The fourth family of neuropeptides (neuropeptide family #4 in Tables 2, 4, and 6; Additional file 4) all have the C-terminal sequence RPamide in common, but their N-termini are quite variable, although there is an overrepresentation of P, R, and aromatic and aliphatic residues. We have previously isolated and sequenced four Antho-RPamides from the sea anemone *A. elegantissima* [9–11, 52, 53, 63] and cloned the preprohormone for one of them, namely Antho-RPamide-1 (LPPGPLPRPamide) [10]. In our current paper, we have identified two additional Antho-RPamide preprohormones from *A. elegantissima*, of which one (Table 2, neuropeptide family #4, gene #2; Additional file 4) represents the complete preprohormone for the isolated neuropeptides Antho-RPamide-2, − 3, and − 4.

Very recently, another research group published the cloning of a preprohormone for two potential *N. vectensis* RPamide peptides, LPKPRPamide and FPPGFHRPamide [64]. This preprohormone and its RPamide neuropeptides, however, are different from the preprohormone and its RPamide peptides that we identified in our current paper in *N. vectensis* (Table 2, neuropeptide family #4; Additional file 4). These findings implicate that *N. vectensis* has at least two genes, each coding for different RPamide preprohormones, which is a situation resembling that in *A. elegantissima* (Table 2, neuropeptide family #4).

We discovered RPamide preprohormones in all six investigated Actiniaria species, in four (out of seven) Scleractinia species, in one (out of four) Corallimorpharia species, and in two (out of two) Zoantharia species, showing that RPamide peptides are widespread in Hexacorallia (Tables 2, 4, 6, neuropeptide family #4). The fact that we could not identify RPamide peptides in all tested hexacorals was probably due to the varying qualities of their transcriptomes. We were unable to find RPamides in the classes Ceriantharia, Octocorallia, Cubo-, Scypho-, and Staurozoa (Table 6, neuropeptide family #4) [34]. These negative results were probably not due to low-quality databases, because several of the investigated species had high-quality transcriptomes [37, 49]. The conclusion is, therefore, that the RPamide peptides are probably confined to the Hexacorallia.

Using RPamide antibodies, we established previously that Antho-RPamides were produced by ecto- and endodermal sensory neurons of the tentacles and oral disk of the sea anemone *C. parasitica* [63] (Additional file 11). Also, another research group recently localized RPamide peptides in a sensory ectodermal nerve net at the aboral pole of *N. vectensis* planula larvae [64]. Therefore, it can be assumed that RPamides are neuropeptides in all hexacorals.

The fifth peptide family, the pQITRFamides (Tables 2, 4, and 6, peptide family #5) is also Hexacorallia-specific. It occurs as pQITRFamide in Actiniaria (Table 2, peptide family #5), mainly as pQSITRFamide in Scleractinia (Table 4, peptide family #5) and as pQLTRFamide or pQQWTRYamide in Corallimorpharia (Table 6, neuropeptide family #5). Although the pQITRFamide preprohormones have all the characteristics of neuropeptide preprohormones (Additional file 5), we are uncertain about their status, mainly because their peptide products, in contrast to all the neuropeptides mentioned above, have never been isolated. Yet, the TRFamides could possibly represent a neuropeptide family.

The sixth peptide family has a similar uncertain status as the pQITRFamides. These peptides are characterized by having an FHIRamide or FGHIRamide C-terminus (Tables 3, 5, and 7, peptide family #6). Although their preprohormones show all the characteristics of a cnidarian neuropeptide preprohormone (Additional file 6), the deduced mature neuropeptides have unusual properties for a cnidarian neuropeptide. Most established cnidarian neuropeptides are small (around six amino acid residues long) and lack acidic residues, possibly because acidic residues can be processing sites for prohormone endoproteinases [9–13, 64]. The FHIRamide peptide sequences that we derived from the FHIRamide preprohormones, however, were unusually long, ranging from 14 to 19 amino acid residues. Moreover, they often contained one or more acidic residues in the middle of their proposed amino acid sequences (Tables 3, 5, 7, neuropeptide family #7; Additional file 7), which is unusual for cnidarian neuropeptides. It is correct that a peptide with the proposed sequence QPPYLDLTPSYFHIRamide and additional peptides with related sequences were recently isolated from the sea anemone *N. vectensis* and sequenced using mass spectrometry (Fig. 2 from ref. [36]). Yet, we are not convinced that these sequences represent genuine neuropeptides.

The seventh (Antho-RNamide), eighth (Antho-RIamide), and ninth (Antho-KAamide) peptide families all have the unusual N-terminal L-3-phenyllactyl residues and their occurrence is confined to the Actinaria (Table 3, neuropeptide family # 7, 8, 9) and Ceriantharia (Table 7, neuropeptide family #7). These peptides have been previously isolated from *A. elegantissima* and sequenced using mass spectrometry [56–58]. Furthermore, varying biological actions on different sea anemone muscles and isolated muscle cells have been measured for these peptides [9, 10, 58, 66, 67] and for Antho-RIamide its neuronal localization has been determined [58]. It is, therefore, likely that these peptides are also functional neuropeptides in the other actiniarians and ceriantharians.

The identification of the phenyllactyl peptide preprohormones showed that the N-terminal phenyllactyl group originates from an N-terminal phenylalanyl residue that is located directly after the signal sequence (Additional files 7, 8 and 9). The nature of the enzymes that catalyze the posttranslational conversion of N-terminal phenylalanyl into N-terminal phenyllactyl residues remains unclear. These enzymes are probably present in the lumen of the rough endoplasmic reticulum (RER) and act after removal of the signal sequence during preprohormone translocation across the RER membrane.

The tenth neuropeptide family consists of peptides with the C-terminus RWamide, being most frequently LRWamide (Tables 3, 5, 7, neuropeptide family #10). Their preprohormones contain only one copy of the RWamide peptide that is located directly after the signal sequence (Additional file 10). We have previously isolated and sequenced two RWamide peptides from *A. elegantissima*, Antho-RWamide-1 (pQSLRWamide) and Antho-RWamide-2 (pQGLRWamide). Screening of the *A. elegantissima* transcriptome database, however, revealed four genes, coding for slightly different RWamide peptides. The same phenomenon was observed for the other actinarians (Table 3, neuropeptide family 10). The creation of multiple neuropeptide genes might be an effective way of producing large amounts of neuropeptides and be an alternative to single neuropeptide genes having multiple copy preprohormones.

RWamide peptides occur in all tested species belonging to the Actiniaria, Scleractinia, Corallimorpharia, Zoantharia, and Ceriantharia (Tables 3, 5, 6, neuropeptide family #6). Earlier, we have been unable to identify RWamide peptides in Octocorallia and in Cubo-, Scypho-, and Staurozoa [34]. A re-investigation of these cnidarian classes and subclass, however, has now shown that Octocorallia produce RWamide peptides, but that the Cubo-, Scypho-, and Staurozoa don’t (TL Koch, unpublished results). The RWamide peptides, therefore, appear to be confined to the Anthozoa (Fig. 1).

We have previously found that the RWamide peptides are expressed by a set of endodermal sensory motor neurons that project to the sphincter muscle of the sea anemone *C. parasitica*, showing that the RWamides are neuropeptides and suggesting a role in neuromuscular transmission [9, 10]. In agreement with these anatomical findings, we have observed that nanomolar concentrations of Antho-RWamide-1 or − 2 induced contractions in *C. parasitica* sphincter muscle preparations or isolated sphincter muscle cells, showing that the Antho-RWamides might be neuromuscular transmitters [68]. All these findings suggest that the RWamide peptides in the other hexacorals and ceriantharians also might play a role in neuromuscular transmission.

The phylogenetic position of the subclass Ceriantharia within the class Anthozoa has remained uncertain [59, 60, 69–71]. Based on the analyses of their mitochondrial genomes, one study concluded that the ceriantharians are a sister group to a clade containing the Octocorallia plus the Hexacorallia, while other data suggested them to be sister to the Hexacorallia [70, 71]. We realize, of course, that neuropeptide genes are only a very small fraction of all nuclear genes present in an animal. However, our data on the neuropeptide genes in the ceriantharian *P. borealis* strongly suggest that ceriantharians are more closely related to Hexacorallia than to Octocorallia. This is especially clear for the GLWamide genes that are absent in Octocorallia [32], but present in *P. borealis* and in all Hexacorallia (Table 6, Supplementary file 3). *P. borealis* even has two genes coding for GLWamide preprohormones and these proteins contain GLWamide neuropeptide sequences that are strongly resembling the GLWamide neuropeptides from hexacorals (Table 6, Fig. 4). These data argue for a phylogenetic position of Ceriantharia as depicted in Fig. 2 and Fig. 3.

In addition to increasing our knowledge of neuropeptide evolution in Cnidaria (Fig. 3), our present study could also contribute to obtaining a more realistic and complete view on the molecular neurobiology of hexacoral models such as *N. vectensis*, *E. diaphana*, and *A. millepora*. In the following paragraphs, we will illustrate this point for *N. vectensis*.

Shortly after the publication of the *N. vectensis* genome paper [72], Anctil analyzed the *N. vectensis* genomic sequence for the presence of receptors and neurohormones involved in neurotransmission and intercellular communication [73]. In addition to finding a large number of interesting and important proteins, he also postulated the occurrence of vertebrate neuropeptides in *N. vectensis*, such as gonadotropin-releasing hormone, galanin, and vasopressin [73]. In our study, however, we find no evidence for the presence of these neuropeptides in *N. vectensis* or in any other anthozoan species. This conclusion is in line with the findings of Plachetzki et al. [74], who concluded that gonadotropin-releasing hormone, galanin, and vasopressin and their receptors were absent in cnidarians. For assigning a peptide as vasopressin, one needs, in addition to the three identical C-terminal amino acid residues PRGamide found by Anctil [73], the characteristic cystine bridge ring structure of the peptide and other conserved residues [65, 75], which were not present in the postulated *N. vectensis* peptide [73]. In fact, from all the 30 postulated neuropeptide sequences in Table 4 from [73], we and others could only confirm 6 neuropeptide sequences. These sequences were (1) Antho-RFamide (pQGRFamide), which we also found in *N. vectensis* (Table 2, neuropeptide family #2); (2) pQAGAPGLWamide (Table 2, neuropeptide family 3); (3) (G)APGLWamide – this peptide has slightly been misinterpreted by Anctil [73] as is indicated by the parentheses around the residue that should probably be omitted (Table 2, neuropeptide family 3); and (4) pQITRFamide (Table 2, neuropeptide family 5). In addition, we annotated a preprohormone in *N. vectensis* that contained ten RPamide peptides (Table 2, neuropeptide family #4; Additional file 4). However, Anctil [73] annotated four RPamide peptides, which were different from the ones that were identified by us. The expression of two of them could be confirmed by Zang and Nakanishi by molecular cloning of their common preprohormone [64]. These two sequences were originally annotated by Anctil as (pQDAF)LPKPRPamide, and (pQDSSNYE)FPPGFHRPamide [73]. However, the latter sequence was partly misinterpreted by Anctil [73] (the sequence in between the parentheses should be omitted) and was later corrected by Zang and Nakanishi [64], and Hayakawa et al. [36]. Similarly, also the first sequence might be shorter, due to a potential endoproteolytic cleavage site at D [64]. Thus, *N. vectensis* likely expresses two RPamide preprohormone genes, one identified by us, producing ten (Additional file 4) and the other identified by Anctil [73], producing two RPamide neuropeptides.

In a recent paper [36], Hayakawa et al. analyzed the neuropeptide content of *N. vectensis*, using a similar bioinformatics approach as we published earlier [37] in combination with mass spectrometry. Their findings [36], however, were somewhat different from the ones that we have summarized in Table 2 and Table 3. These differences are shortly listed here: (1) Hayakawa et al. [36] concluded that Antho-RFamide (Table 2, Neuropeptide family #2) occurred as a kind of dimeric peptide with the sequence **pQGRFG**RED**QGRFamide** (the two single peptide sequences are in bold). It is hard for us to agree with this conclusion, because we have isolated, using RFamide radioimmunoassays, large quantities of genuine Antho-RFamide (pQGRFamide) from both the sea anemone *A. elegantissima* [61] and the octocoral *Renilla köllikeri* [76], without finding measurable amounts of pQGRFGREDQGRFamide. Furthermore, the dimeric peptide sequence contains several established endoproteolytic processing sites for cnidarian preprohormones, such as the RED sequence that contains three such sites [9–11], including the basic cleavage site for prohormone convertase [77, 78]. Also, it would not be possible to create the dimeric sequence, which contains an N-terminal pQ group, without N-terminal cleavage at E or D residues in the prohormone (Additional file 2) [36]. We propose, therefore, that the dimeric peptide sequence might be an Antho-RFamide prohormone processing intermediate. (2) Similarly, a peptide named PRGamide was found with the sequence **GPRGG**RATEF**GPRGamide** [36]. Again, this sequence is a dimeric form of two peptides that we annotated as GPRGamides (Table 1, neuropeptide family #1). Also in this case, the dimeric peptide has several cleavage sites in the middle of its sequence, such as RATEF that has at least two of these sites, among them a site for prohormone convertase [77, 78]. Furthermore, it is not possible to release the dimeric peptide sequence from its prohormone without cleavage at E and R residues (Additional file 1) [36]. Therefore, we propose that GPRPamide (and APRPamide) are the final peptide products from the *N. vectensis* preprohormone given in Additional file 1, and that the dimeric form found in [36] is a processing intermediate. (3) A neuropeptide preprohormone, dubbed QWamide precursor, was proposed that lacked a signal sequence [36]. According to current knowledge of basal cell biology, however, this precursor cannot be part of the secretory pathway and, thus, not be a preprohormone for neuropeptides. (4) A neuropeptide preprohormone, named HIRamide precursor, was identified [36], corresponding to the FHIRamide preprohormone in our study (Additional file 6; Table 3, neuropeptide family #6). The authors proposed that the FHIRamide peptides produced by this preprohormone are evolutionarily related to the vertebrate tachykinins [79], indicating a deep evolutionary origin of these bilatarian neuropeptides [36]. We find, however, that the FHIRamide peptides only occur in Hexacorallia, which would not support their presence in the common cnidarian ancestor (Fig. 3). (5) In contrast to our current study, Hayakawa et al. [36] failed to identify GLWamides, pQITRFamides, Antho-RNamides, −RIamides, −KAamides, and RWamides in *N. vectensis* (Table 2, neuropeptide families #3 and #5; Table 3, neuropeptides families #7 to #10; Additional files 3, 5, 7-10).

In conclusion, our study shows that three cnidarian neuropeptide families, the X_1_PRX_2_ amides, GRFamides, and GLWamides, have likely originated in the common cnidarian ancestor (Fig. 3), while other neuropeptides are confined to a certain cnidarian class, subclass, or order. In addition, our paper contributes to the creation of neuropeptide inventories for hexacoral species. These inventories might be useful resources for subsequent experiments that will improve our understanding of hexacoral laboratory models, such as *N. vectensis*, *E. diaphana*, and *A. millepora*.

## Methods

### Sequence data

For our analyses, we used the assembled genome and transcriptome sequence data from the hexacorals and ceriantharians given in Table 1. Here, also the accession numbers for each of the databases are given.

### Identification of neuropeptide preprohormones

To identify neuropeptide preprohormones, we applied a bioinformatics tool that we published two years ago [37, 80]. In short, this script is based on identifying multiple prohormone convertase cleavage sites (KR, RR, R) in conjunction with a Gly residue (GKR, GRR, GR) for peptide C-terminal amidation. Using this script, we selected proteins from the databases as being potential neuropeptide preprohormones when (i) they contained at least three of the above-mentioned processing sites, (ii) contained at least three highly similar peptide sequences preceding these processing sites, and (iii) contained a signal peptide for RER translocation [81, 82].

We also applied TBLASTN [83] and the Python subprogram Regex [84], using previously identified cnidarian neuropeptides or neuropeptide preprohormones as queries [9–11, 21, 24, 25, 27, 34, 52–58, 61, 63].

## Supplementary information

**Additional file 1.** Partial or complete amino acid sequences of the GPRGamide preprohormones in species belonging to the orders Actiniaria, Scleractinia, Corallimorpharia, or Zoantharia (belonging to the subclass Hexacorallia), or the order Spirularia (belonging to the subclass Ceriantharia).

**Additional file 2.** Partial or complete amino acid sequences of the Antho-RFamide (pQGRFamide) preprohormones in species belonging to the orders Actiniaria, Scleractinia, Corallimorpharia, or Zoantharia belonging to the subclass Hexacorallia), or the order Spirularia (belonging to the subclass Ceriantharia).

**Additional file 3.** Partial or complete amino acid sequences of the Antho-LWamide preprohormones or related preprohormones in species belonging to the orders Actiniaria, Scleractinia, Corallimorpharia, or Zoantharia (belonging to the subclass Hexacorallia), or Spirularia (belonging to the subclass Ceriantharia).

**Additional file 4.** Partial or complete amino acid sequences of the Antho-RPamide preprohormones or related preprohormones in species belonging to the orders Actiniaria, Scleractinia, Corallimorpharia, or Zoantharia (belonging to the subclass Hexacorallia).

**Additional file 5.** Partial or complete amino acid sequences of the pQITRFamide preprohormones or related preprohormones in species belonging to the orders Actiniaria, Scleractinia, and Corallimorpharia (belonging to the subclass Hexacorallia).

**Additional file 6.** Partial or complete amino acid sequences of the FHIRamide preprohormones or related preprohormones in species belonging to the orders Actiniaria, Scleractinia, Corallimorpharia, or Zoantharia (belonging to the subclass Hexacorallia).

**Additional file 7.** Amino acid sequences of the Antho-RNamide preprohormones in species belonging to the orders Actiniaria and Spirularia

**Additional file 8.** Amino acid sequences of the Antho-RIamide or related preprohormones in species belonging to the order Actiniaria.

**Additional file 9.** Amino acid sequences of the Antho-KAamide or related preprohormones in species belonging to the order Actiniaria.

**Additional file 10.** Amino acid sequences of the Antho-RWamide or related preprohormones in species belonging to the orders Actiniaria and Scleractinia.

**Additional file 11.** Scan of two pages from the Ph.D. thesis by: Carstensen K. Struktur, Wirkungsweise und Biosynthese von Antho-RPamiden, einer neuen Neuropeptidfamilie aus der Seeanemone Anthopleura elegantissima BRANDT. 1993; Ph.D. thesis, Faculty of Biology, University of Hamburg (written in German).

## Data Availability

All protein sequences from Additional files 1, 2, 3, 4, 5, 6, 7, 8 and 9 have been retrieved from publicly available genomic and transcriptomic databases (see Table 1).

## Abbreviations

A-RFamide: Antho-RFamide
A-RIamide: Antho-RIamide
A-RNamide: Antho-RNamide
A-KAamide: Antho-KAamide
A-RWamide: Antho-RWamide
Hyp: Hydroxyproline
MMA: Metamorphosin A
PC: Prohormone convertase
Phelac: L-3-phenyllactyl
pQ: Pyroglutamate residue (cyclized glutamine residue)
Regex: Regular expression (software program)
RER: Rough endoplasmic reticulum
RP-1: Antho-RPamide-1
RP-2: Antho-RPamide-2
RP-3: Antho-RPamide-3
RP-4: Antho-RPamide-4
