# Supplementary material for: A comparative genomics study of neuropeptide genes in the cnidarian subclasses Hexacorallia and Ceriantharia
Source: BMC Genomics. 2020 Sep 29;21:666. doi: 10.1186/s12864-020-06945-9 (PMC7523074; doi:10.1186/s12864-020-06945-9)
Supplement: Supplementary file 1 — Additional file 1. Partial or complete amino acid sequences of the GPRGamide preprohormones in species belonging to the orders Actiniaria, Scleractinia, Corallimorpharia, or Zoantharia (belonging to the subclass Hexacorallia), or the order Spirularia (belonging to the subclass Ceriantharia). [file 12864_2020_6945_MOESM1_ESM.pdf]

Additional file 1. Partial or complete amino acid sequences of the GPRGamide preprohormones in species belonging to the orders Actiniaria, Scleractinia, Corallimorpharia, or Zoantharia (all part of the subclass Hexacorallia), or the order Spirularia (belonging to the subclass Ceriantharia). For some species more than one preprohormone fragments was identified. Signal sequences are underlined. An asterisk indicates a stop codon. Neuropeptide sequences are highlighted in yellow; C-terminal processing sites are highlighted in green. The C-terminal Gly residues that are converted into C-terminal amide groups are highlighted in red.

Actiniaria (see Table 2, neuropeptide family number 1)

Anthopleura elegantissima

>GBXJ01018913.1 TSA: ANTHOPLEURA ELEGANTISSIMA COMP26526_C0_SEQ1 TRANSCRIBED RNA SEQUENCE

MPPKYDFVVLLFGIVVLSILSNSHQVYSAYLVQGGVQRDFGDWYNNYKPYYETEEYNKQTEEELEKNPLEPGR
DNNNQNDGPRGGRSLGELRGRGMLDGPRGGRSIDINGPRGGRSAEGPRGGRSTEYGPRGGRSAVYGPRGGRSM
EYGPRGGRSMQYGPRGGRSMEYGPRGGRSMEYGPRGGRSSEYGPRGGRSMEYGPRGGRSMKYGPRGGRSLMFG
PRGGRSLINGPRGGRSLNAHRIKKGIHAGPRGGRSVTKSGPRGGRSVSRMRRGIEEDGPRGGRDIENEGPRGG
RSM

Anemonia viridis

>GGLT01029780.1 TSA: Anemonia viridis TR18012:c0-g3-i1 transcribed RNA sequence

HQGEAVLGRSLEYGPRGGRSLEYGPRGGRSLVSGPRGGRSLMFGPRGGRSLVYGPRGGRSLNAHTIKKKIHAG
PRGGRSATKSGPRGGRGISRMRRSIEEDGPRGGRDVENQGPRGGRSMSDGNRGGRFMEDGPRGGRSMQDDGPR
GGRSMEEDGPRGGRSMEDGPRGGRSMEEDGPRGGRSMEDGPRGGR

>OCZR010600796.1 Anemonia viridis genome assembly, contig: scaffold600796_len163_cov32_single, whole genome shotgun sequence

MAPKYDFVCLLFGIVVLTIVSNSQEVYSAYMVQGGVQRDFGDWYNNYKPYYETEEYNKQTEEELEKNPLEPRK
FNNDQIDGPRGGRSLSELNVGGTLDGPRGGRSIDIKGPRGGRSALYGPRGGRSMEYGPRGGRSMEYGPRGGRS
MGYGPRGGRSMEYGPRGGRSLEYGPRGGRSMEYGPRGGRSVEYGPRGGRSLEYGPRGGRSLEYGPRGGRSLVS
GPRGGRSLMFGPRGGRSLVYGPRGGRSLNAHTIKKKIHAGPRGGRSATKSGP

Nematostella vectensis

>HADP01095596.1 TSA: Nematostella vectensis, contig TR53312|c0_g1_i1, transcribed RNA sequence

MAYFKWTLCAFVLAVLCLSTNYVAGEKNEEKSEKSTLLQELSKKDFDKELAADEAIVLELLKEKADDLDEESL
LDLLAPRGGRDAPRGGRSLLDAPRGGRSLLDLLAAPRGGRDAPRGGRSLTELLNAPRGGRSLADLLDAPRGGR
SLAELLDAPRGGRSIEAPRGGRSLAELLEAPRGGRSLIELLEAPRGGRSVEAPRGGRSILELLTAPRGGRSAP
RGGRSAEKSDAIHKEKKAPRGGRKRRSLPEEEMDGPRGGRSAVSGRSSEDGPRGGRAVYGPRGGRSFDGSRGG
RSYDGPRGGRSMENGPRGGRATEFGPRGGRSYEGSRGGREIDGPRGGRSFKEGPRGGRAVEGPRGGRDLYEEG
PRGGREVYLEGPRGGRDLYEEGPRGGREVYLEGPRGGR

Phymanthus crucifer

>WUCR01005850.1selectionselectiontranslationframe+1-1

MTLKCDFVILLFGILVLAILSDDSHVVYSAYLVQGGVQRDFGDWYNNYRPYYETEEYSKETENELEKNPLQPR
RYNDQNEGPREGRSVGMSDGQRGGRSIDIKVPRGGRSTEGPRGGRAIEYVHGRSTAYGSRGERSAVYGLRGGR
SLAYGPRGGRSLEYGPRGGRSLEYGPRGGRSLASGPRGGRSLMYGPRGGRFIVYGPRGGRSLENGLYKNTQRI
KKGMYAGPRGGRSVTKSGPRGGRSISRMRRSLEVEGQRGRRDLENEGPREGRSMEEGPRGGRGMEEEGPRGGR
SMFDDGPRGGRSVFDGPRGGRSMENGPRGGRSMEDGTLRGGRSMEDGPRGGRSVFDVPRGGRSMEDGPRGGRS
MDDGTRGGRDMEDGPRGGREILQNEDAVDNKNGPRGGRDEAADSAGRSRGGRDVTGREGALVRKKRVSDINME
AIHAKLKKMR*

Scolanthus callimorphus

>GGGE01112931.1selectionselectionrevtranslationframe+1

MELKWLFILVIAANLGQWQKCVFAESSSDKASKSSAKLLQDLGELKDKKSLEEELAADEAVILELLKDKADDE
NEDESLLDILAPRGGRDAPRGGRSLFDAPRGG

Exaiptasia diaphana

>NW_018386067.1 EXAIPTASIA PALLIDA ISOLATE CC7 UNPLACED GENOMIC SCAFFOLD, AIPTASIA GENOME 1.1 SCAFFOLD3160, WHOLE GENOME SHOTGUN SEQUENCE

MPFKKGFLLCLALELTIILCEARDVQRAYIGQRDFGDWYNNYEPYYEKQGSNKEPEEQDFNFWDENDGPRGGR
RSLDYQNGREINMYDGPRGGRSILENGPRGGRSLENGPRGGRSLSSNGPRGGRSLGYGPRGGRSIYDGPRGGR
SVYYGPRGGRSIEYGPRGGRSLQYGPRGGRSLRNGPRGGRSLKRMQRAGPRGGRSLAQMGPRGGRSLSAKLRM
KRNVLEDGVHGDRSVFNVFDGPRGGRSMEDEFGPRGGRSMDGPRGGRDESEGPRGGRDESEGPRGGRDEAEGP
RGGRDEAEGPRGGRDEAEGPRGGRDEGEGPRGGRSITHDGPRGGRDVSTKNIDSHSRTKRVVSQNQLKASS

Scleractinia (see Table 4, neuropeptide family number 1)

Acropora millepora

>Acropora millepora isolate SF001 amil.Sc0000002, whole genome shotgun sequence

MSFNSLKLLFLASIFTVLMLKERRILADGQDQKTVAKRDEVFYSNVESVKNSYSVEGDGKSRRSAEDEENQGE
ITDDGMMYYGSFYPMDTNTKGYELYYPENPPYEEWYGPFEPPEEYENQGEWYEGNGYYKRSLDFLGPRGGRSV
YYGPRGGRSLENEAGPRGGRSLNDEVMGSRGGRTIGNAQGPRGGRSVENGGPRGGRSVDTLAKGRLGVEDSEE
NKSTTRTFKKSDTSSIKGQRRGREIKADVGPRGGRGVSDSKTTDDVSNGAPGRRDARSASGPRGGRDASERDS
REQRARSVLDFLGPRGGRSIEFGPRGGRSTIFESPILWDETAFDYGPRGGRYIDYVLWRRSLETPPRDRRDII
FGPRGGRGILSGPRGGRAIDYGPRGGRSLEGYCSRCARSINFAEYGPRGGRSIEMGPRGGRSVDFGPRGGRSL
SEGPRGGRSLLFNTFAFSGPRGGRSVNYDLYEGPRGGRAIDERELSRAFGNEGAVYYYGPRGGRSVYGETGPR
GGREVEYTGPRGGRAVIYGPRGGRAIFSGPRGGRDISGPRGGRNIESGPRGGRSTKSDFGPRGGRSILSESAM
WSKTGSLRGRSSDILGPRGGRSTLESSATEKREATSSVEEQTITNEKVRKERDTNVDMEKIDKKVTKSK

Acropora digitifera

>NW_015441057.1selectionselectiontranslationframe+1

MSFNSLKLLFLASIFTVLMLKERRVLADGQDQKTVAKRDEVFYSNVESVKNSYSVEGDGKSRRSAEDEENQGE
ITDDGMMYYGSFYPMDTNTKGYELYYPENPPYEEWYGPFEPPEEYKNQGEWYEGNGYYKRSLDFLGPRGGRSV
YYGPRGGRSLENEAGPRGGRSLNDKVMGPRGGRTIGNAQGPRGGRSVRGGRGVSDSKTTDDVNNGAPGRRDAR
SAGGPRGGRDASERDSREQRARSVLDFLGPRGGRSIEFGPRGGRSTIFESPILWDERAFDYGPRGGRYIDYVL
WRRSLETPPRDRRDIIFGPRGGRGILSGPRGGRAIDYGPRGGRSLEGYCSRCARSINFAEYGPRGGRSIEMGP
RGGRSVDFGPRGGRSLSEGPRGGRSLLFNTFAFSGPRGGRSVNYDLYEGPRGGRAIDERELSRAFGNEGTMYY
YGPRGGRSVYGETGPRGGREVEYTGPRGGREVIYGPRGGRAIFSGPRGGRDISGPRGGRNIESGPRGGRSTKS
DLGPRGGRSILSESAMWSKTGPRGGRSLESSDILGPRGGRSTVESSATEKREATSSVEEQTITNEKVRKERDT
NVDMEKIDKKVTKSN

Mantipora capitata

>RDEB01000433.1 MONTIPORA CAPITATA ISOLATE COLONY #1 SC0000432, WHOLE GENOME SHOTGUN SEQUENCE

MGPSGLKLVLCLLILTVWESKKRSVLADEQSKKTVAKRDEVYYNNVGSVKNSYSVAEEEKSARSTENQENQGE
QGENTDQRLMYYGPFYPRYIDTEGYELYYPENPPYGEWHGPIEPPEENYETPDGWYFKRSLDFLGPRGGRSLY
YGSRGGRSLENEAGPRGGRSLNDVVMGPRGGRSVENTEGPRGGRSVEHLTVDIDGPRGGRSVKETGPRGGRTV
QESRETKSTIKTVQKSDSALTKGLRGGREIKADAGPRGGREVSDFKATEDVNTGPRGGRDTQSASGPRGGRDV
IEKGPREERVRSVSDFLGPRGGREIEYGTRGGRWVRNGPRGGRSIDFVSRDERFASFEGLGPRGGRALDYGPR
GGREVGYGPRGGRALGYGPRGGRAIAYGPRGGRAIDYGPRGGRSLEEYGSRGSRSIYVVEYGPRGGRSIGMGP
RGGRSVDLGPRGGRSLSEGPRGGRSLLFESVLGPRGGRSADYNLQDGPRGGRDLAHIYGSRGGRAVEYIDMYG
PRGGRALVEGEYGPRGGRAVEYEGEGYYYGPRGGRAIYVETGPRGGRGVEYEAGPRGGRALTYGPRGGRAILS
GPRGGRDISGPRGGRDVDNGPRGGRSTNTVWGPRGGRSILSEGAMWTKSGTSSAGPRGGRSLGSNEKLGPRGG
RSNVESNAAKKRETSSSVEGQTTSNKEVRKERDADLDMEKVDKKLTDSI

Pocillopora damicornis

>GEFF01009230.1 TSA: POCILLOPORA DAMICORNIS CONTIG_12117 TRANSCRIBED RNA SEQUENCE

MKNGALALFFCILSLTRVFAEEQTTQQKKTVAKRDEVFYNNVGSVKNSYSIEEEDRSSRSAEKEENQNNNSEN
KGEYLYYGSVYPRDFEGREYELNYPTYEEWYGPFEPPEEYYQNVDGDYKRSLYSRGKRSAYYGPRGGRSVYHG
PRGGRSVATGAGPRGGRSVENDSGPRGGRSVESNIGPRGGRSVESNIGPRGGRSVESDIGPRGGRSVEDSSYL
SNTNVGPRGGRSTDASGPRGGRSVDKSSQKSDASSTAETGPRGGRAVSDTKSKGDEANGPRGGRDTQSAINDQ
RGGRDVGEKGPREERVRSVENNGPRGGRSVESGPRGGRAIDSGPRGGRAIEYGPRGGRSVFYGPRGGRAIEYG
PRGGRSVDYGPRGGRSVDYGPRGGRAIDYGPRGGRAIEYGPRGGRAIEYGPRGGRSVDYGPRGGRTIDYGPRG
GRAIEYGPRGGREVYYGPRGGRDIDYGPRGGREIYYGPRGGRAIDFGPRGGRAVDYGPRGGRAVEYGPRGGRA
VEYGPRGGLSVDFGPRGGRSLLYDVVFGPRGGRSLAYEPRDRRFVESEGPRGGRDIDFEYGPRGGRDLEYVDV
YGPRGGRAAGGIDFGPRGGRDLEYSEEEGFYDFGPRGGRAVYFETGPRGGRSVEFETGPRGGREVEYGPRGGQ
YLYSGPRNGRDISGPRGGRSTTVAERSNGPRGGRDLENGPRGGRSAMAGPRGGRGILSEGSMWAASSGETNDG
PRGGRSVGKNTADASGPRGGRSTAESSTAVKREATTAGKEQTVVKERQERDSKEVNVDVEKVDKKLTNSS

Stylophora pistillata

>GARY01012958.1selectionselectiontranslationframe+1

MKTGASALIFCILSLNRVFAEDQTTQLKKTVAKRDEVYYNNVGSVKNSYSIEGEDRTSRSAENEEHQNNNAEN
KGEYLYYGSVYPRDFEGREYELNYPSYEEWYGPYEPSEEYYQNGEEDYKRSLYSFGPRGGRNAYYGPRGGRSA
YYGPRGGRSVYYGPRGGRSLEKEAGPRGGRSVENDDGPRGGRSVENDSGPRGGRSVENDVGPRGGRSVESNFG
PRGGRSVEDNIGLSGRRSVEDSSSSSNTNIGPRGGRSTDASGPRGGRSVEKSNQNSDASSTAKTGPRGGRDTE
SGPRGGRAVSDTKGKADEANGPRGGRDAQSEANGPRGGRDVGEKGPREERVRSVENDSLRGGKSVESGPRGGR
AVESGPRGGRAIEYGPRGGRSVFYGPRGGREIEYGPRGGRSVDYGPRGGRAIDYGPRGGRAIKYGPRGGRAID
YGPRGGRAVGYGPRGGRAIDYGPRGGRAIEYGPRGGREIFYGPRGGRDQDYGPRGGREISYGPRGGRAIDFGP
RGGRAVEYGPRGGRAVEYGPRGGRWIDFGPRGGRSLLYDVVFGPRGGRSLAFGPRGGRSVEFEGPRGGRDIDF
ENGPRGGRDLEYVDVYGPRGGRAVGGMDFGPRGGRDLEYSEEEGFYEFGPRGGRAVYFEAEPRGVRGVEFQTG
PRGGREVEYGPRGGRYIYSGPRGGRDISGPRGGRSATTAESSNGPRGGRDLENGPRGGRSAVAGPRGGRAILS
EGSMWAASSGPRGGRSLDKNTADASGPRGGRSTAESSKTVKRETTAAGKEQTAVKERQERDSKEVDVDIEKV
DKLS*

Orbicella faveolata

>NW_018148679.1 ORBICELLA FAVEOLATA ISOLATE FL UNPLACED GENOMIC SCAFFOLD, OFAV_DOV_V1 SC7JCM8_1245, WHOLE GENOME SHOTGUN SEQUENCE

MKTEALAFLICALFLRETTADEQVAQSVEQKKTVAKRDEVFYNNVGSVKNSYRVSGAERRSAENQENQANRAE
STNSELLYYGSFYPRDFEGREYELYYPENPSYGEWYGPFEPPVDYYENGEESWDGRDYKRSFDFIGPRGGRSV
YYGPRGGRYVYYGPRGGRSAESEAGPRGGRSVENSNGPRGGRSAENDNGPPRGRSAEDESVTLNTNTGPRGGR
AVNNDGPRGGRAVDGSQTKRSTTTEQKLDTKSTTENGPRGGRDTAADAGPRGGRAISVKKASSDEASGPRGGR
DTQSAASGPRGGRDVGVKNPREERVRSVENDGPRGGRSVLFGPRGGRAVNSGPRGGRAIEYGPRGGRSVFYGP
RGGRAIEFGPRGGRAVDFGPRGGRAVDFGPRGGRAIDYGPRGGREIYYGPRGGRAIEYGPRGGRTIEYGPRGG
RAIGYGPRGGRSLLYGPRGGRSMETYGLRGGRAIDFGPRGGRAVENGPRGGRSLAFGPRGGRSVGDGPRGGRS
LDLGPRGGRSLFEGPRGGRSTLYDIVYGPRGGRSAEYDGPRGGRDLDYIFGPRGGRSLGYVDIYGPRGGRALE
EIDFGPRGGRTVDYVGDGYYDFGPRGGRAIYYETGPRGGRGIEFEAGPRGGRGVEYRLFSGPRGGRDISGPRG
GRSAAYFEISNGPRGGRAVESGPRGGRNAVINSMSGPRGGRSILSEGTMWTNSGASNTGPGGGRSVSNSKAEK
AGPRGGRSTSENKKTEKREATSTTAAEKTLAKKREERDSKEADVDLQKIDKQLTKSS

Callimorpharia (see Table 6, neuropeptide family number 1)

Amplexidiscus fenestrafer

>scaffold_680selectionselectiontranslationframe-1

MERRVLGLLVCAVLLKGLNFGQNDGCAAEKGVQVTLNGRTKTVAKRDEVVYSNVGSVKNSYSLESG
QEGRRSAASPEQQKYNERNTDTEMLYYRPLYPRDRGSREYVLYYPEYSPHREFYEPYQPAQSYYGN
EQENYGEEMALPEVYYGISEYKRSFNGARGGLDALNEYNNGPRGGRSARSITGPRGGRSVVEDSRQ
SAGPRGGRSLESKTGLGAVGKADTSSNLENGPRGGRAINDDGPRGGRSVGTATGPRGGRASEDGPR
GGRSTTKLQDKRTTAEEKNTNDVSSTKNGPRGGREIKERTGAMGGGAIAVSGDAAKDSSASSDQSN
GLRGGRGTESTSGPRGGRDVGNIIAPREERVRSIADNPKYMYYGPRNGRAMSLGPRGGRAVENGPR
GGRSLAWAVEYGPRGGRSVEYGPRGGRAIDYGPRGGRSLVYGPRGGRSIVNYGPRGGRAVEYGPRG
GRALMYGPRGGRSVTDYGPRGGRAIGYGPRGGRSMIYDGPRGGRATYYGPRGGRSTVNGPRGGRSL
EYGPRGGRSVYMGTRGGRSLMFGPRGGRAVQFELGLRDGRSAATNFGPRGGRSFYDEVVGPRGGRS
FASTEMYGPRGGRSVGYRYQMYSGPRGGRGLLYNEVYGPRGGRAFEDSEFGPRGGRDLEQAGGYYR
TKGYYGYGPRGGRDVYYEGGPRGGRAVGYETVFGPRGGREVSGPRGGRSATTENSVNYSGPRGGRS
IESDASAGPRGGRSILSQSALGEHSDDTAAKAGPRGGRSLGSSSSDSTAGPRGGRSMVKQEEHVGG
PRGGRSSSDSAVSNNGTTDGQLAGDKKRDESVAKRADTDMEKVDSKVEKSS*

Corynactis australis

>GB|GELM01056998.1| TSA: CORYNACTIS AUSTRALIS COMP84402_C0_SEQ1 TRANSCRIBED RNA SEQUENCE

EYGPRGGTSMTDYGPRGGRAVDLGPRGGRSMENGPRGGRSLEYGPQWRRSLLFGPRGGRSVQSESGPRGGRSV
ATSFGPRGGRSLSVGPRGGRSALFDTVFGPRAGRSFSSEEAYGPRGGRSEYEMYSGPRGGRGVDYGYQYGPRG
GRALNYEEMYGPRGGRALGEFGPRGGRDLEYGSYYEGEGYYSNGPRGGRAVYFEEGPRGGRALGYETGFGPRG
GREVFNGPRGGRAISGPRGGRSTASMEHTAEDSTGPRGGRSTNSDTSSAGPRGSGRSILSESAMRSDSGSAAG
AAAGPRGGRSLGAGQSDSAAGPRGGRSTVTVTDQQKDVGGPRGGRSTSDSSVTVKKSTVEEQQADGKQREERE
MKQADIDMAKVDKQLSKSS

>GB|GELM01041161.1| TSA: CORYNACTIS AUSTRALIS COMP66920_C0_SEQ1 TRANSCRIBED RNA SEQUENCE

LENSAQTTGINTGPRGGRSVDSTNGPRGGRSADGAAAATSSKEGPRGGRAVEEDGPRGGRSVETANGPRGGRA
AADGPRGSGRSVQESPEKRTTEKEQNTNDASSTSNGPRGGRDIQDNSGTVKDSSTSSNQASGPRGGRDTQSTS
GPRGGRDVVNEKAPREERVRSVADSPKYTYHGPRNGRAFFLGPRGGRSVAFGPRGGRALENGPRGGRSLEYGP
RGGRAVNFGPRGGRDLEYGPRGGRSMIDYGPRGGRAIDYGPRGGRSLEFGPRGGRAVYLGPRGGRSLEYGPRG
GRSFIDYGPRGGRAI

Discosoma sp.

>scaffold_52selectionselectiontranslationframe+1

MERRVLRLLLCAILLKGLNIGQNDVCAAEQTVQVALNGRTKTVAKRDEVVYSNVGSVKNSYSIERGQEGRRSE
ESQEQQEYNERNTDNEMLYYRQPYPRDREGRGYEMYYPEYSPDREFYQPYEPVESYYNGNEQENYGGEMALPD
VYYGLSEYKRSFNGARGGLGTLNEYYDGPRGGRSVQSITGPRGGRSVVEDSTQSVGPRGGRSIESKTGPRGGR
SAADDADTSANLEDGPRGGRAVNDDGPRGGRSAGTVTGPRGGRASEDGPRGGRSTTASQDKKTLADEKNTDDV
LSTKNGPRGGREIKEGTGAMGGRAIVVRGDAAKDSSASADQSNGPRGGRDTQSTSGPRGGRDVGNKIAPREER
VRSIADNPKYMHYGPRNGRALVLGPRGGRAVENGPRGGRAVGWAVEYGPRGGRSLEYGPRGGRAIDYGPRGGR
SLGYGPRGGRSMVNYGPRGGRAVEYGPRGGRTLAYGPRGGRSMIEYGPRGGRAIDYGPRGGRSVEFGPRGGRS
LEYGPRGGRSLTDYGPRGGRAIGYGPRGGRSMTYYGLLGGRATDYGPRGGRSTVEGPRGGRSLEYGPRGGRSV
YLGPRGGRSLLFGPRGGRSVQLQFGPRGGRSVVTAFGPRGGRSLYDAVVGPRGGRSFTSTEMYGPRGGRSVGY
QIYNGPRGGRGVDYSYVYGPRGGRAIEGGEFGPRGGRALDQAGGYYRTEGYYDYGPRGGRDVYYEGGPRGGRA
VGYETGFGPRGGREVSGPRGGRSATITENSVDSSGPRGGRSIDSDASAGPRGGRSIVSESAMSERSDDTVANA
GPRGGRSLGSGSSDSTAGPRGGRSTVNKEKQSGGTKGDRSSSDSANSNKSVADGQLAEDKKRDESVAKRADTD
MEKVDAKLEKSS*

Ricordea yuma

>GB|GELN01042514.1| TSA: RICORDEA YUMA COMP77941_C0_SEQ1 TRANSCRIBED RNA SEQUENCE

MDKRALWLLLCIVFLNEFEKRLVCAVDQTEKTVQNTKVKTVAKRDEVVYSNVGSVKNSYSFQGGQEERKSTGS
DQSEQQRENNKGNTDSEMLYYGPFYPRGSYVRRDELYYPEYPTYYEGYLGWYDPYRTTDNYYGEDGQETMGAS
DEYKGFTVYKRALSYNGPRGGRATMNEYIAGPRGGRSTYSTNGPRGGRSAVEDSVRTTGLRGGRSTEIVNGPR
GGRSANDAGTSSNVNDGPRGGRAVADSGPRGGRGVEMASRSRGGRASADGPRGGRSARDSQQKTATAQEKSAN
DASSTENGQRGGREVKSEAGPRGGRATSEGASTLQSSGPRGGRDVGDMSPREERVRSVSDNQNYVYSGSRNGR
DLYYGPRGGRTVFYGPRGGRALEYGPRGGRAVDYGPRGGRSLEYGPRGGRAVDFGPRGGRSLDYGPRGGRAVY
YGPRGGRAIEYGPRGGRSIIDYGPRGGRDIEELGPRGGRSLGYGPRGGRSFVDYGPRGGRAIDYDRSLEYGPR
GGRSLDYGPRGGRSIIYYGPRGGRAVDYGPRGGRSLQNGFLDGPRGGRSLTTDSGLRGGRSTLHDGPRGGRSF
SSEEMYGPRGGRGVDYSYGPRGGREVGYEELSGPRGGRDLAAGVFGPRGGRDLEHGFDYERGFYTFGPRGGRA
IYYDKGPRGGRAVGYEAEFGPRGGRDLFTGPRGGRDISGPRGGRSTTGAEHIENSNGPRGGRSATLDASLGPR
GGRTILSEGAMGARSASTTNEAGPRGGRSLESSSSDNHAGPRGGRSTLDQGKTSQGPRGGRSSSDSSNKEENE
TANKRDESHAKRAEVNVEKIDKQLSKSS

Zoantharia(see Table 6, neuropeptide family number 1)

Protopalythoa variabilis

>GCVI01021646.1 TSA: PROTOPALYTHOA VARIABILIS CL11579.CONTIG1 TRANSCRIBED RNA SEQUENCE

PRGGRSIDMGPRGGRSLDMGPRGGRSLDMGPRGGRSIDMGSRERRSLDMGPRGGRSIEGPRGGRSVFYESGPR
GGRAVDNGPRGGREIESDSGPRGGRDVVKGPRGGRDAEFDYGPRGGRDLSIKYGPRGGRDLAFDYGPRGGRSA
YDGPRGGRDINEGPRGGRSLEVDMYEGPRGGRDVDAGPRGGRSAVQDVKNVEDDGPRGGRAILANNDKTHTMK
DSSFKFDE

Zoanthus sp.

>GGTW01141341.1 TSA: ZOANTHUS SP. QL-2018 UNIGENE89435 TRANSCRIBED RNA SEQUENCE

MISIVKALLLAVFCYGICPMVLSSEKIKRGNYFDSNTGGRHGEEDENIREERVDNTGETRLKLHKRSIDNGER
MLRDLTDYSQYSYRYNGPFEYRYPYEPVGETNGNYEILGDQNSQGPRGGRSIINGPRGGRDVIYKGPRGGRSI
DMGPRGGRSVEMGPRGGRSVDMGPRGGRNIDMGPRGGRSIDMGPRGGRSIDLGPRGGRGVDIGPRGGRSVEMG
PRGGRSIDMGPRGGRSVEMGPRGGRGVDIGPRGGRSVEMG

>GGTW01041186.1 TSA: ZOANTHUS SP. QL-2018 CL16884.CONTIG1 TRANSCRIBED RNA SEQUENCE

PRGGRSVDMGPRGGRSVDMGPRGGRSVDMGPRGGRSFDAGPRGGRTLYDKYGINGKKSLDYGPRGGRGIETNN GPRGGRDVQSDNGPRGGRDVENENGPRGGRDVESDSGSRGGRDANSDNGPRGGRNVESENGPRGGRDLESSNG PRGGRDVESNNGPRGGRNVIDGPRGGRNADFEYGPRGGRDLSSKHGPRGGREASLVYGPPGGRDEAFDNGPRG GRSASGPRGGREVEQEEKQFNNHGLIEMGKRYESDQEKVKKEATKEITSKINDEMSH

Ceriantharia (see Table 6, neuropeptide family number 1)

Pachycerianthus borealis

>HAGY01013590.1 TSA: Pachycerianthus borealis, contig TRINITY_DN15170_c0_g3_i1, transcribed RNA sequence

MWDIRQKGVACLFLTCLSFNAFLCENLVDNSKDESRRSLKTIINLDSFSGPRGGRSLEKDGDSGPRGGRGLTEGYAGPRG GRELTEGISGPRGGRELTEGIAGPRGGRGLTEGIAGPRGGRGLTDGIAGPRGGRGLTEGIAGPRGGRGLT

>TSA: Pachycerianthus borealis, contig TRINITY_DN15170_c0_g3_i1, transcribed RNA sequence

RGGRGLTEGIAGPRGGRGLTDGIAGPRGGRSLTEGIAGPRGGRGLTEGIAGPRGGRGLTEGIAGPRGGRGLTE GIAGPRGGRELTEGFAGPRGGR
