# Supplementary material for: A comparative genomics study of neuropeptide genes in the cnidarian subclasses Hexacorallia and Ceriantharia
Source: BMC Genomics. 2020 Sep 29;21:666. doi: 10.1186/s12864-020-06945-9 (PMC7523074; doi:10.1186/s12864-020-06945-9)
Supplement: Supplementary file 2 — Additional file 2. Partial or complete amino acid sequences of the Antho-RFamide (pQGRFamide) preprohormones in species belonging to the orders Actiniaria, Scleractinia, Corallimorpharia, or Zoantharia belonging to the subclass Hexacorallia), or the order Spirularia (belonging to the subclass Ceriantharia). [file 12864_2020_6945_MOESM2_ESM.pdf]

Additional file 2. Partial or complete amino acid sequences of the Antho-RFamide (pQGRFamide) preprohormones in species belonging to the orders Actiniaria, Scleractinia, Corallimorpharia, or Zoantharia (all part of the subclass Hexacorallia), or the order Spirularia (belonging to the subclass Ceriantharia). For some species more than one preprohormone fragments was identified. Signal sequences are underlined. An asterisk indicates a stop codon. Neuropeptide sequences are highlighted in yellow; C-terminal processing sites are highlighted in green. The C-terminal Gly residues that are converted into C-terminal amide groups are highlighted in red.

Actiniaria (see Table 2, neuropeptide family 2)

Anthopleura elegantissima

This is the complete Antho-RFamide preprohormone from A. elegantissima that has been cloned by us in 1992 see reference [25] and https://www.ncbi.nlm.nih.gov/pubmed/1429603 :

>Antho-RFamide preprohormone

MTTVSYVTILLTVLVQVLTSDAKATNNKRELSSGLKERSLSDDAPQFWKGRFSRSEEDPQFWKGRFSDPQFWK
GRFSDPQFWKGRFSDPQFWKGRFSDPQFWKGRFSDPQFWKGRFSDPQFWKGRFSDGTKRENDPQYWKGRFSRS
FEDQPDSEAQFWKGRFARTSSGEKREPQYWKGRFSRDSVPGRYGRELQGRFGRELQGRFGREAQGRFGRELQG
RFGREFQGRFGREDQGRFGREDQGRFGREDQGRFGREDQGRFGREDQGRFGREDQGRFGRELQGRFGREFQGR
FGREDQGRFGREDQGRFGRELQGRFGREDQGRFGREDQGRFGREDLAKEDQGRFGREDLAKEDQGRFGREDIA
EADQGRFGRNAAAAAAAAAAAKKRTIDVIDIESDPKPQTRFRDGKDMQEKRKVEKKDKIEKSDDALAKTS*

This is the incomplete N-terminal fragment that could be recovered from the transcriptome:

> gb|GBYC01015085.1

MTTVSYVTILLTVLVQVLTSDAKATNNKRELSSGLKERSLSDDAPQFWKGRFSRSEEDPQFWKGRFSDPQFWK
GRFSDPQFWKGRFSDPQFWKGRFSDPQFWKGRFSDGTKRENDPQYWKGRFSRSFEDQPDSEAQFWKGRFARTS
TGEKREPQYWKGRFSRDSVPGRYGRELQGRFGRELQGRFGREAQGRFGRELQGRFGREFQGRFGRED

Anemonia viridis

>OCZR010052132.1 Anemonia viridis genome assembly, contig: scaffold52132_len524_cov50, whole genome shotgun sequence

MTTVSYVTLLLTVLVQVLTSDAKVTNNKRELSSGLKEQGPSDDAPQFWKGRFSRSEEVPQFWKGRFSDPQFWK
GRFSDPQFWKGRFSDPQFWKGRFSDPQFWKGRFSDPQFWKGRFSDPQFWKGRFSEGTKRENDPQYWKGRFSRS
FEDQPDSEAQFWKGRFARTSSGEKREPQYWKGRFSRGSVPGRLGRELQGRFGRELQGRFGRMAQGRFGREDPG
RFGREAQGRFGRELQGRFGREFQGRFGREDQGRFGRELQGRFGRENQGRFGREEQGRFGREIQGRFGREDQGR
FGREDQGRFGRELQGRFGREDQGRFGREDQGRFGRELQGRFGREDKGRFGREKLAEEDQGRFGREDLAEADQG
RFGREDIAKEDQGRFGREDIAKEDQGRFGRNAAAKKRTIDLIDIESDPKPQTRFDVKDMHEKRKVEKKDKIEK
SDDALAKTS

Nematostella vectensis

>scaffold_19

MAPRPGTLLLLGILIQVLICTAKSTYKKEIADLLDDNKDTPQFWKGRFSDPQFWKGRFSDPQFWKGRFSDPQF
WKGRFSDPQFWKGRFSDPQFWKGRFSDPQFWKGRFSDPQFWKGRFADELLNGGHHKHHHEEGEWKRTAGPGRF
GREDQGRFGREDQGRFGREDQGRFGREDQGRFGREDQGRFGREDQGRFGREDQGRFGREDQGRFGREDQGRFG
REDQGRFGREDQGRFGREDQGRFGREDQGRFGREDQGRFGREDQGRFGREDQGRFGREDQGRFGREDQGRFGR
EDQGRFGREDQGRFGREDQGRFGREDQGRFGRNKIARVIDLDQGRFGRTMDTATKKDTVASMPEQDANPQTRF
DGKKRQAAKERSIEKKSTISSDAKASDAKQS

Phymanthus crucifer

>WUCR01005850.1selectionselectiontranslationframe+1

MTKISYVTILLTVLVQVLTLNAKATNNKRELADAVNDDAPQFWKGRFARSQDSQFWKGRFSDPQFWKGRFSDP
QFWKGRFSDPQFWKGRFSDPQFWKGRFSDSQFWKGRFSDPQFWKGRFSDGGKRGNEPQFWKGRFSRSYNKQPA
SDAQFWKGRFARNEVPGRYGRDFQGRFGREFQGRFGREDQGRFGREVQGRFGRELQGRFGRELQGRFGREDLA
EDQGRFGREDLAEDQGRFGREDLTEDQGRFGREDLTEDQGRFGRPVKTGFRTCLVRPQLWTAVYQRKRPESAE
APFLAPTGASASRELVARPISVFDW

Scolanthus callimorphus

>GGGE01108082.1 TSA: SCOLANTHUS CALLIMORPHUS TRINITY_R1_DN172982_C0_G1_I2, TRANSCRIBED RNA SEQUENCE

RFGRDADQGRFGREQGRFGREQGRFGREQGRFGRDFQGRFGREEQGRFGREEQGRFGREMDQGRFGREMDQGR
FGREEQGRFGRDYQGRFGREQGRFGRDFQGRFGREQGRFGREQGRFGRDFQGRFGREEQGRFGRELEQGRFGR
DFYDEQGRFGRNMDSAEDNKRSDILEELVRDPQTRFDSERSVISEKDTKKH

Exaiptasia diaphana

>NW_018388004.1 EXAIPTASIA PALLIDA ISOLATE CC7 UNPLACED GENOMIC SCAFFOLD, AIPTASIA GENOME 1.1 SCAFFOLD630, WHOLE GENOME SHOTGUN SEQUENCE

MTTASYVTILVTLLFHILAINAKDTKREPEDDQPQFWKGRFARGAVPQYWQGRFSDPQFWKGRFADPQFWKGR
FADPQFWKGRFADPQFWKGRFSDPQYWKGRFSDDDKRSNDPQYWKGRFSRSMKTPDDDLPQFWKGRFSRDSLP
GRFGRELQGRFGREQGRFGREEQGRFGREEQGRFGREEQGRFGREEQGRFGREEQGRFGREEQGRFGREEQGR
FGREEQGRFGREEQGRFGREEQGRFGREQGRFGREEQGRFGREDQGRFGREEDQGRFGREEADQGRFGREEED
QGRFGREKEDQGRFGREEADQGRFGKRDLKAKSNSKRTIEVQSDAMPQTRFRDSFKRNLVKKEKKSADASSKA
T

Scleractinia (see Table 4, neuropeptide family 2)

Acropora millepora

>GHGU01103239.1 TSA: ACROPORA MILLEPORA COMP128196_C0_SEQ1, TRANSCRIBED RNA SEQUENCE

MSSMMVSTLVSLVCCLILSTNAKPLEENNASEFDFTEGKLVRSANDRLKRSSMENIGSLNDPQYWKGRFYDYV
HWRERPNNQEHNQVADSIVDKREPQYWKGRFYREQGVDQRARAFVPGRFGRNFQGRFGRNMQGRFGREDEQGR
FGREENLQGRFGREDDQGRFGREENMQGRFGREDDLQGRFGRDFQGRFGREEDLQGRFGREDDQGRFGREETQ
GRFGRDKVANDEEQGRFGREDRDDELKEFPKDFEEDEKADSAEKREVTSSLEESKEKNLES

Acropora digitifera

>NW_015441131.1selectionselectionrevtranslationframe+1

MSSMMVSTLVSLVCCLILSTNAKPLEENNASEFDFTEGKLVRSANDRLKRSSMENIGSLNDPQYWKGRFYDYV
HWRERPNNQEHNQVADSIVDKREPQYWKGRFYREQGVDQRARAFAPGRFGRNFQGRFGRNMQGRFGREDEQGR
FGREENLQGRFGREDDQGRFGREENMQGRFGREDDLQGRFGRDFQGRFGREEDLQGRFGREDDQGRFGREETQ
GRFGRDKVANDEEQGRFGREDPDDELKEFPKDLEEDGKADSAEKREVTSSLEESKGKNLES*

Mantipora capitata

>GFRO01002721.1 TSA: MONTIPORA CAPITATA C214820_G1_I1 TRANSCRIBED RNA SEQUENCE

MLSMMSMLVSLVCCLALLTNAKPLEETGNDRPKFTEAEFVRSVNDRQKRSTLEKIGSLNDPQYWKGRFYDYAH
WRDRHNDHGKDHVSDSVVDKREPQYWKGRFYREEGEAHHQTRAIAPGRFGRNFRGHFGRNIQGRFGRDSIQER
FGREDDEGRFGRENDQGRFAREENLLGRFERKEDQGRFGREENTLGQFGEEENLQERLGREDNLQGRFGREDD
QVRRFGREETQGRFGRDNIANGEEQGRFGRGYKDGNLKQFPQELEEEKEDSKDDKREVTSSLEETKEENSES

Pocillopora damicornis

>XP_027051563.1 antho-RFamide neuropeptides type 2-like [Pocillopora damicornis]

MSVSNTFILAVFCCHLLLVHAKALEDSTKEAADENDVPAFAEGKFTRSLENDPQYWKGRFSDIVGELSDPQYW
KGRFSHDQYWQGRFADTGSEMDKREPQYWKGRFSRGEEEQQLRSAVPGRFGRNFQGRFGRNFQGRFGRNFQGR
FGRELQGRFGRDEIQGRFGREDLQGRFGREDMQGRFGREEEDQGRFGRDFIQGRFGREDQGRFGREDDQGRFG
RDSIQGRFGREELDQGRFGREELDQGRFGRDEIVEDEDQGRFGREEDSDDLLVKLENKLEEDHDAKREVASSL
DESKDETSES

Stylophora pistillata

>GARY01004500.1selectionselectiontranslationframe+1

MSVSNSFIVAVLCCHLLLVYAKPLEDSKKEAADESDVPAFAEGKFTRSVENDPQYWKGRFSDFEGDLNDPQYW
KGRFSHDQYWRGRFADTGSDMDKREPQYWKGRFSREEEEHQLRSAVPGRFGRNFQGRFGRNFQGRFGRNFQGR
FGREMQGRFGREEMQGRFGREDLQGRFGREEEDQGRFGRDFIQGRFGREEQGRFGREYDQGRFGRDSIQGRFG
REELDQGRFGREELDQGRFGRDEILEDEDQGRFGREEDSDDLLVKLENKLGEDYDARREVVSSLDESKDETSE
S*

Porites rus

>Porites rus isolate 14846/IV/SATS-LN/2007 genome assembly, contig: sscaffold02543, whole genome shotgun sequence

MSAWIAVVLCCHLLVISAKPLEEKKESSDDSDSLPEFTDAKFERSVSDPQYWRGRLSDNKEALNDAQYWRGRL
SADPQYWRGRFSDAQYWRGRFSDNMEEKREPQYWFGRFSRGDREHQLRALVPGRFGRNFQGRFGRNFQGRFGR
ENMQGRFGREENMQGRFGREDDKQGRFGRDFQGRFGRDELQGRFGREKEDEQGRFGRNFQGRFGREEDLQGRF
GREKIADDKEQGRFGREEEDDLEKIEKTLQAEEDSKEEKPEEVRSLEDSKDESSES*

Orbicella faveolata

>NW_018149777.1 ORBICELLA FAVEOLATA ISOLATE FL UNPLACED GENOMIC SCAFFOLD, OFAV_DOV_V1 SC7JCM8_2955, WHOLE GENOME SHOTGUN SEQUENCE

MRISSCVLLAVFCCHLLLISAKPLDDENKEADDSDMPEFTEGKFARSVSAEKPQYWKGRFSDMKEEVLKDPQY
WKGRFSDPQYWKGRFSDPQYWKGRFSDVNKREPQYWKGRFSREEEGEQQVRALAPGRFGRNFQGRFGRNLQGR
FGRDMQGRFGREDMQGRFGREDDLQGRFGREEDALQGRFGREEDSQGRFGREEEQGRFGRDFMQGRFGREEED
QGRFGREEEMQGRFGREEDFQGRFGREEEMQGRFGRDEIAEDEDQGRFGREEDDLLAELENKLLDETYDTKRE
IATSSLEESNAESTES

Corallimorpharia (see Table 6, neuropeptide family 2)

Amplexidiscus fenestrafer

>scaffold_221selectionselectiontranslationframe+1

MSNLHLLILGAISCELVLLIQAKAIDETRDLSVGNVEPEFAGGDPQFWKGRLADWKKPLSDLWYDT
HDDEREPQFWKGRLHRDDVLESNQPIQGEYDAYEVRTIVPGRFGRDFLGRLRREEIQGRFGREEGI
QGRFGRGAQGRFGREEYQGRFGREENFQGRFGREEDMQGRFGREEDMQGRFGREADLQGRFGREED
QGRFGREEDFQGRFGREEMQGRFGREMQGRFGRENLQGRFGRENIQGRFGREEYLQGRFGREELQG
RFGREQGRFGRDKVEDDTEQGRFGREEDQGRFGREEDQGRFGREVDQGRFGREVDQGRFGREEDQG
RFGREEDQGRFGRNELLESSNDLFADGEKNLSEKDSEDLAHEDKREVNHGLEDKNDESSKS*

Corynactis australis

>GB|GELM01055713.1| TSA: CORYNACTIS AUSTRALIS COMP83249_C0_SEQ2
TRANSCRIBED RNA SEQUENCE

MSALYALLLTAIGCQLLLTYAKPLDETQENDEPKFAEGKFARAVNDPQYWKGRFSDWKGHLNDPQYWKGRFSD
PQYWKGRFSDPQYWKGRFSDTDLNKREPQYWKGRFSRDGTLEGVENHETRGLAPGRFGRNLQGRFGRDELQGR
FGREEDLQGRFGREEDLQGRFGREEDTQGRFGREEDQGRFGREELQGRFGREE

>GB|GELM01056293.1| TSA: CORYNACTIS AUSTRALIS COMP83775_C0_SEQ1
TRANSCRIBED RNA SEQUENCE

LQGRFGRDNLQGRFGREDMQGRFGREEDMQGRFGREEDMQGRFGREEEDMQGRFGREEDMQGRFGREQGRFGR
EQGRFGRDKVEADEEQGRFGREEDQGRFGRDELSDSAADDLLAEVEKNLFEDDIDEDKHEAESSLADTKDESS
QS

Discosoma sp.

>scaffold_2selectionselectiontranslationframe+1

MSNLCALILAAIRCEVVLLTQAKAIDETREPFTGNSEPDFAGGEIARSVNYTPFWKGRLAGREEPLNDQFWKG
RLYDASYDEREPQFWKGRLHSRDVLEDGQPIQGEHYEVRTIVPGRFGRDFLGRFRREEMQGRFGREEEMEGRF
DRGAQGRYGRGEGQGRFGREEGMQGRFGREVDLQGRFGREEGFQGRFGREEMQGRFGREEDQGRYGRDLQGRF
GRDNLQGRYGREKIQGRFGREDLQGRFGREESQGRFGREESQGRFGREQGRYGRDKVDDEAEQGRFGREEEQG
RYGRDELFDSSNDLVAKEEKNSGDLAHEVKHSIEDTNDESAKS*

Ricordea yuma

>GB|GELN01036197.1| TSA: RICORDEA YUMA COMP70150_C0_SEQ1 TRANSCRIBED RNA SEQUENCE

MSRALYAILLVATACHSLRAKAMDDKRELSSTNNEPDFADGEFARSLNDPQYWRGRFADWKEDLNDPQYWKGRFSDPQYWKGRFSDPQFWKGRFSDMESDKREPQYWRGRFSRNMLRNVPLGEGEQRVLRDFAPGRFGRDFQGRFGREELQGRFGREDLMQGRFGREEDLQGRFGRESDQGRFGREDLQGRFGREEDELQGRFGREENQGRFGRDDLQGRFGREEDLQGRFGREEELKEKYGREEDQGRFGREDLQGRFGRDSMQGRFGREEDLQGRFGREEDLQGRFGRDEIEDAKEYDQGRFGRDNLMDYTDGLLEQVEQNLAENEPDELKNDDKREVKSSLEDNKDEGTES

Zoantharia (see Table 6, neuropeptide family 2)

Protopalythoa variabilis

>GCVI01061335.1 TSA: PROTOPALYTHOA VARIABILIS UNIGENE37206 TRANSCRIBED RNA SEQUENCE

RFGREEQGRFGREEQGRFGRNRIKTENDKQGRFGKKSEIIKAVKKDKKSKITKDEANEKIKKVKKTEIGTEEDQGRFGKKSEIKHKDQGRFGKKSVITTDDDQGRFGRFMTKISEVATDERGKHGLFQREKITKNNKDESEQGRFGREKSADKKSVHAMPDEEKNVLKEVKREFDLKDDFGLAEHKKRTFTS

Zoanthus sp.

>GGTW01185537.1 TSA: ZOANTHUS SP. QL-2018 UNIGENE133649 TRANSCRIBED RNA SEQUENCE

MLSTWFLVSWLLLLDCLLLTPAEVLSKGKRSSLRNYLETLGSKSQLNDQYWKGRLADSKLGSEELDGSDAMDTIFENLWKKKLSDYNKNYDDNPQYWRGRFSRNNDPQFWKGRLARSKEEQGRFGREKQGRFGREEQGRFGREPKAQYWRGRFERGKEEQGRFGREKQGR

>GGTW01093753.1 TSA: ZOANTHUS SP. QL-2018 UNIGENE41832 TRANSCRIBED RNA SEQUENCE

EEQGRFGREEQGRFGREEQGRFGREFGREELETANDKQGRFGKKSAIMKADQKKKKSGITENEEIGKDKKSNIDKEDQGRFGKKSKIEYKSQGRFGKKSVIASEDDQGRFGRDRVGKNDKNDFIQRKTIAENYKGEDQGRFGRGKSSAYVDESHVIKNIDVQRSFDALKVEDSRFAGKRKRALKS

Ceriantharia (see Table 6, neuropeptide family 2)

Pachycerianthus borealis

>HAGY01069284.1 TSA: Pachycerianthus borealis, contig TRINITY_DN11290_c0_g1_i1, transcribed RNA sequence

MIKTVCLIASLSILATVCTSLENQYWKGRFSRNNIQGRFSRDHIPQYWKGRFSRRDEIPQYWKGRFSRDEIPS
LNEHESMNEQDEEKRELAGPMQAREVHDNVYEPMNDQYWKGRFMRLRAGGHKRQFSRGMEGGQTDYGMRDAVT
QGQDEEDRELVPAEHQEGQESEEERDSLVPMGQQEEAETEEERNLQSEEAQEQEEEEKRQQEGEEEEEEERDVK
NDDMQGRFARQGRFKKREENELDAAAKAFDQGRFRREEN
