# Supplementary material for: A comparative genomics study of neuropeptide genes in the cnidarian subclasses Hexacorallia and Ceriantharia
Source: BMC Genomics. 2020 Sep 29;21:666. doi: 10.1186/s12864-020-06945-9 (PMC7523074; doi:10.1186/s12864-020-06945-9)
Supplement: Supplementary file 3 — Additional file 3. Partial or complete amino acid sequences of the Antho-LWamide preprohormones or related preprohormones in species belonging to the orders Actiniaria, Scleractinia, Corallimorpharia, or Zoantharia (belonging to the subclass Hexacorallia), or Spirularia (belonging to the subclass Ceriantharia). [file 12864_2020_6945_MOESM3_ESM.pdf]

Additional file 3. Partial or complete amino acid sequences of the Antho-LWamide preprohormones or related preprohormones in species belonging to the orders Actiniaria, Scleractinia, Corallimorpharia, or Zoantharia (all part of the subclass Hexacorallia), or Spirularia (belonging to the subclass Ceriantharia). For some species more than one preprohormone fragment was identified. Signal sequences are underlined. An asterisk indicates a stop codon. Neuropeptide sequences are highlighted in yellow; C-terminal processing sites are highlighted in green. The C-terminal Gly residues that are converted into C-terminal amide groups are highlighted in red.

Actiniaria (see Table 2, neuropeptide family 3)

Anthopleura elegantissima

This is the complete MMA preprohormone sequence that we cloned in 1995 from A. elegantissima, see reference [27] and DOI: 10.1073/pnas.92.25.11647

MALKCHLVLLAITLLLAQCSGSVDKKDSTTNHLDEKKTDSTEAHIVQETDALKENSYLGAEEESKEEDKKRSA
APQQPGLWGKRQKIGLWGRSADAGQPGLWGKRQSPGLWGRSADAGQPGLWGKRQNPGLWGRSADAGQPGLWGK
RQNPGLWGRSADAGQPGLWGKRQNPGLWGRSADARQPGLWGKREIYALWGGKRQNPGLWGRSADPGQPGLWGK
RELVGLWGGKRQNPGLWGRSAEAGQPGLWGKRQKIGLWGRSADPLQPGLWGKRQNPGLWGRSADPQQPGLWGK
RQNPGLWGRSADPQQPGLWGKRQNPGLWGRSADPQQPGLWGKRQNPGLWGRSADPQQPGLWGKSPGLWGRSAD
PQQPGLWGKRQNPGFWGRSADPQQPGLWGKRQNPGLWGRSADPQQPGLWGKRQNPGLWGRSADPQQPGLWGKR
QNPGLWGRSADPQQPGLWGKRQNPGLWGRSAGSGQLGLWGKRQSRIGLWGRSAEPPQFEDLEDLKKKSAIPQP
KGQ*

This is the incomplete MMA preprohormone sequence that we recovered from the transcriptome. Its corresponds to a piece in the N-terminal part of the completely cloned preprohormone shown above:

>GBYC01023131.1 TSA: ANTHOPLEURA ELEGANTISSIMA COMP61956_C0_SEQ2 TRANSCRIBED RNA SEQUENCE

QSPGLWGRSADAGQPGLWGKRQNPGLWGRSADARQPGLWGKREIYALWGGKRQNPGLWGRSADPGQPGLWGKR
ELVGLWGGKRQNPGLWGRSAEAGQPGLWGKRQKIGLWGRSADPLQPGLWGK

Anemonia viridis

>GGLT01019281.1 TSA: Anemonia viridis TR10585:c0-g1-i1 transcribed RNA sequence

MRKKRDTTEAKIAQVTEESKENSYLGSAEEESKKEDNKKSAAPQHPGLWGKRQHPGLWGRSADPLQPGLWGKR
QNPGLWGKREIIGINGRKRP

>GGLT01005731.1 TSA: Anemonia viridis TR3046:c0-g1-i1 transcribed RNA sequence

PQQAGLWGKRQNPGLWGRSADPQQAGLWGKRQNPGLWGRSADPQQAGLWVNSHAFLFFFHSMCYTTLQW

>GGLT01057729.1 TSA: Anemonia viridis TR36133:c1-g1-i2 transcribed RNA sequence

GLWGKRQNPGLWGRSADPQQAGLWGRSAGSGKRQERIGIWGRSAEPPQYKELEDLKKKISNSKSKRTMMMP

>OCZR010467576.1 Anemonia viridis genome assembly, contig: scaffold467576_len116_cov35_single, whole genome shotgun sequence

PGLWGRSAEPQQAGLWGKRQNPGLWGRSADPQQPGLWGKRQHPGLWGRSADPQQAGLWGKRQN

Nematostella vectensis

>HADO01000933.1selectionrevtranslationframe+1

GPPGLCRKRSPKPPGLWGKRQAGAPGLWGKRSAGPPGLWGKRDAGPPGLCRKRSPKPPGLWGKRQAGAPGLWG
KRSAGPPGLWGKRDAGPPGLWGKRVAGPPGLWGKRQAGAPGLWGREAGAPGLWGKRQAGAPGLWGKREAGAPG
LWGREANAPGLWGKRRAGAPGLWGKREANAPGLWGKRQAGPPGLWGKRDEDEDEDMDETNGDPLWGRSADAGP
PGLWGRKKRAASPQRDLYGIGLWGRNAALMTAEELDLSFKNEEQS*

Phymanthus crucifer

>WUCR01000590.1selectionselectiontranslationframe+1

MMARVKCQLVLLAIAFLLARCSGTVDKKDSRSNHLDEKKRESHEAQVVQETQAIKENSYLGSAEEESKRDDKK
RSAAPQQPGLWGK

>WUCR01000590.1selectionselection-1translationframe+1

RQSPGLWGRSADAQQPGLWGKRQSPGLWGRSADAQQPGLWGKRQSPGLWGRSAEPGQPGLWGKRDIPGLWGGK
RQSPGLWGRSAEPGQPGLWGKRDIPGLWGGKRQSPGLWGRSAEPLQPGLWGKRQNPGLWGRSAEPRQPGLWGK
RQSPGLWGRSAEPQQPGLWGKRQSPGLWGRSVF*

Scolanthus callimorphus

>GGGE01109457.1 TSA: SCOLANTHUS CALLIMORPHUS
TRINITY_R1_DN173494_C0_G1_I1, TRANSCRIBED RNA SEQUENCE

LWGKRQATSPGLWGKRQAGSTGLWGKRQAARPGLWGKRQATSTGLWGKRQAGSPGLWGKRQAGSTG

>GGGE01109458.1 TSA: SCOLANTHUS CALLIMORPHUS
TRINITY_R1_DN173494_C0_G1_I2, TRANSCRIBED RNA SEQUENCE

GKRQAGSTGLWGKRQAASPGLWGKRQAGSTGLWGKRQAARPGLWGKRQATSTGLWGKRQAGSPGLWGKRQAGS
TG

>GGGE01109459.1 TSA: SCOLANTHUS CALLIMORPHUS
TRINITY_R1_DN173494_C0_G2_I1, TRANSCRIBED RNA SEQUENCE

GKRQAGSTGLWGKRQAGSTGLWGKRQAGSPGLWGKRQAASPGLWGKRQARRPGLWDKRVVDEDEQELRRSAGP
PGLWGRTIEKDARTQITTPADVYGLGLWGRDEGLVAAEPDQRIAHLELDLQIEDKSKV

>GGGE01109460.1 TSA: SCOLANTHUS CALLIMORPHUS
TRINITY_R1_DN173494_C0_G3_I1, TRANSCRIBED RNA SEQUENCE

MAPLGRTVIAVLLLLTLCSAKSENQEKQTAVQEGKLTSQKREAKSPGLWGKRDTDAKQPGLWGKRQAPGLWGR
SPDAAHTGLWGKRKANSPEIDEKRSSDASSPGLWQKRSSRPAQPGLWGREAASPGLWGKRKAGSTGLWGKRQA
GSTGLWGKRQATSPGLWGKREAGSTGLWGKRQAARPGLWGKRQATSPGLWGKRQAGSTGLWGKRQAGSTGLWG
KRQATSPGLWGK

Exaiptasia diaphana

>NW_018384632.1 EXAIPTASIA PALLIDA ISOLATE CC7 UNPLACED GENOMIC SCAFFOLD,
AIPTASIA GENOME 1.1 SCAFFOLD150, WHOLE GENOME SHOTGUN SEQUENCE

MALKGQLCVILTTLLLIQCQGKSTKKENIEQHKAVQTSGAERTGSIAGELSEISEERREAEPPQFGLWGKRQV
ESPIEDPQFFDKKANSFGLWGKRGNGVGLWGRSADSWSKRQDSGLGLWGRSANPGNAVGLWGKRQRGGGRRGL
DAKRYANPGDGVGLWGKRQHDFGLWGRSAEPGNPVGLWGRVADKRDEQKRQKSIGLWGRSADPQKIGLWGR

Scleractinia (see Table 4, neuropeptide family 3)

Acropora millepora

>GHGU01049817.1 TSA: ACROPORA MILLEPORA COMP120237_C0_SEQ1, TRANSCRIBED RNA SEQUENCE

MPAVQIALLLMVVLVTPSLARHLEKTENDGADELSTKVEREELSESAMDSANDDNILEIRELKDIKGGRSPKT
LRLSQSLGTHIARQTGRESVDDSHKLVKELDESVEDGFGPPGLWGRREIRHGENEKSKQEDGEKLARLPGLWG
RETRQSPPGLWGRGISNDPPGLWGRGVKNGPPGLWGRNIISEVTENGKRRLPRMQKGEDA

Acropora digitifera

>NW_015441458.1selectionselectiontranslationframe+1

MPAVQIALLLMVVLVTPSLARHLEKTENDGADELSTKVEREELSESAMDSANDDNILEIRELKDIKGGRSPKT
LRLSQSLGTHIARQTGRESVDDSHKLVKELDESVEDGFGPPGLWGRREIRHGENEKSKQEDGEKLARLPGLWG
RETRQSPPGLWGRGISNDPPGLWGRGVKNGPPGLWGRNIISEVTENGKRRLPRMQKGEDA

Mantipora capitata

>GFRO01000152.1 TSA: MONTIPORA CAPITATA C135713_G1_I1 TRANSCRIBED RNA SEQUENCE

MPAYQIAILLLVALLTSSFARHLEKTEKDETGELPSKVEREEKSSEPIINTENDHTFIKNEESNAKRQDDIPV
EWGRSLVFLSLPQSLDTFQDARRTGLESPDYRLKQGQESDEPAKEGQFKPPGLWGRGLPYKNEEAKQENKEKH
RRLPGLWGREVMQSPLGFWGREIRQRPPGSLGKYRRPGLWGRSLGRKTPRNRERSIQNVQQNEDAQADLSYKE

Pocillopora damicornis

>XP_027060553.1 LWamide neuropeptides-like isoform X1 [Pocillopora damicornis]

MPAFKVTVFFLIFSAMCNARQFEAATDEDSAIGALHERKDARDETRKQRNSAEEETLNAEVDDMQRLSESHSN
KREDDLPRVWERSIKNENQVQKIDNDLPSSAEQADESEDREQDDSPSVLSARNMAKRRGIKDGPADLWGRGIE
NGPPGLWGRGLNNGPPGLWGRGLNNGPPGLWGRGLDNGPPGLWGRGLKNGPPGLWGREARGTSALGRRNANGP
PGLWGREIKNGPPGLWGRGLRSGAVGLWERELKNGPPGLWGRGLRNGPPGLWGREIEKVSKSSNRELSDVKSK
EEDGNEAKEDIQEMK

Stylophora pistillata

>GARY01038459.1selectionselectionrevtranslationframe+1

MPAFKVIVFFLVLLAMCNARRSEFTDEDSAVSELHQRGDALDETRKQQNSVEEETLNTEADDIQHLPESHSNK
RKDDFPGMLERSTEYQNQAQKIGNDLPFGPEQEDESEDHYQDESPSVFSARNKAERREIIYGPGLWDRGLDNR
PPGLWGRGLSNKPPGLWGRGLENRPPGLWGRGLQNRPPGLWGREVRGSSALGLRNANKPPGLWGRGLRSSPLG
LWGRELNNKPPGLWGRGLQNRPPGLWGREIEKVSKASKRELIGVKSTEEDGNESKEDIQELK*

Porites rus

>Porites rus isolate 14846/IV/SATS-LN/2007 genome assembly, contig: sscaffold00176, whole genome shotgun sequence

MSSLKAVFLSVLVLFLSTSCALQLEAKNDEKTGELSYNAQEKRDDETKTDSSNNLQRDSASGINRGEGMVTLA
DLAEIKQRSFMGRKAQPPGLWGRSLRNQPPGLWGRQATVEEEEEDSDDERKINSRIPGLWGRGLKNRTPRLWE
RSGEDEKNLREEHENDIFLAQNKPPGLWGRGTNDHEDFEDKSMRALSGPWERGLRNNRPGLWGRGIEGSHPGL
WGSNTRDDPSENWSRQLQPGLWGRALKNSPKELWRRNFFSSETDEDKDIDFNSK*

Orbicella faveolata

>NW_018149873.1 ORBICELLA FAVEOLATA ISOLATE FL UNPLACED GENOMIC SCAFFOLD, OFAV_DOV_V1 SC7JCM8_3106, WHOLE GENOME SHOTGUN SEQUENCE

MPTLKATIFLLLVLLPLCHARHFEGTDEDSADGDKLSLDMQEKREDEAKTEDLDSAKKDAESAGDSLVRRFLK
SSSDKRQDDMPGIWGRSIESKDGDEMHAQLVKLDQSEENEERRDEGAEPQPPGLWGREFENKLPKPAGVWERG
IENKQPPGLWGRDIQNKLPKPAGVWERGIEDKQPPGLWGREIENGPNGLWGQDAQNGPPGLWGREPQPPGLWG
REIENQPPGRALWGRGMSRAESWDDVDDKDEVIHDDK

Corallimorpharia (see Table 6, neuropeptide family 3)

Amplexidiscus fenestrafer

>scaffold_110selectionselectiontranslationframe+1

MPTLKATVLLVLVVLYSCDARHFRITDDSPNNNTPVREKKDDLEAPKSMQQHELGPAGQKRQEDLP
GVWGRSISIQPQSLENLEESSESLVEQETDSLSNKKRKEENVSPSLLERSFTKRESLDEGPLSFWG
RDLNDGFQDLWEREIRNGPPGLWGRQIGKTSPPGLSGREFKDSSPDLQGQGFENAPPGAYWGRELN
SGPPGVLWGREIDIASPGIWEREVEDGPPGLWGRDVEDGAPGLWGRDIKRRPPGMWGREIEDGPPG
LWGRDIGKPEMWARQVKRADERDEASYNNALETRSSEQVSD*

Corynactis australis

>GB|GELM01038038.1| TSA: CORYNACTIS AUSTRALIS COMP61921_C0_SEQ1 TRANSCRIBED RNA SEQUENCE

MPALKVTFFLLLAVLPLCEARHFRKTEDSKTNENLASGKKNEGTVPKTALNSDEDGQIMHADIRGPGIKRQDD
LPGMWGRSLSIQPHNLEPDEVDESSKSLVEQQVVSSSNKKREEEDGPPGLWGRSLNKEESLKDGPPGLWGREI
EHGPPGLWGRDLENAPPGPWGRELENGPPGLWGRELKNGPPGLWGRELKNGPPGLWGRELKNGPPGLWGRELK
NGPP

>GB|GELM01040281.1| TSA: CORYNACTIS AUSTRALIS COMP65537_C0_SEQ1 TRANSCRIBED RNA SEQUENCE

EIESGPPGLWGRELKNGPPGLWGREIESGPPGLWGRELKNGPPGLWGRELEGGPPGLWGRELEKGPPGLWGRQ
IEKGLPGLWGRDLDGGPPGLWGREFRSPVGSTEDSQRNTVETRSSEQDSDKNAEELSIEA

Discosoma sp.

>scaffold_1selectionselectiontranslationframe-1

MPALKATVLLVLVVLNSCDARHFRINDDSQNNDVLAIKKKDDSAAAPKSRQQDEGGPGQKRQDDLPGVWGRSI
SIQPQSLAENLKESSKRSVEQETDYLSNEKRQEENVSPSLRGRDLNDGSLDLWEREIRNGPPGLWGRQIGKMG
PPGLWGRELKDGSPGFENAAPGWAREINSGPPSHVLLDRERDIASPGIWEREVEDGPPGLWGRDVEDGPPGLW
GRDIVSGPPGLWGRDIESGPPGLWGRDIEDALPVMSGRKIKRSEERDQASPENAMETRSSEQD*

Ricordea yuma

>GB|GELN01016097.1| TSA: RICORDEA YUMA COMP18682_C0_SEQ1 TRANSCRIBED RNA SEQUENCE

MSSVKVMFLLLVVLLSFCEARHLRTENFESNAKKDEVNAQADTIEQQQRRQDDLPGVWGRGLFEENGDESSNR
VVDEELDAFSSNNKREKNGGPPGLWGRSFSAKDTLNEGPLGLWGRDLKGGGKGLWGRELKNGPPGLWGREIGK
SGPPGLWGRGIESGPPGLWGREVQGPPAGLWGRDAEGMNTDEHGEALEKNVMETRSIDDDAEGDVD

Protopalythoa variabilis

>GCVI01021756.1 TSA: PROTOPALYTHOA VARIABILIS CL11644.CONTIG1 TRANSCRIBED RNA SEQUENCE

MRLISLFLLIFFLSMSKARPSDNKESSKTANIATQKREIELPLKRDFQGAHGLWGRSAKDPVRERDLQNLHGL
WGRSTQDINGLWGRTLAKQGGTGLWGRSLQHRGLWGRTVREDAGGVHGLWGRSMKGSELEKVLRSLNPTRIGM
WGRRSIPTYEGIWGRSFMKNANKAEEKENEAKR

Zoantharia (see Table 6, neuropeptide family 3)

Zoanthus sp.

>GGTW01158323.1 TSA: ZOANTHUS SP. QL-2018 UNIGENE106425 TRANSCRIBED RNA SEQUENCE

MRTGMYFLLVIFLALSGARLTTSSKLSTQKREIGFREFDNFQKAAKNVNSPEKVLKRSTTSEDAQFNGLWGRS LQTAGLWGRNLRAGLSGKPLPQNMLTTRNFPGGLHRRGPRDGRSMQATNGLWGRSLFGQNVAELWGKSMNVKQ PVDRRTIKEDEGGVHGLWGRSLKERDSRDSTPTLYVPGMWGKRDETENSNYKKRNVNEAKMA

Ceriantharia (see Table 6, neuropeptide family 3)

Pachycerianthus borealis

>HAGY01045747.1 TSA: Pachycerianthus borealis, contig TRINITY_DN275_c1_g1_i1, transcribed RNA sequence

MTQPLVLSLLSIAVFFYCASALPPPDQRVDSTTDNDFFWRSGYENNLKQTPDQWLQTRRHLKPPTNFGLWGRS IERTENDPNTYSQKDVVTRQARPPPGVGLWGKRDLWQSKGKPDTPGRNEIRTWGN

>HAGY01000524.1 TSA: Pachycerianthus borealis, contig TRINITY_DN8540_c0_g2_i1, transcribed RNA sequence

MFISTCLIVLAMTLSMTHSKAIDENYLEDGLSHETINWPEGWESEEKNAEEVPMYSRPHRELAASRPPFGLWG RKRSAGKTKSPPKNPMAGLWGKRSLEFQHKK
