# Supplementary material for: A comparative genomics study of neuropeptide genes in the cnidarian subclasses Hexacorallia and Ceriantharia
Source: BMC Genomics. 2020 Sep 29;21:666. doi: 10.1186/s12864-020-06945-9 (PMC7523074; doi:10.1186/s12864-020-06945-9)
Supplement: Supplementary file 4 — Additional file 4. Partial or complete amino acid sequences of the Antho-RPamide preprohormones or related preprohormones in species belonging to the orders Actiniaria, Scleractinia, Corallimorpharia, or Zoantharia (belonging to the subclass Hexacorallia). [file 12864_2020_6945_MOESM4_ESM.pdf]

Additional file 4. Partial or complete amino acid sequences of the Antho-RPamide preprohormones or related preprohormones in species belonging to the orders Actiniaria, Scleractinia, Corallimorpharia, or Zoantharia (all part of the subclass Hexacorallia), or Spirularia (belonging to the subclass Ceriantharia). For some species more than one preprohormone fragment was identified. Signal sequences are underlined. An asterisk indicates a stop codon. Neuropeptide sequences are highlighted in yellow; C-terminal processing sites are highlighted in green. The C-terminal Gly residues that are converted into C-terminal amide groups are highlighted in red.

Actiniaria (see Table 2, neuropeptide family 4)

Anthopleura elegantissima

This is the Antho-RPamide-1 preprohormone cloned by us in 1996. It contains one copy of the isolated and sequenced neuropeptide Antho-RPamide-1 plus another RPamide peptide. See reference [10] and

https://www.jstor.org/stable/3883730?origin=JSTOR-pdf&seq=1#metadata_info_tab_contents

>Antho-RPamide preprohormone

MASKLLLCMALLVVVFVLSVESRQKAGVVQADQYRPRPGKKQYDGPEGDYEDLPPGPLPRPGRKRFFEDY*

Below are the three RPamide preprohormones retrieved from the transcriptome database:

>GBXJ01051472.1 TSA: Anthopleura elegantissima comp52214_c0_seq2 transcribed RNA sequence

MASKLLLCMALLVVVFVLSVESRQKAGVVQADEYRPRPGKKQYDGPEGDYEDLPPGPLPRPGRKRFFEENY

>GBXJ01055841.1 TSA: ANTHOPLEURA ELEGANTISSIMA COMP54556_C0_SEQ1 TRANSCRIBED RNA SEQUENCE

MACPRKIGLGLLCLFSVLIQGCFCEENENLVPNEHSVPEKLEHGEKGTVQYNVDTVKLTGSKHVKMDDKIEKP
PWPPRPGRRNTIPLKAKKALPTIIRPGRSIIESLMSKAKRNYQVKLYRPGRDDVPKGPPKLYRPGREDVPEGP
QNFHLRPGRDAMPQTLLRPGRGMAGPPSLFRPGREDVPQNFHLRPGREDVPNGPPSLFRPGREDVPQKLLRPG
RDEIPEQFNNVRAGRRSLGYNMPWTYEGSKVNSNVLHNSHTFRQKALEETAKRNYQDSEQEMSDPNSLQDEQQ
DF

>GBXJ01041884.1 TSA: Anthopleura elegantissima comp45991_c0_seq1 transcribed RNA sequence

MASLNIFLFSALLALLVVTCYGFRLEKDLESEYEGNSKEYQDAQKAIPIRPGKREYPLYRMLEDPQYVTIPVR
PGKRVVRARSNDASPQWPDVYTPYRPGRK*

Anemonia viridis

>GGLT01139692.1 TSA: Anemonia viridis TR91925:c1-g1-i1 transcribed RNA sequence

PEGPQNLLRPGRGDVPEGPQNLLRPGRGDVPEGPQNLIRPGRDAIPQGLFRPGREMAGPPSLFRPGREDVPQN
FQFRPGRDEIETGPPSLFRPGREDVPQRLLRPGRDEIPDQYHSVRAGRRSLGYNMPWTYEGSTVNKNAQHHSH
TFRQKALEETTKRNYQDNEQEMSDPNSFDFGECRSEWPF

>GGLT01105026.1 TSA: Anemonia viridis TR68509:c5-g1-i1 transcribed RNA sequence

MACPRKTGLGILCLFAILIQGCFCEEGENLVPNEQGSVPEKLVHGEKGTVQYKVNTVKMTGNEHVKMDENKNQ
RPPWPPRPGRRNIIPLSLRAKKTLPTVIRPGRSVIESLTSKAKRAYQDRLIRPGRDNVPKGPQYLYRPGREDV
PEGPQFLLRPGRDVPERPQTLLRPGRGDVPEGPQNLLRPGRGDVPEGPQNLLRPGRGDVPEGPQNLLRPGRG

Nematostella vectensis

>HADP01175575.1 TSA: Nematostella vectensis, contig TR68193|c5_g1_i2, transcribed RNA sequence

MYSCMGISLLLILCFKGSYGEESIDLEPGVDKVPEKVEHGEEGSAKYNINTVTLTGNKATGNKVTNDDKRPPW
PPRPGKRTFRPQESADTPSIFRPGRSVHEDQLLFRPGRANLHKRQGLMFRPGRREDVPKPQIFRPGRREDIPS
DDDQLMFRPGRNEEQLFRPGRRSDVGDEQLLFRPGRRSDLEEDQLIFRPGRRSDVAEEQLFRPGRRSDVPEAF
LDQWTSVRPGRGGYRMPWTYTGNSVNSHVSQKSHTFRQKAVEQEKKKREV

Phymanthus crucifer

>WUCR01000061.1selectionselectiontranslationframe+1

MACPRKTGLGLLCLLAVFIQGCLCEEDDNLIPNEHSVPEKVEHGEKGTVHYNVETVKLSGNKHTKMDDKTKRP
PWPPRPGRRNIISFKAKKTHPTVIRPGRSILESLKTKAKRSYQNNLLFRPGREDVQKDPPKFLRSGREDVPAG
PQFDWRTERDEVPQLQPLFRPGREMAGPLQELFRPGREDVPQSPLFRPGRDHVPNGSPPLFRPGREDVPQTLL
RPVRDNIPEQYHTVRAGRRSLGYNMPWTYVEGTVNSNLRHNTHTFRQKALEETKRNFQDESEQEMSDPNSLQE
EQQDF*

Scolanthus callimorphus

>GGGE01101217.1 TSA: SCOLANTHUS CALLIMORPHUS
TRINITY_R1_DN170058_C0_G2_I1, TRANSCRIBED RNA SEQUENCE

MTRNLNMTWAFCLVFTLWVQGTLCENKEQTIELSQDFVPEKIEQGSEGSINYKQNTIKLKGTQHKHWPPKEDT
GPRPPWPPRPGKKRFLRPQLPNKLIFRPGRSVHAEEHVFRPGRQMLRPGRQFLFRPGRSMSKRQGIILRPGRR
EDIAEDQSVFRPGRREAKDQFIFRPGRRDDIPEAFLDQFTSVRPGRAGYKMPWTYTGKSLNSHVSEKSHTFRQ
KALAEHKRTLEKSN

Exaiptasia diaphana

>TSA: Aiptasia pallida Loc_3802_Tr_2 mRNA sequence

MAVSTRLVVFVLGILIQGCFVFSEDNIKPDVDRIPEKIEHGEKGKSEYKVNSIILTGNQHTQVDNNDEDMRPP
SWPPRPGRRSFISRIKSRSSGPTIMRPGRELNSEKSKRMSSPQFVLRPGRGIDQHLAGDERDDVPQHPNILRP
GRDKIERPPSLFRPGREDVPQISLLRPGREIKTRLSVLRPGREDVPQISIFRPGREVEGPPPLFRPGREDVPQ
HISLFRPGREMVRRQVRLFRPGRELVADQGGASTVVRPGRRNIHYNMPWTYVGNTVDAHTHRKTH

Scleractinia (see Table 4, neuropeptide family 4)

Acropora millepora

>GHGU01096698.1 TSA: ACROPORA MILLEPORA COMP116100_C0_SEQ1, TRANSCRIBED
RNA SEQUENCE

MRFLLVIFAIGFLVTLAYAGQRTRIRPGKKDETSDPPNDSITQEDTSAEEEEEGLSYWIPVDAASAINKIPGN
DARTNFPRPGRKRTFPKYADMQGFIRPGRKRRSLDD

Acropora digitifera

> NW_015442502.1_FGENESH
MKDPGNEVALSTCLISTFLADAAPAGQRTRIRPGKKDETSDPPNDSITQEDTSAEEEEEEGLSYWIPVDAASAI
NKIPGNDARTNFPRPGRKRTFPKYADMQGFIRPGRKRRSLDD

Pocillopora damicornis

>RCHS01002836.1_FGENESH
MRGLLLIVIIAVLATFTTGALENEKRQFTNWRPGKKDETHLLGNQLHDLQAPETVKRQNKKLWRPGKKDKIQT
LGYHNAQASSGSIPTLNDQKQNDLETQMTGLGGVSPPVANIPFPRPGRKRRSMAA

Stylophora pistillata

>GARY01002305.1selectionselectiontranslationframe+1

MRALALIVIFGVSATFAFGDDEDVNQKNPWRPGKKDETYWLRDQLPVLESVKRQKGMWKPGKKDETHLLGNQL
NDLQAPETVKRQHTVWKPGKKDETHWLGNHNAQASTGSMQILNDGKQNDPETQITGMGGVSPPAANIPFPRPG
RKRRSMTA*

Corallimorpharia (see Table 6, neuropeptide family 4)

Ricordea yuma

>GB|GELN01016094.1| TSA: RICORDEA YUMA COMP18678_C0_SEQ1 TRANSCRIBED RNA SEQUENCE

MRAFLAAFFLGLLAVVAKSDDDSDWLNSELVDDENSLSSEPNNWNLEDLPKRKKPFFMPRPGKRSFDYLTSYD
AINSASDKREPQLDWRPGKKDNTGLFQYQQDDKPPLRPPRPGKKSVTSELRGFDRSISGGGHWKFTRPGKKRA
IERL

Zoantharia (see Table 6, neuropeptide family 4)

Protopalythoa variabilis

>GCVI01008171.1 TSA: PROTOPALYTHOA VARIABILIS CL3648.CONTIG1 TRANSCRIBED RNA SEQUENCE

MVSTTKRDLLKPLKVLLIGLLVLETIQESSCVNHEEHLIKKIPSKLEHSESGSSHYKLATLKMNGVQHRVKSD
ERFGPPGFPDAWPPRPGRRSLVETDQWWGDVEPGSPRPGRRTLDEQRLHRPRPGRTFKPQNEDFNPFKTKTRP
GRNAEEDLPWEYTGSSLHKGESKESHTLKKIAPEKKRGFHAKQIEIQRDFRI

Zoanthus sp.

>GGTW01056127.1 TSA: ZOANTHUS SP. QL-2018 UNIGENE4189 TRANSCRIBED RNA SEQUENCE

MNFSALNNMAATKCFLLAIILTVVTTEGFAFKLRDEEGEAAVADNKPPFPRPGRRGEIEAMLENEREVADHPP
LPRPGRRREIEAMLDNKRKVADHPPLPRPGRRRGIEAMLENKRKGFSPSF
