# Supplementary material for: A comparative genomics study of neuropeptide genes in the cnidarian subclasses Hexacorallia and Ceriantharia
Source: BMC Genomics. 2020 Sep 29;21:666. doi: 10.1186/s12864-020-06945-9 (PMC7523074; doi:10.1186/s12864-020-06945-9)
Supplement: Supplementary file 5 — Additional file 5. Partial or complete amino acid sequences of the pQITRFamide preprohormones or related preprohormones in species belonging to the orders Actiniaria, Scleractinia, and Corallimorpharia (belonging to the subclass Hexacorallia). [file 12864_2020_6945_MOESM5_ESM.pdf]

Additional file 5. Partial or complete amino acid sequences of the pQITRFamide preprohormones or related preprohormones in species belonging to the orders Actiniaria, Scleractinia, Corallimorpharia, or Zoantharia (all part of the subclass Hexacorallia), or Spirularia (belonging to the subclass Ceriantharia). For some species more than one preprohormone fragment was identified. Signal sequences are underlined. An asterisk indicates a stop codon. Neuropeptide sequences are highlighted in yellow; C-terminal processing sites are highlighted in green. The C-terminal Gly residues that are converted into C-terminal amide groups are highlighted in red.

Actiniaria (see Table 2, neuropeptide family 5)

Anemonia viridis

>OCZR010246212.1 Anemonia viridis genome assembly, contig: scaffold246212_len2040_cov40, whole genome shotgun sequence

QVTKSTDHKNGQISPSTDHKIWQVTSSTDNKIWQTRFGRAPDPQFTRFGRSPHPQITRFGRSAHPQITRFGRS
PNPPITRIGRSAHPQITRFGRSASAHPQITRFGKSPHPQITRFGRSPHPQITRFGRSAHPQITRFGRSAHPQI
TKFGRSAHPQVTRFGRSAHPKITRFGRSGHPQFTRFSRSPHPQITRFSRSPNPSINTSTDHKIWQVTPSADHK
IWQVTQSTDHKIWQVTRSTVHKIWQVTPSTNNKIWQVTPSTVHKIWQVTPSIDNKIWQVTPSTDRTIWQVTPM
HISHDLTDHPVRKITSFGTSPHPQCKRFDWCYLTHCLILLRRTIILLDLFIHK

Nematostella vectensis

>scaffold_47

MTYKSLLHVFIFLLSTCSAYGRSTRVSLKTTVTGGITRQPIPLRSTGYPSGSAYHSASLPIVRFTRSASPQIT
RFRKSDASPQITRFGRSASPQKTRFGRSASPQITRFGRSASPQITRFGRSASPQITRFGRSASPQITRFGRSA
SPQITRFEVGRLITRFGSHFQ

>HADP01261925.1 TSA: Nematostella vectensis, contig TR89867|c0_g1_i1, transcribed RNA sequence

MPNTCLIHVSLIVILTLYGATKGRGTPASRNTIVSRGIITASSNTFKGGIHRSKAVKSSPRNGVSTIASSEIT
SVRKSLKLPHIKELIKFASQPVARFGRSASPQITRFGRSASPQITRFGRSAS

Phymanthus crucifer

>WUCR01007002.1selectionselectiontranslationframe-2

MNLFLTVLGILIVFTLGSLHSIQRDSAVSNCTVNTSMPRSSNHRMKRGVAVLFYTTIKDRRRRSSQDIKQPSY
TERIQDSNTEMNQQPRHPQERNISRKNIVITNKKQKTTAKKTISRTFIYSKIRTLKSRHQDTRRTYSSTYPKI
QRSTHPQITRFGRTTHPQITRFGRTTHPQITRFGRTTHPQITRFGRTTHPQITRFGRTTHPQIT

Scolanthus callimorphus

>GGGE01084743.1selectionrevtranslationframe+1

SASPQITRFGRSASPQITRFGRSASPQITRFGRSTSPQITRLGRSASPQITRFGRSASPQITRFGRSASPQIT
RFGRSASPQITRFGRSASPQITRFGRSTSPQITRLGRSASPQITRFGRSASP

Exaiptasia diaphana

>NW_018384751.1 EXAIPTASIA PALLIDA ISOLATE CC7 UNPLACED GENOMIC SCAFFOLD, AIPTASIA GENOME 1.1 SCAFFOLD1621, WHOLE GENOME SHOTGUN SEQUENCE

MKTTLLIRSCSAFLIFTQLCILSGAENNSEIDEKFGQSYIKQVKRLDRPKMKIFGRSIKPQVTAFERSIRPQV
TRFGRSITPQVTRFGLSIRSQVTRFGRYIRPQVTRFGRSIISQVTRFGRSIRLQVTRFGRSTRSQVTRFGRSI
KPRATRFGQSIRTQVTRFGRSIRSQVTRFVRSIKPLAKKFGRSIRTQVTRFRRSIRSQVTRFGRSIRLQVTRF
GRSIRSQVTRFARYVRPQVTRFGRSVKSQVKGFERSTRSQATRFGRSIRSQVTRFGRSIRSQVTRFGRSIRSQ
VTRFGRSTRPQVTRFGRSIISQVTRFGRSIRLQVTRFGRSTRPQVTRFGRSIISQVTRFGRSIRLQVKRFERS
TRSQVTRFGRYVRPQVTRFGRSIISQVTRFGRSIRPQVTRFGRSIRPQVTRFGRSIRPQVTRFGRSIRPQVTR
FGRSIRPQVTRFGRSIRPQVTRFGRSIRPQVTRFGRSIRPQVTRFGRSIRPQVTRFGRSIRPQVTRFGRSIRP
QVTRFGRSIRPQVTRFGRSIRPQVTRFGRSIRPQVTRFGRSIRPQVPSDYRSQDLDGPPDLKLQDSDDPSYLR
SQDLDDPSDYRSKDLNGPPDHRSQDLGGTSDLKSQDLDGPSYHRSQDLEGPSDLRSQDLEGPSDLRSQDLEGP
SDLRSQDLEGPSDLRSQDLEGPSDLRSQDLEGPSDLRSQDLEGPSDLRSQDLEGPSNLRSQDLEGPSDFRSHD
LDDPLNLELQDLDGPSDLRS

Scleractinia (see Table 4, neuropeptide family 5)

Acropora millepora

>QTZP01000245.1_selection_selection_rev_translation_frame_+1

MLLLIVLYSILPLLYGFLEKQSKAHLHILGRSVTPQSITRFGRSVTPQSITRFGRSVTPQSITRFGRSVTPQS
ITRFGRSVTPQSITRFGRSVTPQSITRFGRSVTPQSITRFGRSVTPQSITRFGRSVTPQSITRFGRSVTPQSI
TRFGRSVTPQSITRFGRSVTPQSITRFGRSVTPQSITRFGRSVTPQSITRFGRSVTPQSITRFGRSVTPQSIT
RFGRSVTPQSITRFGRSVTPQSITRFGRSVTPQSITRFGRYVTPQKKFQYI*

Acropora digitifera

>NW_015441139.1selectionselectiontranslationframe+1

MLLLIVLYSILPLLYGFLEKQSKAHLHILGRSVTPQSITRFGRSVTPQSITRFGRSVTPQSITRFGRSVTPQS
ITRFGRSVTPQSITRFGRSVTPQSITRFGRSVTPQSITRFGRSVTPQSITRFGRSVTPQSITRFGRSVTPQSI
TRFGRSVTPQSITRFGRSVTPQSITRFGRSVTPQSITRFGRSVTPQSITRFGRSVTPQSITRFGRSVTPQSIT
RFGRSVTPQSITRFGRSVTPQSITRFGRSVTPQSITRFGRSVTPQSITRFGRSVTPQSITRFGRSVTPQSITR
FGRSVTPQSITRFGRSVTPQSITRFGRSVTPQSITRFGRSVTPQSITRFGRSVTPQSITRFGRSVTPQSITRF
GRSVTPQSITRFGRSVTPQSITRFGRSVTPQSITKF*

Mantipora capitata

>RDEB01000310.1selectionselectionrevtranslationframe+1

MHLFWLIQVLILWRLLPGLYGFPNFLQSVTGIGRSITPQAVTRFGRSLRPQSVTRFGRSITPQSVTRFGRSIA
PHSVTRFGRSIAPQSVTRFGRSIAPQSVTRFGRSITPQSVTRFGRSITPQSVTRFGRSITPQSVTRFGRSITP
QSVTRFGRSITPQSVTRFGRSITPQSVTRFGRSIAPQSVTRFGRSITPQSVTRFGRSITPQSVTRFGRSIAPQ
SVTRFGRSIAPQSVTRFGRFITPQLVTRFGRSITPQSLTRFGRSIAPQSVKGLDDPSHHNQ*

Pocillopora damicornis

>RCHS01002970.1_selection_selection_translation_frame_+1

MKIVVVPIYQLILLWKLSLTIHGLKKDEPKISHGISQESKRSLQPQSMTRCGRSTDSNPPTTPWTRHGKSLRP
QSVTRFRRSADTKPIRGGNNYISSIIPQSIARFGGFADKNPISQWKRHGRSIRPQSVTKFGRSADTKPIHGGD
SYTRSIIPQSITRFGRSADKNPISQWTRDGRSIRSQSTKRFGLSAGTKTIHGETKYIRSIRPQSITRFGRSA

Stylophora pistillata

>NW_019217873.1_selection_selection_rev_translation_frame_+1

MKIILVPICLLMLQWNLSVMTHRLKKDEPKISHGVPQERMRSLQPQSITKFGRLTDGNPTTPWTRNGRSIRPQ
SITRFGRSADTKPIHGGKNYFRSRRPESVIPFGRSADKNPINQWTREVKSIRPQPITRFARSADSNPSYQWTR
YRRFLRPQSITRFGRSAKDNPSNQWTTFKRSLKPQSITRFGRSADGNPTNYWIRYRSPRPQSITRFRRSADGN
PTIQWKRYKISPRAQSITRFGRSADGNPTNQWERYRRSPRPQSITRFGRSADGNPTNHWIRYRRSPRPQSITR
FGRSADGNPTIQWKRYKISPRTQSITRFGRSADGNPTDYWIRYRRSPRPQSITRFGRSADGNPTIQWKRYKTS
PRAQSITRFGRSADGNPTNQWERYRRSPRPQSITRFGRSADGNPTNHWIRYRRSPRPQSITRFGRSADGNPTI
QWKRYKRSPRAQSITRFGRSADGNPTNQWERYRRSPRPQSITRFGRSADGNPTNHWIRYRRSPRPQSITRFGR
SADGNPTIQWKRYKRSPRAQSITRFGRSADGNPTNQWERYRRSPRPQSITRFGRSADGNPTNHWIRYRRSPRP
QSITRFGRSADGDPTNQWIRYRRSSRPHLIRRFGRSADTNPSNRWKRYKRFLRPQSITRFGRSADSNPSNQWK
RYRRSLKPQSTTRIGRAADSNPSNQWKRYRRFPRPRSISSFGRSADGNPTNQWKRYRRSPRPQSVTRFGRSAD
DNGTIQWKRYRRSLRPQLITRFRRSADSDPSDQWKTYRTSLGPQSITRFG*

Porites rus

>Porites rus isolate 14846/IV/SATS-LN/2007 genome assembly, contig: sscaffold00497, whole genome shotgun sequence

MFSVPFYRVLVLCRLWTLSYGVIPAENKLSETKVNPLQITKRNEVISRQIPRVMLFKQYPNRWERYRDATETS
PVKNLEKPFEPRALRFERPIRPRSVTILGLSTRQQSRKRHGRSIRPQLVTRFGRSIRPQLVRRFGRSIRPQIV
RRFGRSIRPRLVTRFGRSLRPQLVTRFRRSIGPQLVTRYGRSNI*

Corallimorpharia

Amplexidiscus fenestrafer

>scaffold_1selectionselectiontranslationframe-1

MNLINQSYLSYKILLLAQLLFISKTQGSKHTGKRYHLAANIPTESWESSSTPQNQFQQYERSMTPL
SLIKRFGRSVDMKRNQQWTRYGRSMIPPSLTRFGRSVDMKRNQQWTRYGRSMTPSQLTRFGRSVDM
KRDHQWTRYGRSMIPPSLTRFGRSVDIKGQQWTRYGRSMTPSQLTRFGRSVDTKRNQQWTRYGRSM
TPSQLTRFGRSVDIKRNQQWTRYGRSMTPPPLTRFGRSVDIKRNQQWTRYGRSMTPSQLTRFGRSV
DMKRQTPLSLTRFGRSVDTKSNQQWTRYGRSMIPPSIPRFGRSVDDIKRQTPRPRL*

Discosoma sp.

>scaffold_36selectionselectiontranslationframe+1

MNLMNQTFLSFKILLLVQLLYISKIQGSKRSADKRYHLAAYQPTQRSESSGIPQHHLQRYRRSTTTPPPLTRF
GRSVAIKRNQQWTRYGRSMTPQSLTRFERSVDTKRNQQWTRYGRSVDMKRNQQWTRYGRSMTPQSLTRFGRSV
DTKRNQQWTRYGRSVDMKRNQQWTRYGRSMTPQSLTRFGRSVDTKRNQQWTRYGRSVDMKRNQQWTRYGRSVD
MKRNQQWTRYGRSIIPPSLTRFRRSVDMKRNQQWTRYGRSVDMKRN*

>scaffold_36selectionselectiontranslationframe-1

MNLMNQTFLSFKILLLVQLLSISKTQGNKRTADKRYHLAAYNPTEKSESSGIPQHHLQRYRRSTTTPPPLTRF
GRSVDMKRNQQWTRYGRSVDMKRNQQWTRYGRSVDMKRNQQ*
