# Supplementary material for: A comparative genomics study of neuropeptide genes in the cnidarian subclasses Hexacorallia and Ceriantharia
Source: BMC Genomics. 2020 Sep 29;21:666. doi: 10.1186/s12864-020-06945-9 (PMC7523074; doi:10.1186/s12864-020-06945-9)
Supplement: Supplementary file 6 — Additional file 6. Partial or complete amino acid sequences of the FHIRamide preprohormones or related preprohormones in species belonging to the orders Actiniaria, Scleractinia, Corallimorpharia, or Zoantharia (belonging to the subclass Hexacorallia). [file 12864_2020_6945_MOESM6_ESM.pdf]

Additional file 6. Partial or complete amino acid sequences of the FHIRamide preprohormones in species belonging to the orders Actiniaria, Scleractinia, Corallimorpharia, or Zoantharia (all part of the subclass Hexacorallia). Signal sequences are underlined. An asterisk indicates a stop codon. Proposed neuropeptide sequences are highlighted in yellow; C-terminal processing sites are highlighted in green. The C-terminal Gly residues that are converted into C-terminal amide groups are highlighted in red.

Actiniaria (see Table 3, neuropeptide family number 6)

Anthopleura elegantissima

>GBXJ01068702.1 TSA: ANTHOPLEURA ELEGANTISSIMA COMP60383_C0_SEQ2 TRANSCRIBED RNA SEQUENCE

YGQPAFFHIRGKREENPPYEEILEPAFFHIRGKRVAKQPPFLGGPAYFHIRGKRDVGPPYIDLTEPAFFHIRG
KRVAKQPPFLGGPAYFHIRGKREEYPPLVDLTEPAFFHIRGKRESSQE

Anemonia viridis

>Anemonia viridis genome assembly, contig: scaffold138930_len4308_cov81, whole genome shotgun sequence

MRFTLHVLSFALLIAVVTATNEKKEAKFPPNLGQPAFFHIRGKREESRPPIVDLTEPAFFHIRGKRVAKQPPF
LGESAFFHIRGKREENPPYEELLEPAFFHIRGKRVAKQPPFLGGPAYFHIRGKRDVGPPYVDLTEPAFFHIRG
KRVAKQPPFLGGPAYFHIRGKREEYPPLVDLTEPAFFHIRGKRVSIIPNNIQEK*

Nematostella vectensis

>HADP01054819.1 TSA: Nematostella vectensis, contig TR35242|c0_g1_i1, transcribed RNA sequence

MRLYLFVPVFALVLAVEGASDEKRDSKQPPIDLSPAAYFHIRGKRTHNAPPLDLSGPAYFHIRGKRTAKQPPY
LDLGEPSFFHIRGKRTEGPPYIDLTEPSFFHIRGKRSSEQPPLDLGPAYFHIRGKRTKNPPIDLGPAYFHIRG
KRLSGEQPPYLDLTPAYFHIRGKRTQQPPMIDLSEPAFFHIRGRRAVEQPPYLDLTPSYFHIRGKRTEYPPFL
ELGQPSYFHIRGRRAEKTTKD

Phymanthus crucifer

>WUCR01017082.1selectionselectiontranslationframe+1

MRISLVHGLSFVVLIAVVMATNEKKEAKAPPIIDLGQPAYFHIRGKRDENPPFEEILEPAFFHIRGKRVAKQPPFLGQPAYFHIRGKREENPPFEEILEPAFFHIRGKRVAKQPPFLGQPAYFHIRGKREENPPFEEILEPAFFHIRGKRVAKQPPLLGGPAYFHIRGKRDVSPPYVDLTEPAFFHIRGKRVAKQPPFLGGPAYFHIRGKRVSLSHTHANQMNILPTLFPNSLGSLKNCLKSRVTFPASRGSRSKEKGSLCRSRPFAMSRMVTRQPLSKLITSR*

Exaiptasia diaphana

>TSA: Aiptasia pallida Loc_7399_Tr_1 mRNA sequence

MKNVLSLLSLAMMLSIVIATEEKKDAKEQPPFVDLTQPAFYHIRGKRVARQPPFVDLTQPAFYHIRGKRVAKQPPFAVDLTQPAFYHIRGKRQFPPNVDLTAPAYYHIRGKRVAQQPPFVDLTQPAYYHIRGKRDN*

Scleractinia (see Table 5, neuropeptide family 6)

Acropora millepora

>GHGH01030046.1 TSA: ACROPORA MILLEPORA COMP71360_C0_SEQ1, TRANSCRIBED RNA SEQUENCE

MFLRSISFLFFAVCLVKCETKKRQEDPPLQSVLNTEYFGHVRAGRRDSRDVNPPLKEALNNAYFGHFRGRRKSGFDSDKDVLEALENQYFGNIRGKRQTSDKDLLEAFKTAYFGNIRGKRHEGNERRQRGLQDYSKSLE

Mantipora capitata

>GFRO01000833.1 TSA: MONTIPORA CAPITATA C199810_G1_I2 TRANSCRIBED RNA SEQUENCE

MFLRIIVFFFVVYLVSCETDRKHREEDPPLQEALNTKYSAHVRAGKRGSDNDYPPLKEALSKTHFGHFRGKRKGGLESEEGLLEALKNVYFGNIRGKRQMSDKDLLPALKSEYFGNIRGRREKDNELRGKESQDTSRHSE

Pocillopora damicornis

>XP_027043620.1 uncharacterized protein LOC113671573 isoform X1 [Pocillopora damicornis]

MMFLRLAVLFCVVCLASSEKEKNSEDPPGLLQALNSKYFGHIRGKRTGKDDNPPLEQALNNAYFGHIRGKRTQSDDYPPLEEALNSAYFGHIRGKRTGGWGSNKDLLEALNNAYMGHIRGKRQQETMDKSV

Stylophora pistillata

>GARY01013793.1selectionselectionrevtranslationframe+1

MMFLRLAVLFCVVCLASSETEKKSEDPPGLLQALNSKYFGHIRGKRTDKDDSPPLEQALNNAYFGHIRGKRTQ
SDNHPPLEQALNKAYFGHIRGKRTVITSL*

Porites rus

>OKRP01000467.1_FGENESH
MFLRIFLFFFLMVLTSCSETKKDTQDSPPDLLQALRSKYLGHIRGKRTDSADSDPPALKEALGNAYFGHIRGK
RTGGWESNKDLLEVLNNAYSGHIRGKRQQDTSKNSQ

Orbicella faveolata

>MPSW01001564.1_FGENESH
MLLRLVILFSMVCLASCETKTNSEDPPLLEALNAKYFGHIRGKRMESEDYPPGLKEALSNAYYGHIRGKRTGG
WASNQDLLEALNNAYMGHIRGKREQVHTEHLLSVKFLSESCFSP

Callimorpharia (see Table 7, neuropeptide family 6)

Amplexidiscus fenestrafer

>scaffold_13selectionselectiontranslationframe+1

MFLRLASLCFVVCVVNCKTKETSDDPPWAEALNAKYFGHIRGKRTVCKPFQFSFSPSL

Corynactis australis

>GB|GELM01044721.1| TSA: CORYNACTIS AUSTRALIS COMP71691_C0_SEQ1
TRANSCRIBED RNA SEQUENCE

MLLKLATVFLVVCFANCKPKQTAEADPPWAEAVNAKYFGHIRGRTTDVEDSVPPYEEALNRAYFGHIRGKRTE
SQDSPPYEEALNRAYFGHIRGKRTLEGWGINPAVLEALNNQYFGHIRGKRTGGWGSNADLLEALNNEYMGHIR
GKRTQNDKKSE

Discosoma sp.

>scaffold_34selectionselectiontranslationframe+1

MFLRLATLCFVVCVVNCKTKDTADDPPWAEALNAKYFGHIRGKRTESEDYPPYEEALNRAYFGHIRGKRTGGW
ESNADLLQALNNEYMGHIRGKREQVNIATITA

Ricordea yuma

>GB|GELN01022761.1| TSA: RICORDEA YUMA COMP41129_C0_SEQ1 TRANSCRIBED RNA SEQUENCE

MFFKLAILFSVVSLANCKTKKTAEDPPWEEALNSKFFGHIRGKRTDSQDIPPYGEALNNEYFGHIRGKRTLED
WRINPEVLQALNDQFFGHIRGKRWGGWDRNGDLQNALNNAYMGHIRGKRDQTQSTKKSE

Zoantharia (see Table 7, neuropeptide family 6)

Zoanthus sp.

>GGTW01139254.1 TSA: ZOANTHUS SP. QL-2018 UNIGENE87347 TRANSCRIBED RNA SEQUENCE

KQEIPLASLMKGNVAYGHIRGKKQEIPLQSILNGGVAYGHIRGKRSHLPPYDNRVAFGHIRGKRQNFGPGWLN
ELQMLGHIRGRKRQFLKPNHFMGSGVAYGHIRGKRQLPFSQLMNSGVSFGHIRGRRQFPIDGLDGISYLGHIR
GKRTEKSI
