# Supplementary material for: A comparative genomics study of neuropeptide genes in the cnidarian subclasses Hexacorallia and Ceriantharia
Source: BMC Genomics. 2020 Sep 29;21:666. doi: 10.1186/s12864-020-06945-9 (PMC7523074; doi:10.1186/s12864-020-06945-9)
Supplement: Supplementary file 7 — Additional file 7. Amino acid sequences of the Antho-RNamide preprohormones in species belonging to the orders Actiniaria and Spirularia [file 12864_2020_6945_MOESM7_ESM.pdf]

Additional file 7. Amino acid sequences of the Antho-RNamide preprohormones in species belonging to the order Actiniaria. Other hexacorallian species do not express these preprohormones. However, C. borealis (subclass Ceriantharia) has one gene, coding for a Antho-RNamide preprohormone. For some actiniarian species more than one preprohormone was identified. Signal sequences are underlined. An asterisk indicates a stop codon. Neuropeptide sequences are highlighted in yellow; C-terminal processing sites are highlighted in green. The N-terminal Phe residues of the immature peptides (highlighted in blue) are converted into N-terminal phenyllactyl residues. The C-terminal Gly residues that are converted into C-terminal amide groups are highlighted in red.

Actiniaria (see Table 3, neuropeptide family 7)

Anthopleura elegantissima

>GBXJ01041885.1 TSA: Anthopleura elegantissima comp45992_c0_seq1 transcribed RNA sequence

MASSKTLVVLLAVFMLCGVVFQCTDAFLRNGRDEIMARKRAEMGNPPYEEMYQYDEANMAKARRAKRSKQ*

>GBXJ01137864.1 TSA: Anthopleura elegantissima comp71182_c0_seq1

transcribed RNA sequence

MSKGTVLVLLVVLIAGVMVQTGDAFLRNGRNNIPLAREDGLPRDFYEQYYERFPQDSRFKKRRFSKDMA*

>GBXJ01087037.1 TSA: Anthopleura elegantissima comp65659_c2_seq2 transcribed RNA sequence

Anemonia viridis

>GHCD01132920.1selectionselectionrevtranslationframe+1

MASSKTLVVLLAVFMLCGVVFQGTDAFLRNGRDEIMARKRAEMGYPPYEEMYQYDEANMAKARREAKRSKQ*

>GHCD01074248.1selectionselectiontranslationframe+1

MSKGTVLVLLVVLIAGVMVQTGDAFLRNGRNNIPLAREDGLPRDFVRAILSRLPPGLHDSRNDDLAKTWHE*

>GHCD01038475.1selectionselectionrevtranslationframe+1

ILIFVLCLFQGTDAFLRNGRDEIMEVALAPSLFSIHCHPSPQISEIYDYKGLSEVRLD*

Nematostella vectensis

>HADO01000428.1selectionselectionrevtranslationframe+1

MASSRTLVAFLAVFVLCGMLIQTSDAFLRNGRDEIYAKKRAEMGYPPRWEEYENQLYQRRDEDAKRSKQ*

>HADP01211238.1selectionselectiontranslationframe+1

MKRSLVVVLVVAIAAAMFVETEGFLRNGREQIPSKLERDAAELEDFYNALYAQQRANPGYDRFRRERKANA*

Phymanthus crucifer

>WUCR01005850.1selectionselectiontranslationframe-1

MASSKTLVALLAVFMLCGVVFQCTDAFLRNGRDEIFARKRAEMGLPPYGEMYEYDEANMAKARRGKKTSASYF
DR*

>WUCR01021523.1selectionselectiontranslationframe-1

MSKGTVLVLLVVLIAGVMVQTGDAFLRNGRNNIPLDREEGFPRDFYQQYYQGFPQDSRFKKRKYDNLLYG*

Scolanthus callimorphus

>GGGE01317346.1selectiontranslationframe-1

MASTRTLVAVLAVFVLCGVFIQTSDAFLRNGRDMIFARKRAEMGNPPVDYELPEYDAAEMMKQRREAAKRSKQ
*

>GGGE01349750.1selectiontranslationframe-2

MKTSVVVVLVVLVACSMLIQTEAFLRNGKKRSFERARDAADLEEYLDAIYAEGRGYDIPERFRRGRKWQA*

Exaiptasia diaphana

>TSA: Aiptasia pallida Loc_16101_Tr_1 mRNA sequence

MASSKTLVALLVVFMLCGVVFQSTNAFLRNGRDEIYARKRGEMGVPPYMEYDAADLARARREFAKRTKQ*

>TSA: Aiptasia pallida Loc_10681_Tr_1 mRNA sequence

MNKSVIVVMLAVLVASVMVQTGDAFLRNGRDSIPAKRENALADEFFNEYLRERFPVDDRFKKRRFAKDMA*

Ceriantharia (see Table 7, neuropeptide family 7)

Pachycerianthus borealis

>TSA: Pachycerianthus borealis, contig TRINITY_DN3630_c0_g1_i1, transcribed RNA sequence

MASAKILVALLVVALFASVFVSETEAFLRNGRDSIPFKRRQTQRFYYPEDLEEFQRPQENKLDTEYLKQK*
