# Supplementary material for: A comparative genomics study of neuropeptide genes in the cnidarian subclasses Hexacorallia and Ceriantharia
Source: BMC Genomics. 2020 Sep 29;21:666. doi: 10.1186/s12864-020-06945-9 (PMC7523074; doi:10.1186/s12864-020-06945-9)
Supplement: Supplementary file 8 — Additional file 8. Amino acid sequences of the Antho-RIamide or related preprohormones in species belonging to the order Actiniaria. [file 12864_2020_6945_MOESM8_ESM.pdf]

Additional file 8. Amino acid sequences of the Antho-RIamide or related preprohormones in species belonging to the order Actiniaria. Other hexacorallian or ceriantharian species do not express these preprohormones. Signal sequences are underlined. An asterisk indicates a stop codon. Neuropeptide sequences are highlighted in yellow; C-terminal processing sites are highlighted in green. The N-terminal Phe residues of the immature peptides (highlighted in blue) are converted into N-terminal phenyllactyl residues. The C-terminal Gly residues that are converted into C-terminal amide groups are highlighted in red.

Actiniaria (see Table 3, neuropeptide family 8)

Anthopleura elegantissima

>GBXJ01087037.1 TSA: Anthopleura elegantissima comp65659_c2_seq2 transcribed RNA sequence

MSPMLKNLAILAIFVILITPRPSSGFYRIGREFETRPGTQGNTEGAIDSPDGFGADDLMNYRLAALRAKRLMA
KKQENDKRSLQ*

Anemonia viridis

>GHCD01115296.1selectionselectiontranslationframe+1

MSPMLKNLAILAIFVILITPRPSSGFYRIGREFETRPGTQDNTEGAIDSMDGLGADDLFNYRLAALRAKRLMA
KKQENDKSLQ*

Nematostella vectensis

>HADO01002733.1selectionselectionrevtranslationframe+1

MSPTMKYVLVAVLFCALVAPRFTHGFYRVGRELEKRREAMGGQDPLVQLAQGPDSDDLWRARMAYLRARRTEA
KKTEQR*

Phymanthus crucifer

>WUCR01005850.1selectionselectiontranslationframe+1-2

MSPMLKNLAVLAILVILITPRLSSGFYRIGREFETRPGMQRTSEEAIDSPDGMGAEDLMNYRLAALRAKRLMA
KKKENDKRSLQ*

Aiptasia diaphana

>TSA: Aiptasia pallida Loc_12743_Tr_1 mRNA sequence

MTSVVKTLAFLAILVILIVPHPSSGFYKIGRREVPRQNGEVKALEGSAREADFDDWLRNIMSLREARRMEKIA
KMNGNQ*
