# Supplementary material for: A comparative genomics study of neuropeptide genes in the cnidarian subclasses Hexacorallia and Ceriantharia
Source: BMC Genomics. 2020 Sep 29;21:666. doi: 10.1186/s12864-020-06945-9 (PMC7523074; doi:10.1186/s12864-020-06945-9)
Supplement: Supplementary file 9 — Additional file 9. Amino acid sequences of the Antho-KAamide or related preprohormones in species belonging to the order Actiniaria. [file 12864_2020_6945_MOESM9_ESM.pdf]

Additional file 9. Amino acid sequences of the Antho-KAamide or related preprohormones in species belonging to the order Actiniaria. Other hexacorallian or ceriantharian species do not express these preprohormones. Signal sequences are underlined. An asterisk indicates a stop codon. Neuropeptide sequences are highlighted in yellow; C-terminal processing sites are highlighted in green. The N-terminal Phe residues of the immature peptides (highlighted in blue) are converted into N-terminal phenyllactyl residues. The C-terminal Gly residues that are converted into C-terminal amide groups are highlighted in red.

Actiniaria (see Table 3, neuropeptide family 9)

Anthopleura elegantissima

>GBXJ01026192.1 TSA: Anthopleura elegantissima comp33156_c0_seq1 transcribed RNA sequence

MNRVALVLLLVCVSAVLMPSDVNAFFKAGRELREREAARQYGNEPQYGAADDRELWQALMNARNSQQKRNVAN
KQ*

Anemonia viridis

MNRVALVLLLVCVSAVLMPSDVDAFFKAGRELREREAARQYGSEPQYGAADDRELWEALMNARNSQQKRKVAN
KQ*

Nematostella vectensis

>HADO01001257.1selectionselectionrevtranslationframe+1

MNRLALVLLCMFLTAVLMPNRVQGFFKAGRELKEKSADSNERRQFNEGSVAEEEARKEFLRALLRSAERRSKT
DDYQ*

Exaiptasia diaphana

>TSA: Aiptasia diaphana Loc_26956_Tr_1 mRNA sequence

MNRLALVILFVCLSAVLINNQVDAFFKAGRSLHDRQAARQVDNAMEGEIGAADAKELWEEFVKARSAERKRAM
QDRQ*
