# Supplementary material for: A comparative genomics study of neuropeptide genes in the cnidarian subclasses Hexacorallia and Ceriantharia
Source: BMC Genomics. 2020 Sep 29;21:666. doi: 10.1186/s12864-020-06945-9 (PMC7523074; doi:10.1186/s12864-020-06945-9)
Supplement: Supplementary file 11 — Additional file 11. Scan of two pages from the Ph.D. thesis by: Carstensen K. Struktur, Wirkungsweise und Biosynthese von Antho-RPamiden, einer neuen Neuropeptidfamilie aus der Seeanemone Anthopleura elegantissima BRANDT. 1993; Ph.D. thesis, Faculty of Biology, University of Hamburg (written in German). [file 12864_2020_6945_MOESM11_ESM.pdf]

Struktur, Wirkungsweise und Biosynthese von Antho-RPamiden, einer neuen Neuropeptidfamilie aus der Seeanemone *Anthopleura elegantissima* BRANDT

DISSERTATION
zur Erlangung des Doktorgrades
des Fachbereichs Biologie
der Universität Hamburg

vorgelegt von
KLAUS CARSTENSEN

Hamburg 1993

Antiseren gegen die schon bekannten Peptide markiert wurden. Diese ersten Ergebnisse zeigten, daß es in Seeanemonen vermutlich Neuropetide gibt, die RPamid als carboxyterminale Struktur aufweisen.

a

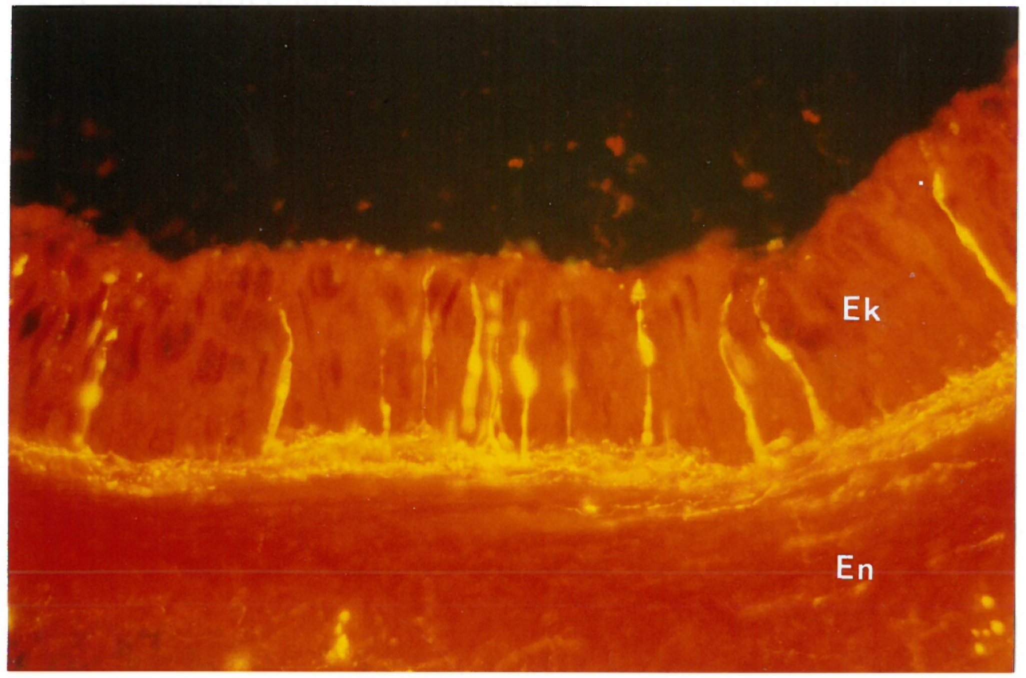

b

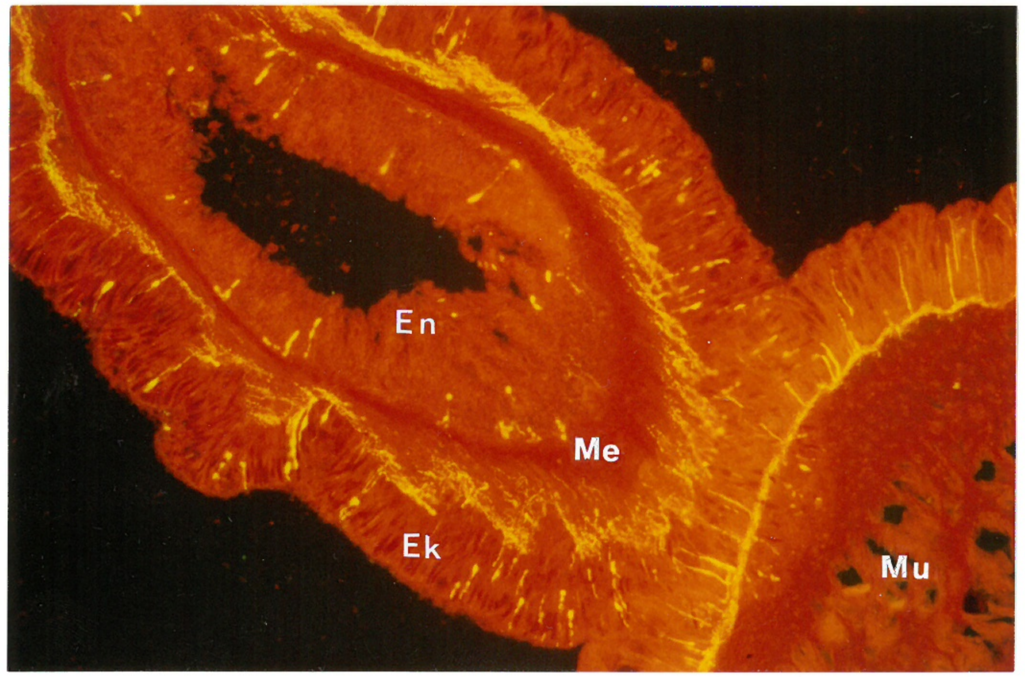

Abb. 3
Immuncytochemische Färbung mit einem Antiserum gegen Arg-Pro-NH_2. Lange, schlanke Neurone im (**a**) Ektoderm der Körperwand und im (**b**) Ekto- und Entoderm einer Tentakel der Seeanemone *Calliactis parasitica* werden angefärbt. Ek = Ektoderm, En = Entoderm, Me = Mesogloea, Mu = Mundscheibe; (a) x 290 , (b) x 115; (Fotos von Prof. Grimmelikhuijzen).
